# Diagnosis and management of community-acquired pneumonia in children: South African Thoracic Society guidelines

**DOI:** 10.7196/AJTCCM.2020.v26i3.104

**Published:** 2020-08-04

**Authors:** H Zar, D P Moore, S Andronikou, A C Argent, T Avenant, C Cohen, R J Green, G Itzikowitz, P Jeena, R Masekela, M P Nicol, A Pillay, G Reubenson, S A Madhi

**Affiliations:** ^1^ Department of Paediatrics and Child Health, Red Cross War Memorial Children’s Hospital and Faculty of Health Sciences, University of Cape Town, South Africa; South African Medical Research Council Unit on Child and Adolescent Health, University of Cape Town, South Africa; ^2^ Department of Paediatrics and Child Health, Chris Hani Baragwanath Academic Hospital, and Faculty of Health Sciences, University of the Witwatersrand, Johannesburg, South Africa; ^3^ Department of Paediatrics and Child Health, Red Cross War Memorial Children’s Hospital and Faculty of Health Sciences, University of Cape Town, South Africa; Department of Pediatric Radiology, Perelman School of Medicine, University of Philadephia, USA; ^4^ Department of Paediatrics and Child Health, Red Cross War Memorial Children’s Hospital and Faculty of Health Sciences, University of Cape Town, South Africa; ^5^ Department of Paediatrics and Child Health, Red Cross War Memorial Children’s Hospital and Faculty of Health Sciences, University of Pretoria, South Africa; ^6^ Centre for Respiratory Diseases and Meningitis, National Institute for Communicable Diseases, Johannesburg, South Africa; ^7^ Department of Paediatrics and Child Health, Faculty of Health Sciences, University of Pretoria, South Africa; ^8^ Department of Paediatrics and Child Health, Red Cross War Memorial Children’s Hospital and Faculty of Health Sciences, University of Cape Town, South Africa; ^9^ Department of Paediatrics and Child Health, Nelson R Mandela School of Medicine, School of Clinical Medicine, College of Health Sciences, University of KwaZulu-Natal, Durban, South Africa; ^10^ Department of Paediatrics and Child Health, Nelson R Mandela School of Medicine, School of Clinical Medicine, College of Health Sciences, University of KwaZulu-Natal, Durban, South Africa; ^11^ Division of Medical Microbiology, Department of Pathology, Faculty of Health Sciences, University of Cape Town, South Africa; and Division of Infection and Immunity, School of Biomedical Sciences, University of Western Australia, Perth, Australia; ^12^ Department of Paediatrics and Child Health, Nelson R Mandela School of Medicine, School of Clinical Medicine, College of Health Sciences, University of KwaZulu-Natal, Durban, South Africa; ^13^ Department of Paediatrics and Child Health, Rahima Moosa Mother and Child Hospital, Faculty of Health Sciences, University of the Witwatersrand, Johannesburg, South Africa; ^14^ South African Medical Research Council Vaccine and Infectious Diseases Analytics Unit, University of the Witwatersrand, Johannesburg, South Africa; Department of Science and Technology/National Research Foundation: South African Research Chair in Vaccine Preventable Diseases, Faculty of Health Sciences, University of the Witwatersrand, Johannesburg, South Africa

**Keywords:** covid-19, coronavirus

## Abstract

Background. Pneumonia remains a major cause of morbidity and mortality amongst South African children. More comprehensive immunisation regimens, strengthening of HIV programmes, improvement in socioeconomic conditions and new preventive strategies have impacted on the epidemiology of pneumonia. Furthermore, sensitive diagnostic tests and better sampling methods in young children improve aetiological diagnosis. Objective. To produce revised guidelines for pneumonia in South African children under 5 years of age. Methods. The Paediatric Assembly of the South African Thoracic Society and the National Institute for Communicable Diseases established seven expert subgroups to revise existing South African guidelines focusing on: (i) epidemiology; (ii) aetiology; (iii) diagnosis; (iv) antibiotic management and supportive therapy; (v) management in intensive care; (vi) prevention; and (vii) considerations in HIV-infected or HIVexposed, uninfected (HEU) children. Each subgroup reviewed the published evidence in their area; in the absence of evidence, expert opinion was accepted. Evidence was graded using the British Thoracic Society (BTS) grading system. Sections were synthesized into an overall guideline which underwent peer review and revision. Recommendations. Recommendations include a diagnostic approach, investigations, management and preventive strategies. Specific recommendations for HIV infected and HEU children are provided. Validation. The guideline is based on available published evidence supplemented by the consensus opinion of SA paediatric experts. Recommendations are consistent with those in published international guidelines.

*Afr J Thorac Crit Care Med.* Published online 30 July 2020. https://doi.org/10.7196/AJTCCM.2020.v26i3.

Community-acquired pneumonia forms part of a broad spectrum of acute lower respiratory tract illness (LRTI) in children. This terminology recognises that LRTI is a spectrum of illness ranging from airway to parenchymal disease, dependent on the pathogen/s and the host response.[1] As prevention and management strategies for childhood pneumonia have been strengthened, so reductions in pneumonia incidence, severity and shifts in aetiology have occurred. Besides the impact on under-5 mortality, pneumonia in early childhood may reduce lung function, setting a trajectory for long-term impairment of lung health including the development of asthma or chronic obstructive pulmonary disease (COPD) in adulthood.

In South Africa (SA), with socioeconomic improvements, reduction in perinatal HIV transmission, increasing numbers of HIV-exposed uninfected (HEU) children, effective combination antiretroviral therapy (ART) programmes, and improved immunisation, the epidemiology and aetiology of childhood pneumonia is changing.

In addition, improved diagnostic methods have highlighted the importance of multiple pathogens contributing to co-infection in the aetiology of respiratory illness, and the importance of organism interactions. Current treatment and preventive strategies for childhood pneumonia therefore require revision.

## Epidemiology

Pneumonia is one of the most common causes of morbidity and mortality in SA children, despite improvements in immunisation and HIV management programmes. In 2017 there were ~320 000 pneumonia episodes and 4 100 deaths in SA children aged <5 years.[2] The incidence of pneumonia in children <5 years old in SA has declined by ~50% from 2000 - 2015, including an estimated 71% reduction in children living with HIV (CLWH).[3] The roll-out of interventions to prevent mother-to-child transmission (PMTCT) of HIV and increased provision of ART to HIV-infected individuals has contributed to the decline in pneumonia cases.[4] Furthermore, following introduction of pneumococcal conjugate vaccine (PCV) into the SA public immunisation programme in 2009, PCV immunisation was estimated to have reduced under-5 hospitalisations for all-cause pneumonia by 33% and 39% in HIV-uninfected and HIV-infected children, respectively, by 2014.[5] Invasive pneumococcal disease has also declined by 69% among children <2 years, including an 89% reduction in PCV7 serotypes and a 57% reduction in PCV13 serotypes in 2012.[6] Despite these reductions, pneumonia remains one of the most important causes of death in SA children <5 years and is a major cause of healthcare utilisation and morbidity.[7,8] There are several determinants of pneumonia severity and mortality. The case fatality risk (CFR) among children hospitalised with pneumonia in 5 SA hospitals was 2% from 2009 - 2012,[8,9] with HIV infection an important risk factor for hospitalisation and in-hospital mortality.[10] HEU infants have an increased risk of hospitalisation compared to HIV-unexposed infants and an increased risk of in-hospital death, predominantly in the first 6 months of life.[11,12] Increased risk of hospitalisation in HEU infants has also been found for specific pathogens such as pneumococcus, respiratory syncytial virus (RSV) and influenza virus.[10,11,13] Additional risk factors for severe pneumonia include infancy (particularly those <4 months), premature birth, incomplete immunisation, maternal smoking or household tobacco smoke exposure, indoor air pollution, low birthweight, malnutrition, lack of exclusive breastfeeding and overcrowding.[9,14,15]

## Summary – epidemiology

1. In SA, pneumonia incidence in children has declined by around 50% from 2000 to 2015, with an estimated 70% reduction in episodes in HIV-infected children.
2. A reduction in vertical transmission of HIV, increased provision of ART, and PCV have contributed to this reduction.
3. Pneumonia remains a major cause of morbidity and death in SA children.
4. Risk factors for pneumonia hospitalisation include: HIV infection, HIV exposure (predominantly in infants), young age (particularly those <4 months), premature birth, incomplete immunisation, maternal smoking or household tobacco smoke exposure, indoor air pollution, low birthweight, malnutrition, non-exclusive breastfeeding and overcrowding.

## Diagnosis

### Clinical diagnosis

#### Clinical presentation

The main symptoms of pneumonia are cough, difficulty breathing or tachypnoea. Physical examination should include: assessment of the child's general appearance, measurement of respiratory rate, evaluation of the work of breathing and pulse oximetry. Auscultation of the chest should be done where possible (evidence level II); however, there is wide inter- and intra-observer variability in the interpretation of auscultatory sounds in paediatric pneumonia.[16]

The World Health Organization (WHO) guidelines classify children with cough or difficulty breathing into three categories, based on clinical signs – severe pneumonia, pneumonia or no pneumonia (evidence level Ia) (Fig. 1; Table 1).[17] Children with lower chest indrawing are now classified as having pneumonia, rather than severe pneumonia. Treatment is based on these categories – severe pneumonia requires referral to hospital and antibiotics; pneumonia requires oral antibiotics and outpatient management with follow-up; and no pneumonia is treated symptomatically. However, children living with HIV (CLWH), malnourished children or immunocompromised children, who present with lower chest indrawing, should be regarded as having severe pneumonia and referred to hospital for appropriate management (evidence level Ib).

#### Assessment of severity

Assessment of the **general appearance** of the child is helpful to evaluate severity of illness. Any child with a general danger sign requires

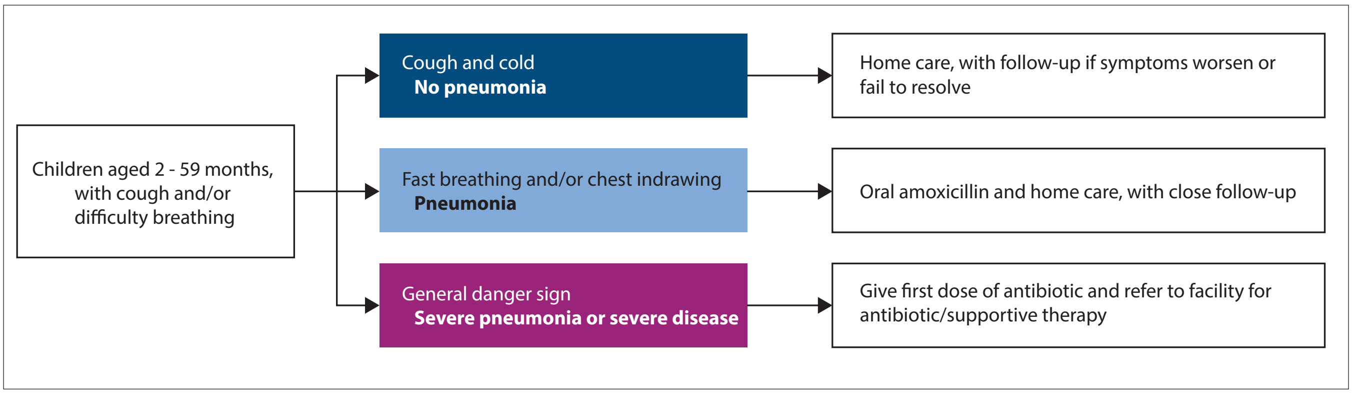

*Fig. 1. Revised World Health Organization classification and treatment of childhood pneumonia at health facilities.*[2]

**Table 1. Categories of pneumonia – World Health Organization classification**[2]

| Category | Characteristics |
|---|---|
| Severe pneumonia | **Any child with a general danger sign** |
| | Inability to drink |
| | Convulsions |
| | Abnormal sleepiness |
| | Persistent vomiting |
| | *or* |
| | Oxygen saturation <90% (at altitude >1 800 m) or <92% at sea level or central cyanosis |
| | *or* |
| | Severe respiratory distress (grunting, very severe chest indrawing) |
| | **Infant <2 months of age with** |
| | A general danger sign |
| | *or* |
| | Chest wall indrawing |
| | *or* |
| | Tachypnoea (≥60 breaths per min) |
| | **Children living with HIV, immune-compromised or malnourished children with** |
| | Lower chest indrawing |
| Pneumonia | **Child >2 months of age with** |
| | Lower chest indrawing |
| | *or* |
| | Tachypnoea |
| | ≥50 breaths per min for infants 2 - 11 months of age |
| | ≥40 breaths per min for children 1 - 5 years of age |
| No pneumonia | No signs of pneumonia or severe pneumonia, i.e. upper respiratory tract infection |

**Table 2. Indications for hospital admission**

| |
|---|
| All children <2 months of age |
| Children >2 months of age with |
| A general danger sign |
| Grunting, severe lower chest indrawing |
| Stridor in a calm child |
| Room air arterial oxygen saturation <92% at sea level or <90% at high altitude, or central cyanosis |
| Severe malnutrition |
| HIV-infected, immune-compromised or malnourished child with lower chest indrawing |
| Family unable to provide appropriate care |

referral to hospital. All children <2 months of age with signs of pneumonia require hospital admission (Table 2).

Excessive **work of breathing,** as indicated by grunting, nasal flaring or very severe chest wall indrawing, is a useful indicator of severity (evidence level Ia).[18,19] British Thoracic Society (BTS) guidelines recommend that signs indicating excessive work of breathing are more specific for diagnosing severe pneumonia than respiratory rate (evidence level II).[20]

Assessment of **oxygenation** is important as a measure of severity (evidence level Ia).[21] Pulse oximetry should be performed in all children, using a paediatric wrap-around probe (evidence level Ib). A saturation of <92% or <90% at higher altitudes (≥1 800 m) indicates the need for hospital admission and supplemental oxygen (evidence level Ia).[20,22]

### Radiological diagnosis

A chest X-ray (CXR) may be useful for confirming the presence of pneumonia or complications such as a lung abscess or empyema (Table 3). A CXR cannot accurately discriminate between viral and bacterial pneumonia (evidence level II).[20,22,23] Overall, a CXR does not influence outcome and rarely informs changes of treatment in the ambulatory setting (evidence level Ib).[23] There is also no evidence that a lateral CXR improves the diagnostic yield in children with pneumonia,[24] except for detection of hilar adenopathy if tuberculosis (TB) is suspected (evidence level II).[25,26]

The use of a CXR has several limitations, including radiographic features being masked by anatomical structures; a normal CXR in the early stages of pneumonia; and lack of inter-reader agreement in interpretation.[27] Clinician-led point-of-care ultrasound is increasingly being used, with higher inter-observer agreement than for a CXR (evidence level Ib).[27,28] Evidence suggests a similar or higher yield in the diagnosis of consolidation or pleural effusion when using ultrasound (evidence level Ib). However, ultrasound is not yet routinely available for the diagnosis of pneumonia, and a CXR remains the standard investigation.[12]

Computed tomography (CT) is not recommended as a first-line diagnostic tool, but where available, can be considered for detecting complications of pneumonia in the acute or subacute phase (for diagnosing a suppurative complication such as necrotising pneumonia, abscess or empyema) and in the chronic phase (for diagnosing bronchopleural fistula or detecting and localising bronchiectasis); it can also be useful for differentiating pneumonia from other pathological conditions, including endobronchial lesions/foreign bodies causing

**Table 3. Indications for chest X-ray (evidence level Ib)**

- Severe pneumonia
- Suspected pulmonary tuberculosis
- Suspected foreign body aspiration
- Pneumonia unresponsive to standard management
- Consider in children <5 years of age, presenting with fever (>39°C), leukocytosis and no obvious source of infection, as ~18% of such patients have radiographic pneumonia (evidence level III)[14,15]

atelectasis and for demonstrating previously undiagnosed, underlying congenital lesions.[20,22] Radiation dose is less of a concern, as low-dose scans (at doses of ~10 CXRs or 3 - 5 anteroposterior (AP) and lateral CXRs) can be performed.

#### Follow-up chest X-ray

A follow-up CXR after acute uncomplicated pneumonia is not indicated if there is clinical improvement (evidence level II).[22,29] A follow-up CXR at 2 - 4 weeks should be done: (*i*) in children with lobar collapse; (*ii*) to document resolution of a round pneumonia (as this may mimic the appearance of a Ghon focus); and (*iii*) in those with ongoing respiratory symptoms.[20,22] A chest ultrasound scan should be considered as an alternative to a repeat CXR in children with unresolving or worsening signs and symptoms to detect complications such as pleural effusion (evidence level Ib).[28,30,31]

### Aetiological diagnosis

Clinical assessment and chest radiography cannot determine the aetiology of pneumonia.[20,32-34] Diffuse bilateral wheezing is, however, often associated with a viral infection, especially respiratory syncytial virus (RSV) (evidence level Ib).[33] Various diagnostic modalities are available for aetiological diagnosis, such as microscopy, molecular diagnostics, culture and antigen detection (Table 4). Recent advances in understanding the aetiology have highlighted that pneumonia may be due to interactions or co-infection with several organisms, including viral-viral and viral-bacterial infections.[22,35] Testing of upper respiratory samples may not, however, discriminate between colonising and pathogenic organisms, making it difficult to attribute aetiology. Identification of organisms such as *Bordetella pertussis*, RSV, influenza virus, parainfluenza virus, severe acute respiratory syndrome coronavirus 2 (SARS-CoV-2) in children with clinical and/or radiological features of pneumonia, or *Mycobacterium tuberculosis* in upper respiratory samples among children is, however, strongly attributable to the aetiology of lower respiratory tract infection (evidence level Ia).[36,37]

The following should be considered when investigating the aetiology of lower respiratory tract infection:

- General tests for infection, including acute-phase reactants (erythrocyte sedimentation rate (ESR), C-reactive protein (CRP), white cell count (WCC), neutrophil count and procalcitonin (PCT)) do not reliably differentiate bacterial from viral pneumonia and should not be routinely used (evidence level Ib).[22,38] CRP concentrations ≥40 mg/L with radiological confirmation of pneumonia suggests bacterial pneumonia (evidence level II)[39]
- HIV status should be determined in all children requiring hospital admission for pneumonia
- Microbiological investigations on blood, pleural fluid or respiratory samples should only be done in children requiring hospital admission (i.e. in those with severe disease or complications or in outbreak situations) (evidence level IVa)[20,22]
- For detection of viruses, polymerase chain reaction (PCR) and/or immunofluorescence on nasal samples may be useful (evidence level Ib). Viruses strongly associated with pneumonia include RSV, influenza and parainfluenza virus and SARS-CoV-2 virus in symptomatic children. Detection of adenovirus, human metapneumovirus (HMPV) or rhinovirus, even though associated with pneumonia, should be interpreted with caution, as healthy children or those with upper respiratory tract infection (URTI) may have a positive test (evidence level Ia).[35,40] Testing for SARS-CoV-2 using PCR can be done on mid-tubinate nasal swabs
- Blood culture has a very low diagnostic yield. Antibiotic pre-exposure and specimen volume impact on blood culture yield.[41] Overall, ~5% of blood cultures of suspected bacterial pneumonia cases are positive; the yield is higher in severe pneumonia[42,43] and in CLWH[44]
- Induced sputum provides a higher yield than upper respiratory secretions for *B. pertussis, Pneumocystis jirovecii* and *M. tuberculosis*[45-49]
- Pulmonary TB should be considered in a child presenting with severe pneumonia or penumonia with a known TB contact, if the tuberculin skin test is positive, if the child is malnourished or has lost weight, and in CLWH or in those who are HIV-exposed (evidence level 1b).[50-52] Two sequential respiratory samples, preferably expectorated or induced sputum, should be tested with Xpert MTB/RIF Ultra (Cepheid, USA) and mycobacterial culture with drug susceptibility testing (evidence level Ib)[53]
- Pleural fluid can be tested for microscopy, culture, pneumococcal antigen (by latex agglutination), PCR for bacteria, mycobacterial culture and Xpert MTB/RIF Ultra (evidence level II).

### Section summary: Diagnosis

1. A diagnosis of pneumonia should be considered in any child who has an acute onset of cough, fast breathing or difficulty breathing.
2. Revised WHO guidelines classify children with cough or difficulty breathing into: (*i*) severe pneumonia; (*ii*) pneumonia; and (*iii*) no pneumonia. Malnourished or immunocompromised children with lower chest indrawing should be managed as severe pneumonia (evidence level Ib).
3. Excessive work of breathing, as indicated by grunting, nasal flaring or severe chest wall indrawing, is an important indicator of severity (evidence level Ia).
4. Pulse oximetry should be performed on all children, with referral to hospital for oxygen if saturation is <92% at sea level or <90% at an altitude >1 800 m (evidence level Ia).

**Table 4. Summary of investigations in children hospitalised for pneumonia**[12]

| | Advantages | Disadvantages |
|---|---|---|
| Vital signs | | |
| Pulse oximetry | Accurate measure of hypoxaemia; guides the use of supplemental oxygen | - |
| Radiological tests | | |
| Chest X-ray | Assess extent of pneumonia<br>Detect complications | Unable to distinguish aetiology<br>Poor intra- and inter-observer agreement for interpretation of some features |
| Lung ultrasound | Higher inter- and intrapersonal agreement of radiological findings compared with CXR<br>May be more sensitive than CXR for detecting abnormalities<br>Easily repeatable, no radiation<br>Can be done by non-radiologists with minimal training | Not widely available<br>Few clinicians have expertise in its use |
| Blood | | |
| Culture for bacterial pathogens | Relative ease of collection<br>Positive culture with a clinically significant pathogen has high specificity<br>Able to guide empirical antibiotic susceptibility patterns | Low sensitivity; therefore, high cost per case detected |
| Molecular testing | More sensitive than blood culture for some targets, e.g. pneumococcal *lytA*<br>Useful for CMV viral load | Lacks specificity for disease, e.g. *lytA* detection may reflect pneumococcal carriage |
| Serology | Useful for epidemiological studies and for specific pathogens, e.g. *B. pertussis* | Usually requires acute and convalescent sera; therefore, not useful for guiding acute treatment decisions |
| Biomarker detection | Potential to discriminate bacterial vs. viral infection | Accuracy for distinguishing bacterial vs. viral pneumonia is suboptimal for available biomarkers (CRP, ESR and PCT) |
| HIV infection | HIV testing essential in hospitalised children whose HIV status is unknown<br>HIV infection or HIV exposure may impact on the spectrum of pathogens considered in empirical antibiotic therapy | - |
| Nasopharyngeal or nasal swab or aspirate | | |
| Bacterial culture, molecular or antigen detection of bacteria and viruses | Ease of collection, relatively good correlation of results with sputum testing, method of choice for some viruses (e.g. RSV, influenza, para-influenza virus, SARS-CoV-2), bacteria (*B. pertussis*) and *P. jirovecii* | Colonisation or infection of the upper airway does not imply that organisms are causing pneumonia<br>Predictive value of attributing causality is high for RSV, influenza virus, para-influenza virus 3 and *M. tuberculosis*<br>Limited value for most other bacteria and viruses |
| Sputum (expectorated or induced) | | |
| Bacterial culture, molecular or antigen detection of bacteria (*M. tuberculosis, B. pertussis*) or *P. jirovecii* | Relative ease of collection<br>Incremental yield over testing of upper respiratory samples for *M. tuberculosis, B. pertussis* and *P. jirovecii* | Requires expertise, and should be conducted in a dedicated space that is well ventilated<br>May also detect organisms colonising or infecting upper airway |
| Urine antigen testing | | |
| Antigen detection | Relative ease of collection | Poor specificity for pneumococcal disease in children due to high prevalence of nasopharyngeal carriage |
| Tracheal aspiration or bronchoalveolar lavage | | |
| Bacterial culture, molecular or antigen detection of bacteria, *P. jirovecii* and viruses | More representative of organisms in the lower respiratory tract<br>Less likely to be contaminated by upper respiratory tract flora | Few comparative studies vs. other sample types<br>Costly, invasive, requires expertise |

**Continued ...**

**Table 4. (continued) Summary of investigations in children hospitalised for pneumonia[12]**

| | Advantages | Disadvantages |
|---|---|---|
| Percutaneous lung aspiration | | |
| Bacterial culture, molecular or antigen detection of bacteria and viruses | Most representative of lower respiratory tract, least contamination with upper airway respiratory tract flora | Useful mainly for peripheral infective foci in the right lung<br>Invasive, and requires expertise<br>Small risk of serious complications |

CXR = chest X-ray; CMV = cytomegalovirus; *B. pertussis* = *Bordetella pertussis;* CRP = C-reactive protein; ESR = erythrocyte sedimentation rate; PCT = procalcitonin; RSV = respiratory syncytial virus; *P. jirovecii* = *Pneumocystis jirovecii; M. tuberculosis* = *Mycobacterium tuberculosis.*

5. A CXR should not be done routinely (evidence level Ib), but should be performed in severe cases to confirm pneumonia and detect complications or when TB is suspected.
6. A follow-up CXR should only be done if the condition of a child does not improve or complications are suspected (evidence level II).
7. Evidence for point-of-care chest ultrasound for diagnosis is accumulating. A chest ultrasound scan, rather than a repeat CXR, should be considered in children with ongoing symptoms (evidence level II).
8. CRP ≥40 mg/L with radiological confirmation of pneumonia is supportive of a bacterial aetiology (evidence level II).
9. Microbiological investigations should not be performed routinely on children, but only in those requiring hospitalisation or in outbreak settings (evidence level IVa).
10. In children with pneumonia, testing of nasal samples with PCR is useful for detecting RSV, influenza virus, parainfluenza virus or SARS-CoV-2; other viruses should be cautiously interpreted, as healthy children or those with URTI may have a positive test.
11. Induced sputum may provide a higher yield than upper respiratory samples for *B. pertussis*, *P. jirovecii* and *M. tuberculosis*.
12. Investigations for TB should be done in children with severe pneumonia or pneumonia with a history of a TB contact, a positive tuberculin skin test, loss of weight or malnutrition or if HIV-infected.

### Aetiology diagnosis

A wide spectrum of pathogens, ranging from viral and bacterial to fungal and parasitic organisms, is implicated in pneumonia pathogenesis, and disease may be due to multiple organisms (evidence level Ia). If children are hospitalised with pneumonia, bacterial-viral co-infection is often the norm (evidence level Ib).[54,55] In the current era of conjugate vaccines targeting *Haemophilus influenzae* type b (Hib) and *Streptococcus pneumoniae*, there has been a shift in the spectrum of pathogens causing pneumonia, with viruses contributing to a greater proportion of severe LRTI episodes (evidence level Ib) and non-typable *H. influenzae* and *S. aureus* emerging as important bacterial pathogens.[48,49,56-60]

#### Bacterial organisms

The Drakenstein Child Health Study and the Pneumonia Etiology Research for Child Health (PERCH) case-control studies indicate that non-typable *H. influenzae* and *S. aureus* are now the leading bacterial causes of severe pneumonia in SA children (Supplementary Table 1 - http://ajtccm.org.za/public/sup/104.pdf) (evidence level Ib).[48,61,62]

Children presenting with empyema or pleural effusion, confluent dense consolidation or pneumatocoele on CXR associated with high-grade pyrexia and elevation of acute phase reactants (e.g. CRP), or those with features of suppurative lung disease, are highly likely to have a bacterial cause (evidence level Ib).[63,64] The most frequently isolated bacteria are *S. aureus,* non-typable *H. influenzae*, *S. pneumoniae* and *Streptococcus pyogenes* (Group A *Streptococcus*).[63,65]

Gram-negative enteric organisms, particularly *Klebsiella pneumoniae*, *Escherichia coli, Enterobacter cloacae* and *Salmonella* spp. are important pneumonia pathogens in HIV-infected or malnourished children in sub-Saharan Africa (evidence level Ib).[44,66,67] In the Child Health and Mortality Prevention Surveillance (CHAMPS) Program, a study designed to establish the infectious aetiology of fatal illness in children dying in hospital, *K. pneumoniae* was identified in 16% of community-acquired pneumonia deaths, and the most common organism isolated in death from hospital-acquired infection in Soweto.[68] Non-fermenting Gram-negatives, such as *Moraxella catarrhalis* or *Pseudomonas aeruginosa* are increasingly recognised as contributors to bacterial pneumonia in children (evidence level II).

*Bordetella pertussis* is an important cause of pneumonia in young infants who are unimmunised or incompletely immunised. In Cape Town, *B. pertussis* prevalence among young children hospitalised with pneumonia was 16% in CLWH, 11% in HEU and 5% in HIV-unexposed children.[47] In the PERCH study, *B. pertussis* was detected in 2% of HIV-uninfected and 1% of HIV-infected children hospitalised with severe or very severe pneumonia.[61,62]

#### Tuberculosis

*M. tuberculosis* is increasingly recognised as an important pathogen in acute pneumonia in settings with a high burden of tuberculosis and HIV (evidence level Ib). In SA, 43 - 85% of children with culture-confirmed tuberculosis presented with acute cough (<14 days) (evidence level Ib).[50,67,69,70] In the Drakenstein study, the incidence of tuberculin skin test (TST) conversion was 12 per 100 child years, and that of pulmonary tuberculosis 2.9 per 100 child years (evidence level Ib).[71] In the PERCH study, 3% of SA children hospitalised with WHO-defined severe pneumonia had microbiologically confirmed tuberculosis (evidence level Ib).[53] Given the suboptimal sensitivity of mycobacterial culture, the proportion of SA paediatric pneumonia cases associated with *M. tuberculosis* is likely to be ~7 - 15% (evidence level IVa).[61,62,71]

If pulmonary tuberculosis is diagnosed, the child must be notified and treatment should be initiated in accordance with the South African Guidelines for the Management of Tuberculosis in Children (2013; https://health-e.org.za/2014/06/10/guidelines-management-tuberculosis-children/ https://health-e.org.za/wp-content/uploads/2014/06/National-Childhood-TB-Guidelines-2013.pdf)

### Respiratory viruses

Respiratory viruses are the leading cause of pneumonia and of hospitalisation in young children (evidence level Ia).[9,58,72] Respiratory virus-related illness follows predictable seasonal shifts; in SA this season is between March and September (autumn to early spring) (evidence level II).[73,74] Respiratory syncytial virus causes 18 - 31% of pneumonia episodes (evidence level Ia).[35] Other viruses associated with pneumonia include influenza, parainfluenza, HMPV, human rhinovirus and adenovirus (Supplementary Table 2 - http://ajtccm.org.za/public/sup/104.pdf) (evidence level Ib).[9,48,49]

Respiratory viruses are diverse, continually adapting and prone to causing intermittent epidemics and pandemics, particularly when adapting from animal reservoirs to the human host. Numerous pandemic influenza events occurred in the 20th century,[75] and the pandemic 2009 H1N1 influenza strain was implicated in the first influenza pandemic of the 21st century. Coronaviruses, too, have been associated with epidemics in recent decades.[76-79] Highly virulent influenza and coronavirus outbreaks may be associated with considerable case fatality risk, and it is for this reason that all cases of severe acute respiratory syndrome in which no alternative aetiological diagnosis is made be investigated for novel respiratory viruses. Such cases constitute a Category 1 Notifiable Medical Condition in SA (https://www.nicd.ac.za/wp-content/uploads/2017/06/NMC-list_2018.pdf).

Although novel coronavirus disease 2019 (COVID-19) appears to affect children mildy, with most developing asymptomatic or mild illness,[80] children with acute lower respiratory illness requiring hospitalisation should be tested for SARS-CoV-2 through the epidemic. Evidence on COVID-19 in children is rapidly evolving and current management of paediatric cases should defer to recommendations from the National Department of Health (https://www.nicd.ac.za/diseases-a-z-index/covid-19/covid-19-guidelines/). Children infected with SARS-CoV-2 may present with non-respiratory illness including multisystem inflammatory disease; and so, where resources permit, hospitalised children may be screened for infection, especially if there is a history of contact with a positive adult.

Viral-viral co-infections and viral-bacterial co-infections are increasingly recognised in the pathogenesis of severe pneumonia (evidence level Ib).[81] Preceding respiratory viral infection may prime the respiratory tract for new acquisition of bacterial colonisation, and increase in density of colonisation or vice versa (evidence level Ib).[33,82,83]

Measles and varicella-zoster virus occasionally cause severe, or fatal, pneumonia in the context of outbreaks or epidemics (evidence level II). Typically, malnourished or immunocompromised children tend to develop the most severe forms of pneumonia related to these organisms.[84,85] Children hospitalised with measles or varicella-zoster virus-associated pneumonia frequently develop superimposed bacterial pneumonia (primarily *S. aureus* and *S. pyogenes*) and require treatment with broad-spectrum antibiotic therapy (evidence level IVa).[86-88]

#### Opportunistic organisms

*Pneumocystis jirovecii*, either alone or as a co-pathogen with cytomegalovirus (CMV), is a leading infection in CLWH not on ART in SA (evidence level Ib).[62,89-92] Typically, pneumocystis pneumonia (PCP) affects young infants, between 6 weeks and 6 months of age; presentation is with severe hypoxia, dyspnoea, low-grade fever and normal chest auscultation (evidence level Ib).[93] However, *P. jirovecii* also commonly colonises the airways in infants.

The association of CMV alone with severe childhood pneumonia in HIV-infected and HEU children is contentious (evidence level II),[94,95] although histological evidence of CMV pneumonia is frequently seen in HIV-infected children dying of pneumonia.[96] Fatal disseminated disease associated with CMV is also well described in children who are solid organ or bone marrow transplant recipients.[97,98]

#### Atypical bacteria

*Chlamydophila pneumoniae*, *Chlamydia trachomatis*, *Mycoplasma pneumoniae* and *Legionella* spp. are infrequently associated with pneumonia in SA children (evidence level II),[48,61,62,99] but should be considered in the differential diagnosis in children with progressive respiratory failure, despite broad-spectrum antibiotic cover (evidence level IVa). Age <5 years and HIVinfection were associated with hospitalisation for severe *M. pneumoniae* pneumonia in SA.[100]

### Summary – aetiology

1. Co-infections are common in pneumonia pathogenesis.
2. The most frequently isolated bacterial pathogens following PCV and Hib immunisation are non-typable *H. influenzae* and *S. aureus*.
3. *B. pertussis* is implicated in severe pneumonia aetiology in those with incomplete immunisation.
4. *M. tuberculosis* is an important pathogen in settings with a high burden of tuberculosis.
5. Gram-negative bacteria are important pathogens in HIV-infected and malnourished children.
6. Respiratory viruses, particularly RSV, are responsible for most pneumonia episodes. Influenza, parainfluenza, adenovirus and human bocavirus, HMPV and rhinovirus are common, although not always pathogenic.
7. In HIV-infected children not on ART, *P. jirovecii* either alone or as a co-pathogen with CMV, is an important opportunistic infection.

## Treatment of childhood pneumonia

### Antibiotic treatment

Choice of empiric antibiotic treatment depends on the child's age, possible aetiology, antimicrobial resistance patterns, previous treatment, as well as factors affecting host susceptibility, including HIV, nutritional and vaccination status. All children with signs of pneumonia or severe pneumonia should receive antibiotics (evidence level Ib).[17,101]

#### Adaptation of guidance to address antibiotic resistance

Substantial (>80%) reductions in the incidence of invasive pneumococcal disease were observed within 4 years of pneumococcal conjugate vaccine (PCV) introduction.[3] High-dose amoxicillin is effective against pneumococci with low- and intermediate-level penicillin non-susceptibility causing pneumonia. Empiric therapy for hospitalised children with community-acquired pneumonia (CAP) should cover non-typable *H. influenzae* and *S. aureus*.[61,62]

#### Which empiric antibiotic?

For severe pneumonia in children >1 month of age, amoxicillin-clavulanate (90 mg/kg/day of amoxicillin component) is recommended. Oral therapy (45 mg/kg/day 12-hourly of amoxicillin component) is preferable, but intravenous therapy (30 mg/kg/dose of intravemous amoxicillin component 8-hourly) should be initiated in children who are unable to tolerate oral medications, if there is a concern about oral absorption, or if the child is severely ill.

For children <1 month of age, initial therapy should be intravenous ampicillin (40 mg/kg/dose 6-hourly) and gentamicin (7.5 mg/kg/dose daily) to cover common neonatal pathogens, including Listeria spp. (evidence level II). Group B Streptococcus, *S. aureus*, *Chlamydia trachomatis* and viruses should also be considered as causes of neonatal pneumonia. Consideration should be given to broadening cover if there is no clinical improvement within 48 hours of initiation of therapy.

A macrolide should be included when 'atypical' pathogens (e.g. Mycoplasma spp., Chlamydophila spp., pertussis) are suspected (evidence level IVa).

For ambulatory treatment of pneumonia, amoxicillin (45 mg/kg/dose 12-hourly) remains the preferred antibiotic for children >1 month old. Outpatient management should not be considered for infants <1 month of age (Table 5).

HIV infection or exposure influences investigation and management of children with pneumonia (see below: Special circumstances: HIV infection or exposure).

### Route of administration

Oral therapy is preferable; however, parenteral antibiotics should be used for children requiring intensive care unit (ICU) admission or for those too ill to tolerate oral medication. There are, however, risks and costs associated with intravenous use, and oral de-escalation is recommended as soon as feasible (evidence level IIIa).[102,103]

### Duration of antimicrobial therapy

In general, 5 days of antibiotic therapy is recommended, but longer duration may be needed in children with severe or complicated disease,

**Table 5. Empiric antibiotic therapy**

| Age | Outpatients | Inpatients |
|---|---|---|
| 0 - 1 month | Children <1 month of age should be hospitalised | Ampicillin 50 mg/kg IV 6-hourly,<br>or benzylpenicillin 50 000 U/kg IM/IV 6-hourly<br>and gentamicin 7.5 mg/kg IM/IV daily<br><u>If poor response</u><br>Ceftriaxone 50 mg/kg IV 12-hourly × 5 d *or*<br>Cefotaxime 50 mg/kg IV 8-hourly × 5 d<br><u>If cultures are negative</u>, switch to oral amoxicillin-clavulanate when clinically improving and taking well orally – complete a total antibiotic duration of 5 d<br><u>If cultures are positive</u>, use targeted therapy according to the organism's susceptibility pattern<br>Step down to oral antibiotic therapy as soon as the patient is clinically stable<br><br>**Add**<br>Azithromycin 10 mg/kg daily orally × 5 d if *C. trachomatis* is suspected (alternative: clarithromycin 7.5 mg/kg/d orally 12-hourly × 5 d; erythromycin is contraindicated in this age group) |
| >1 month | Amoxicillin 45 mg/kg/dose 12-hourly orally × 5 d<br><u>If poor response</u><br>Amoxicillin-clavulanate 45 mg/kg/dose 12-hourly × 5 d<br><br>**Add**<br>Azithromycin 10 mg/kg orally daily × 5 d if *M. pneumoniae, C. pneumoniae* or *C. trachomatis* suspected (alternatives: clarithromycin 7.5 mg/kg/d orally every 12 h for 10 d or erythromycin 50 mg/kg/d for 10 - 14 d) | Amoxicillin-clavulanate 30 mg/kg/dose (of amoxicillin component) 8-hourly IV × 5 d *or*<br>Amoxicillin-clavulanate 45 mg/kg/dose orally 12-hourly × 5 d<br><u>If cultures are positive</u>, use targeted therapy according to the organism's susceptibility pattern<br>Step down to oral antibiotic therapy as soon as the patient is clinically stable<br><u>For susceptible *S. aureus*</u>, use<br>Flucloxacillin 50 mg/kg orally 6-hourly × 2 - 4 weeks<br><u>If poor response</u><br>Ceftriaxone 50 mg/kg IV 12-hourly × 5 d *or*<br>Cefotaxime 50 mg/kg IV 8-hourly × 5 d<br><br>**Add**<br>Vancomycin 10 - 20 mg/kg/dose 6-hourly *or*<br>Clindamycin for suspected CA-MRSA 1 month - 16 years:<br>20 - 40 mg/kg IV or IM/d, in 3 - 4 equally divided doses<br>Use higher doses for treatment of more severe infections<br><br>**Add**<br>Azithromycin 10 mg/kg orally daily × 5 d if *M. pneumoniae*, *C. pneumoniae* or *C. trachomatis* suspected (alternative: clarithromycin or erythromycin) |

IV = intravenous; IM = intramuscular; CA-MRSA = community-acquired methicillin-resistant *S. aureus*.

if there is a poor response to therapy, or as informed by microbiology results.

Bacteraemic staphylococcal pneumonia should be treated for 14 - 28 days, dependent on complications and response to treatment.[104] Uncomplicated presumed staphylococcal pneumonia (i.e. blood culture negative, but with suggestive clinical or CXR features) may be appropriately managed with a 7- to 10-day course of targeted antibiotic therapy, depending on clinical response (evidence level IVa).[105]

### Management of a child who is not responding to therapy

A poor response to treatment has many possible explanations. Consider infection with *M. tuberculosis*, viruses, fungi or atypical organisms. Evaluate for the presence of a foreign body, empyema, heart disease or underlying immunodeficiency.[106] HIV infection is by far the most common immunodeficiency state to consider in SA children with recurrent or severe episodes of pneumonia. If HIV infection is excluded through age-appropriate testing, primary immunodeficiencies should be considered in the differential diagnosis.

Change to amoxicillin-clavulanate if there is a poor clinical response or deterioration in a child treated with amoxicillin. For children initially treated with amoxicillin-clavulanate, change to ceftriaxone (evidence level IVa).[17] Where laboratory support is available, it is strongly recommended to repeat a microbiological work-up, including blood culture, before changing antibiotics.

### Summary: Antibiotic therapy for community-acquired pneumonia

1. Oral amoxicillin is recommended for children >1 month of age who do not require hospitalisation (evidence level Ia).
   a. Treatment duration should be 5 days, with review after 3 days to evaluate response.
   b. Switch to amoxicillin-clavulanate if there is clinical deterioration, and consider referral for further investigation.
2. Children <1 month of age should be hospitalised and treated with ampicillin and an aminoglycoside (evidence level Ib).
3. Amoxicillin-clavulanate (intravenously or orally) is recommended for treatment of hospitalised children >1 month of age with severe pneumonia (evidence level IVa).
   a. Treatment duration should be 5 days.
   b. If there is clinical deterioration, switch to ceftriaxone or cefotaxime for 5 days (evidence level IVa).
4. Treatment duration should be prolonged for severe or complicated disease, and depend on microbiology testing.
   a. Bacteraemic *S. aureus* pneumonia may require 14 - 28 days of antibiotic therapy, depending on clinical response (evidence level IVa).
5. Macrolide antibiotics should be used if pertussis, mycoplasma or chlamydia is suspected (evidence level IVa).

### Adjunctive therapies

#### Antiviral treatment

Oseltamivir is of limited benefit and is not recommended for routine use. Consider use during the influenza season in children at high risk for severe influenza, who present soon after symptom onset.[8,24-36]

#### Corticosteroid therapy

Corticosteroids should be used in children with suspected or confirmed pneumocystis pneumonia (PCP) (see below: Special circumstances: HIV infection or exposure – Treatment), or in pulmonary tuberculosis with nodal airway compression and obstruction.[107,108]

#### Vitamin and micronutrient supplementation

**Vitamin A**

Vitamin A supplementation reduces severity of respiratory complications of measles,[109] but is not recommended for routine use.[110] Consider routine vitamin A supplementation for HIV-infected or malnourished children with CAP.[111]

**Vitamin D**

Vitamin D supplementation does not appear to improve CAP outcomes and is not routinely recommended (evidence level Ia).[112-115]

### Summary: Adjuvant therapies

1. Oseltamivir should be considered as early empiric therapy in children at risk of severe influenza-related pneumonia, who are hospitalised during the influenza season (evidence level II).
2. Routine use of corticosteroids for childhood CAP is discouraged (evidence level Ib).
3. Vitamin A is indicated for measles-associated pneumonia, or in those with vitamin A deficiency (evidence level Ib).
4. Do not routinely use vitamin D supplementation (evidence level Ia).

### When can a hospitalised child be discharged?

Apyrexial children no longer requiring oxygen, with adequate oral intake and acceptable home circumstances, can generally be safely discharged (Table 6).[22]

**Table 6. Criteria for discharge from hospital***

- Clinical improvement, indicated by improved activity, appetite, and resolution of fever for at least 12 hours. Do not discharge if increased work of breathing or tachycardia
- Pulse oximetry measurements consistently ≥90% at altitude (≥1 800 m) or ≥92% at sea level in room air for at least 12 hours
- Stable and/or return to baseline mental status
- If a chest tube was placed, no intrathoracic air leak for at least 12 - 24 hours after removal of the tube
- Ability to administer antibiotics at home, and child able to tolerate oral feeding and antibiotics
- Acceptable home circumstances and ability to return to hospital if clinical deterioration

*Adapted from the Pediatric Infectious Diseases Society and the Infectious Diseases Society of America guidelines.[20]

### General and supportive measures

#### Oxygen therapy

Assess oxygenation with regular pulse oximetry. If <92% at sea level (or <90% at altitude ≥1 800 m), administer oxygen via nasal cannula or face mask to maintain oxygen saturation 92 - 94% (evidence level II).[116] If pulse oximetry is unavailable, administer oxygen if there is central cyanosis, grunting, restlessness, inability to drink or feed, or if respiratory rate is ≥70/breaths per minute.[117]

#### Respiratory support

High-flow humidified nasal oxygen (HFHNO) or nasal continuous positive airway pressure (nCPAP) systems provide support to children with severe respiratory disease (see below: Care of the child with pneumonia in the paediatric ICU or high care).[118,119] These can be safely provided in adequately staffed and equipped high-care areas and district hospitals.[119,120]

#### Blood transfusion

In general, children who are haemodynamically stable should not be transfused if the haemoglobin (Hb) level is ≥7 g/dL (evidence level Ib); if their Hb is <5 g/dL, then transfuse packed red cells to raise the Hb to above the transfusion threshold (i.e. not to 'normal ranges') (evidence level II). For children with Hb 5 - 7 g/dL, evaluate their overall clinical status when deciding whether to transfuse.

#### Fluids and electrolytes

Fluid overload is associated with worse outcomes in severe CAP, particularly in those undergoing mechanical ventilation.[121-123] Hyponatraemia is common secondary to high antidiuretic hormone secretion and is related to the severity of infection,[124-128] but is less likely with isotonic intravenous manintenance fluids.[129]

Generally, children should be fed enterally; if this is not possible, then intravenous isotonic fluids should be administered at <80% of maintenance, with monitoring of sodium levels.

#### Antipyretics and analgesia

Hospitalised children with pneumonia, who often have fever and may have chest pain, should receive treatment,[130,131] including:[132]

- paracetamol, orally (loading dose 20 mg/kg/dose, then 15 mg/kg/dose 4- to 6-hourly)
- ibuprofen, orally (10 mg/kg/dose 8-hourly) with meals
  - where an anti-inflammatory effect is required
  - can be used in combination with paracetamol or opioids
- tilidine (1 drop per 2.5 kg body weight, i.e. 1 mg/kg/dose)
  - intermediate-efficacy opioid.

### Other measures

Over-the-counter cough medications are not effective in the management of CAP (evidence level Ia).[133]

Chest physiotherapy may be of benefit for children with lobar collapse,[134] or when used in conjunction with nebulisation;[135] however, routine chest physiotherapy is not recommended (evidence level Ia).[136,137]

Vaccination status should be reviewed and catch-up provided, including booster immunisation, as indicated.

### Summary: Treatment of pneumonia – supportive measures

1. Children with room air oxygen saturations of <92% (at sea level) or <90% (at altitude ≥1 800 m) should be treated with oxygen (evidence level II).
2. Most children may receive oxygen via nasal cannula, but the route of oxygen administration should be individualised (evidence level IVa).
3. Children who are haemodynamically stable should not be transfused if their Hb is ≥7 g/dL (evidence level II).
4. Children should be fed enterally; if this is not possible, administer intravenous isotonic fluids at <80% of maintenance with monitoring of sodium levels (evidence level Ib/II).
5. Children with fever or chest pain should be treated with appropriate antipyretics or analgesics (evidence level IVa).
6. Over-the-counter cough medications are not recommended (evidence level Ia).
7. Chest physiotherapy may benefit children with lobar collapse (evidence level Ia).
8. Vaccination status should be reviewed and catch-up provided, including booster immunisation, as indicated (evidence level IVb).

### Care of the child with pneumonia in the paediatric intensive care or high-care unit

#### Introduction

A proportion of hospitalised children with CAP require admission to high care or the paediatric ICU (PICU); many of them have comorbid disease or other underlying susceptibilities.

#### Admission criteria

Specific criteria for PICU admission depend on available resources and vary between institutions (Table 7).

#### Investigations

##### Microbiology

See 'Aetiological diagnosis' and 'Aetiology of pneumonia in children'.

**Table 7. Indications for paediatric intensive care unit admission**

- Rapidly deteriorating clinical condition despite appropriate management
- Need for respiratory support as evidenced by
  - apnoea (particularly in small infants)
  - increasing oxygen requirements, i.e. any child requiring $FiO_2$ >60% to maintain arterial saturations >88%[43-45]
  - increasing effort of breathing (as assessed by respiratory rate, chest-wall retractions, noisy breathing), with imminent respiratory collapse
  - hypercarbia resulting in respiratory acidosis
- Deterioration in level of consciousness or seizures, particularly if any concern about maintaining airway patency and avoiding aspiration
- Cardiovascular instability as reflected by severe tachycardia/hypotension/inotrope requirement

#### Radiological investigations

These should be individualised to evaluate for complications (e.g. pleural effusion, pneumothorax or segmental/lobar collapse), and associated cardiac disease. CXR or point-of-care chest ultrasound, where available, is recommended on admission to the PICU to define central line positioning, following endotracheal intubation, or with any significant deterioration.[138]

### Oxygen therapy and monitoring

Clinical signs are inadequate for detecting hypoxia;[139] therefore, continuous pulse oximetry monitoring is required.

### Respiratory support

#### High-flow oxygen

See above: General and supportive measues: Respiratory support.

#### Nasal continuous positive airway pressure systems nCPAP use for children with severe pneumonia (including bronchiolitis) is increasing as evidence emerges that supports its safety and efficacy.[140-144]

#### Invasive ventilation

Invasive ventilation is best provided in units experienced in such care, but often needs to be initiated prior to referral. High-frequency oscillatory ventilation may be considered for children requiring high mean airway pressure (MAP) using conventional ventilation.[145]

Children with significant airflow obstruction or hypercapnia require special consideration. Higher positive end-expiratory pressure (PEEP) and MAP may be required for children with refractory hypoxia (evidence level II).[146]

### Nutrition

Critically ill children should be provided with enteral nutrition as soon as possible (evidence level Ib).[147,148]

Many ventilated children receive inadequate dietary intake, and provision of a higher proportion of prescribed dietary goals is associated with improved outcomes.[149] Parenteral nutrition administration is associated with higher mortality and increased complications (evidence level Ib).[149-151]

Children on HFHNO or non-invasive positive-pressure ventilation may receive enteral feeding without risk of aspiration.[152,153]

### Antibiotic therapy

While PICU-specific issues should be considered, the principles of antibiotic therapy are similar as for other children with CAP. Consider broader antimicrobial therapy for children whose clinical status worsens despite initial empiric therapy. Therapy should be de-escalated based on microbiology investigations. Extrapolating from adult ICU evidence, procalcitonin levels may guide discontinuation of antibiotics,[154] although there are limited paediatric data.[155]

### Corticosteroids, fluid and blood transfusion

These should be administered as per children with CAP managed outside of the ICU (see above: Adjunctive therapies: Corticoisteroid therapy).

### Physiotherapy

Current evidence is insufficient to provide strong recommendations for chest physiotherapy in the PICU.[156,157]

## Summary: High care and intensive care of children with pneumonia

1. Where possible, blood cultures should be obtained from children requiring PICU admission, but should not delay initiation of antibiotic therapy (evidence level III).
2. CXR or chest ultrasound should be done to identify complications at PICU admission and after interventions, such as endotracheal intubation, chest drain or central line placement (evidence level III), and after clinical deterioration (evidence level III).
3. Oxygen saturation levels should be monitored continuously (evidence level IVa). Where possible, $FiO_2$ should be adjusted to achieve saturations of 92 - 96% (evidence level III).
4. nCPAP improves outcomes compared with nasal cannula oxygen (evidence level Ib), while nCPAP and HFHNO have similar efficacy in patients with severe bronchiolitis (evidence level Ib).
5. Antibiotic therapy should be de-escalated and discontinued as soon as possible (evidence level II).
6. Routine chest physiotherapy should not be provided for children (evidence level III), although some patients may benefit. Ongoing chest physiotherapy should be based on clinical improvement and lack of clinical deterioration (evidence level III).

## Prevention of childhood pneumonia

### General preventive strategies

General preventive strategies that reduce the incidence and severity of pneumonia are the following, and are summarised in Table 8:

#### Nutrition

Adequate nutrition and growth monitoring should be encouraged, as malnutrition predisposes children to pneumonia and severe illness. Breastfeeding has been shown to decrease the incidence of pneumonia in young children by up to 32%.[158] Shorter duration of breastfeeding is associated with pneumonia mortality, particularly among infants <5 months of age.[159] Mortality among infants who are not breastfed compared with exclusively breastfed infants through 5 months of age is ~15-fold higher.[159] Breastfeeding should be encouraged for the first 6 months of a child's life, irrespective of maternal HIV or ART use,[160] and may be considered for the first 2 years in CLWH.[161]

#### Micronutrient supplementation

Specific micronutrients that may play a role in the prevention of pneumonia are discussed below.

##### Vitamin A

Vitamin A supplementation reduces severity of respiratory complications of measles.[109] However, a meta-analysis of the impact of vitamin A supplementation on all-cause pneumonia morbidity and mortality showed no consistent effect on pneumonia-specific mortality.[110] Provision of vitamin A supplementation in children with vitamin A deficiency has been associated with improved outcomes.

**Table 8. Summary: Measures to prevent pneumonia in children**

| General preventive strategies | Summary |
|---|---|
| Nutrition | Malnourished children are at increased risk for severe pneumonia and mortality<br>Breastfeeding is protective |
| Micronutrient supplementation | |
| Vitamin A | Vitamin A should be dosed according to the Road to Health card schedule<br>100 000 IU stat at 6 months; 200 000 IU stat at 12 months; 200 000 IU stat at 18 months<br>From 24 months onwards, 200 000 IU every 6 months from 2 to 5 years of age |
| Vitamin D | Vitamin D-deficient children are at increased risk for CAP, supplement with vitamin D 400 IU daily |
| Zinc | Zinc 10 mg (for infants) and 20 mg (for older children) daily significantly reduces the risk of pneumonia |
| Reduction in passive smoking and indoor fuel exposure | Environmental exposure to cigarette smoke or indoor air pollution is strongly correlated with impaired lung health in children |
| Infection prevention and control and physical distancing | Careful attention to limiting transmission of respiratory pathogens reduces the burden of respiratory illness<br>Hand hygiene<br>Cough etiquette<br>Decontamination of environmental surfaces<br>Use of masks |
| **Specific preventive strategies** | |
| Immunisation | Administered at |
| BCG | Birth |
| Pneumococcal conjugate vaccine | 6 weeks, 14 weeks and 9 months |
| Hib conjugate vaccine and pertussis vaccine | 6 weeks, 10 weeks, 14 weeks and 18 months as part of the hexavalent vaccine |
| Influenza vaccine | Not routinely administered in the SA EPI, but should be considered annually for children ≥6 months of age at risk for severe influenza, including those with congenital cardiac disease, chronic lung disease, immunosuppression and neuromuscular disease |
| Measles-containing vaccine | 6 months and 12 months |
| Combination ART | Expeditious initiation of ART at the earliest opportunity must be implemented routinely for all CLWH to restore immunological function and prevent infectious complications of HIV; ideally, the diagnosis should be made at birth and ART initiated within the first week of life, as per National ART guidelines |
| Prophylaxis | |
| Prevention of PCP | Co-trimoxazole prophylaxis is crucial in the prevention of PCP and all-cause mortality in CLWH and those with immunosuppression from other causes; refer to the SA paediatric ART guidelines for details and Table 9 |
| Prevention of tuberculosis | INH is an under-utilised preventive strategy in SA children exposed to a household contact with tuberculosis – INH 10 mg/kg × 6 months is recommended<br>CLWH or other underlying immunosuppression with a positive tuberculin skin test, even in the absence of a contact, should be given INH × 6 months. This may also be considered for children newly diagnosed with HIV. The maximum daily dose of INH is 300 mg |
| Prevention of CMV | Although CMV pneumonia is considered to be an important infection in HIV-infected infants, no recommendation on chemoprophylaxis has been adopted |
| Prevention of RSV | The cost of monoclonal antibody prophylaxis (palivizumab) against RSV is very high – therefore, widespread use is not feasible at a programmatic level; ex-premature infants <6 months of age, and those with congenital cardiac disease or chronic lung disease <1 year of age during the course of the RSV season, benefit most from this preventive measure given monthly through the season |

CAP = community-acquired pneumonia; BCG = bacillus Calmette-Guérin; Hib = *Haemophilus influenzae* type b; SA = South Africa; EPI = Expanded Programme on Immunisation; ART = antiretroviral therapy; CLWH = children living with HIV; PCP = *Pneumocystis jirovecii* pneumonia; IPT = isoniazid-preventive therapy; INH = isoniazid; CMV = cytomegalovirus; RSV = respiratory syncytial virus.

Vitamin A supplementation should be administered as per national guidelines: 100 000IU at 6 months, 200 000IU at 12 and 18 months, and 200 000IU every 6 months from 2 to 5 years of age.[162]

**Vitamin D**

While empiric therapy using vitamin D in hospitalised children with CAP is not beneficial, observational studies have identified an increased risk of pneumonia in children <5 years old with subclinical vitamin D deficiency (evidence level III).[163] A meta-analysis of the role of vitamin D supplementation in pneumonia prevention found a significant protective effect (evidence level Ia).[164] However, in a clinical trial conducted in Asian children <5 years of age, oral doses of vitamin D had no protective effect on the incidence of the first episode of pneumonia (evidence level Ib).[165] Currently, there is lack of consensus as to what serum levels of vitamin D are protective against LRTI, and also little evidence as to the best supplemental regimen (evidence level Iva).[113] British guidelines recommend a daily intake of 400 IU vitamin D, regardless of age group (evidence level Ia).[166]

##### Vitamin E

There is very little evidence to support vitamin E supplementation for the prevention of pneumonia in children (evidence level IVa).[167,168]

##### Zinc

Daily prophylactic elemental zinc, 10 mg (infants) and 20 mg (older children), may substantially reduce the incidence of pneumonia, particularly in malnourished children.[169] A pooled analysis of randomised controlled trials of zinc supplementation in well-nourished and malnourished children found that children who received zinc supplementation had a significant reduction in pneumonia incidence compared with those who received placebo (odds ratio (OR) 0.59; CI 0.41 - 0.83) (evidence level Ia).[170]

#### Reduction in tobacco smoke or indoor fuel exposure

Active and passive exposure to tobacco should be strongly discouraged in women of child-bearing age, particularly among pregnant women,[171] and more generally in the household.[172]

Exposure to fumes from indoor cooking fuels should be limited by opening windows and doors when cooking; the chimney should function well; the stove should be cleaned and maintained; and there should be safer child location practices while fires are burning in the house.[173] The practice of carrying children on caregivers' backs while cooking is as an independent risk factor for LRTI morbidity and mortality.[174] Children should sleep in rooms separate from where food is cooked or where open fires or paraffin burners are used (evidence level Ib).

#### Infection prevention, control, use of masks and physical distancing

Hand hygiene and respiratory etiquette are crucial in limiting transmission of respiratory pathogens.[175,176] Reinforcement of hand hygiene decreases the prevalence of respiratory tract illness in adults by 14% (95% CI 11 - 17) in non-pandemic influenza seasons.[177] A systematic review and meta-analysis of the effect of hand hygiene in limiting illness in children suggested that in primary and secondary schools, hand hygiene may decrease the incidence of respiratory tract infections among learners (evidence level Ia).[178] Although young children are not generally able to adhere to respiratory etiquette practices,[179] older children, caregivers and health workers should adopt these practices to limit the transmission of respiratory pathogens.[175]

Universal use of cloth face masks by children and adults in public is an effective public health intervention to reduce transmission of respiratory viruses, including SARS-CoV-2, in addition to other public health measures.[180] In health facilities, all healthcare workers should wear a surgical mask in addition to practising hand hygiene, physical distancing and environmental decontamination to prevent SARS-CoV-2 transmission. N95 or respiratory masks should be used when taking care of children with confirmed or suspected COVID-19, or when doing aerosolising procedures.[180, 181]

### Specific preventive strategies

#### Immunisation

##### Routine immunisations

All children should receive routine vaccines, including bacillus Calmette-Guérin (BCG), measles, diphtheria-pertussis-tetanus (DPT) toxoid, Hib, polysaccharide-protein conjugate vaccine (HibCV) and PCV as per the SA immunisation schedule.[182] The nature and degree of immunosuppression in CLWH may impact on the efficacy and duration of vaccine-induced protection.[183-187] CLWH treated with ART from early infancy, and responding well to such therapy, demonstrate similar immunogenicity and anamnestic immune responses to most childhood vaccines compared with HIV-unexposed infants.[188,189] HEU infants may have lower concentrations of transplacental acquired antibodies for some vaccine-preventable diseases,[190,191] which could increase their susceptibility to pneumonia during early infancy. The immune responses to all vaccines are, however, similar or more immunogenic for HEU than for HIV-unexposed infants,[188,189] and there is similar persistence of protective antibody concentrations and memory responses.[192]

##### Specific vaccines

**BCG vaccine.** *M. tuberculosis* may be a direct pathogen in pneumonia or may predispose to bacterial infection (including from pneumococcus).[70,193] A birth dose of BCG vaccine is effective in preventing disseminated tuberculosis in young children, but has variable effectiveness (average 50%; range 0 - 84% effectiveness) in prevention of pulmonary tuberculosis, with lower effectiveness in studies on children from tropical countries.[194,195] A birth dose has also been shown to have nonspecific benefits in improving overall child survival in some settings.[196]]

**Pneumococcal vaccine.** Multiple post-licensure effectiveness studies (using 10- and 13-valent PCV) in a diversity of settings have demonstrated a 17% (95% CI 11 - 22) and 31% (95% CI 26 - 35 ) reduction in hospitalisation rates for clinically and radiologically confirmed pneumonia, respectively.[197] In children aged 24 - 59 months a meta-analysis found a reduction of 9% (95% CI 5 - 14) and 24% (95% CI 12 - 33) in hospitalisation rates for clinically and radiologically confirmed pneumonia, respectively (evidence level Ia).[197]

In SA, PCV (currently 13-valent) is administered at 6 and 14 weeks of age, followed by a booster dose at 9 months of age (evidence level Ib). This schedule has been shown to be effective in reducing all-cause pneumonia hospitalisation by 33% and 39% in CLWH and HIV-uninfected children, respectively.[5]

The 23-valent pneumococcal polysaccharide vaccine (Pneumovax 23) is recommended for children >2 years old, who are at risk of developing invasive pneumococcal disease, including those with sickle cell disease, chronic pulmonary disease and cardiovascular disease,[198,199] and is included in the SA Essential Drugs List for

Paediatrics for administration to such patients. It should be preceded by a single dose of PCV, given at least 1 month before (evidence level III).[200,201]

The need for further booster doses of PCV in older CLWH remains to be determined, but the indirect effect of childhood PCV immunisation in reducing transmission and circulation of vaccine-serotype pneumococci could mitigate waning of immunity in CLWH and other high-risk groups that remain susceptible to developing severe pneumonia in later childhood.[202] The WHO currently recommends that a booster dose of PCV may be considered in the second year of life in CLWH (evidence level II).[203]

**Hib conjugate vaccine**. Vaccination with HibCV, as part of a combination vaccine, is recommended as a 3-dose primary series, and includes a booster dose at 15 - 18 months of age in SA. HibCV is less effective in CLWH not on ART;[204] however, the immunogenicity of the vaccine and persistence of memory responses in the second year of life are similar in CLWH vaccinated when already initiated on ART at the time of immunisation.[205]

**Pertussis vaccine.** Pertussis remains one of the most poorly controlled vaccine-preventable diseases globally and causes severe disease in young infants (especially in those <3 months of age) and incompletely immunised children (evidence level Ib).[206,207]

Pertussis vaccine formulations include whole-cell containing (wP) and *B. pertussis* protein-only component acellular vaccines (aP). Whole-cell pertussis vaccines, but not aP protein-containing vaccines, induce mucosal immunity and protect against *B. pertussis* mucosal infection and transmission.[208] Furthermore, the duration of protection of wP is 8 - 12 years compared with 4 - 5 years for aP vaccines.[209] Currently, only aP-containing combination vaccines are available in SA.

Pertussis outbreaks have been temporally associated with transitioning from wP to aP formulations in many high-resource settings, attributed to the waning of immunity in the absence of repeat booster doses at school entry and beyond.[209] Children primed with aP-containing vaccines should receive booster doses of aP vaccines (dTaP) at school entry and possibly every 10 years thereafter (not yet part of the Expanded Programme on Immunisation (EPI)) (evidence level II).[210]

Prevention of pertussis in very young infants, who are at greatest risk of severe disease, is not achievable through infant immunisation, and transitioning from wP to aP could increase the burden of pertussis in in this group.[53] Acellular pertussis vaccination of pregnant women is 90% effective in reducing pertussis in infants <3 months of age (evidence level Ia).[211]

**Influenza vaccine.** Only the sub-unit inactivated influenza vaccine is available in SA for annual administration. There are limited data on its efficacy in children, ranging from 33% to 73%, depending on vaccine preparation and influenza subtype targeted.[212 Current evidence suggests that influenza vaccination is safe in CLWH; however, a randomised controlled trial failed to demonstrate vaccine efficacy.[213] Nonetheless, there remains a recommendation that CLWH should be offered influenza vaccination before the start of winter, particularly if they have underlying chronic lung disease (evidence level III).

Children >6 months of age with underlying medical conditions are considered to be a high risk for complications of influenza, and are prioritised for annual vaccination. Such children comprise those with chronic pulmonary disease (including asthma), cardiac disease, chronic renal or hepatic diseases, diabetes mellitus, metabolic disorders, sickle cell anaemia and other haemoglobinopathies, morbid obesity, immunosuppression, cerebral palsy or other neuromuscular conditions.[214] Family members and siblings of such patients should also be vaccinated.[214] Two doses of inactivated influenza vaccine, administered 1 month apart, are recommended for children 6 months - 9 years of age who have never been vaccinated; and a single dose if immunised in previous seasons.[214]

Recent randomised controlled trials have demonstrated that influenza vaccination of pregnant women was 50% efficacious in reducing PCR-confirmed influenza illness in their infants until 24 weeks of age. In SA and Mali, vaccination of pregnant women was more effective in preventing influenza illness in infants during the first 3 months of life (vaccine efficacy ‑ 85%) with subsequent waning and a non-significant reduction between 3 and 6 months of age.[215] Maternal influenza vaccination also reduced all-cause clinically diagnosed severe pneumonia or pneumonia hospitalisation by 30% in infants during the first 6 months.[215,216]

Influenza immunisation should ideally be administered prior to the onset of the influenza season (which typically occurs from May to September in SA),[73,217] but can also be given during the influenza season. Due to the potential of year-on-year genetic drift of seasonal influenza virus strains, current vaccine formulations are updated annually, and a repeat vaccination is required each year.

**Measles vaccine.** Measles remains a public health concern, and failure to achieve and sustain high immunisation coverage rates (>95%) against this highly contagious virus results in ongoing outbreaks in a diversity of settings, including SA.[217,218] Recent changes in the epidemiology of measles include a greater susceptibility of disease in very young infants (as early as 4 months of age).[219] This is due to lower antibody concentrations in pregnant women who have acquired immunity through vaccination, rather than through wild-type virus exposure, as well as possible waning of immunity in women living with HIV.[220,221]

The WHO recommends that children receive 2 doses of the measles vaccine, the first at 9 months of age and a booster dose at 15 - 18 months of age.[222] However, for infants born to women living with HIV, and in settings with a high risk of measles in young infants, an additional dose is recommended at 6 months of age.[222] In SA, 2-dose measles vaccination is administered at 6 and 12 months of age. This induces seroprotective titres in ~55% of infants following the first dose of vaccine, and in >98% in HIV-exposed and HIV-unexposed children after the second dose of vaccine.[223]

### Combination antiretroviral therapy

The use of ART to reconstitute immunity is very effective for decreasing the incidence of pneumonia and opportunistic infections in CLWH. Combination ART should be initiated on diagnosis of HIV in all children, irrespective of clinical or immunological staging. Screening for HIV infection in newborns of HIV-infected women by means of PCR testing at birth and repeated PCR testing during the infant and breastfeeding period is standard of care in SA, with initiation of ART as soon as possible after confirmation of HIV infection, and continued lifelong thereafter.

### Prophylaxis

#### Prevention of *Pneumocystis jirovecii* pneumonia

Updated recommendations for the management of CLWH were published in 2019 (SA ART guidelines: https://sahivsoc.org/Files/2019%20Abridged%20ART%20Guidelines%2010%20October%202019.pdf) (Table 9).[224]

Although the WHO recommends PCP prophylaxis for HEU infants from 4 to 6 weeks of age until HIV infection has been excluded after complete cessation of breastfeeding, two southern African randomised controlled trials have shown that co-trimoxazole confers no survival advantage over placebo in this subset of children; therefore, this is not recommended in SA.[225,226]

#### Prevention of tuberculosis

All children <5 years of age exposed to a household tuberculosis contact or other close tuberculosis contact should be given isoniazid preventive therapy (IPT) (10 mg/kg; maximum dose 300 mg) daily for 6 months once tuberculosis disease has been excluded. CLWH exposed to a household contact should be given prophylaxis for 6 months, irrespective of their age. A 6-month course of IPT should also be given to tuberculin skin test (TST)-positive HIV-infected children, even in the absence of a known household contact.[227] There are conflicting data on the use of primary IPT in CLWH in the absence of a tuberculosis contact.[228,229] Newly HIV-diagnosed and clinically symptomatic CLWH may benefit from a 6-month course of IPT, irrespective of TST results.

Short-course preventive therapy using rifampicin and isoniazid must not be used in the context of tuberculosis prevention in HIV-exposed neonates born to mothers with active tuberculosis, as the rifampicin component interferes with the prevention of mother-to-child transmission (ART) regimens.[230]

Current WHO guidelines encourage use of preventive therapy with multidrug-resistant tuberculosis (MDR-TB) based on individualised risk assessment for children exposed to source cases. In children exposed to a source case with ofloxacin-susceptible *M. tuberculosis*, a 6-month course of ofloxacin, ethambutol and high-dose isoniazid has been found to be well tolerated.[231]

**Prevention of cytomegalovirus disease in HIV-infected children**

There is no evidence to support a specific intervention in the prevention of CMV disease in CLWH.[232]

**Prevention of respiratory syncytial virus**

Although the humanised monoclonal-specific antibody for the prevention of RSV infections (palivizumab) is available, it is very expensive. Children most likely to benefit are those at risk of severe RSV infection, i.e. babies born prematurely who are <6 months of chronological age at the onset of the RSV season, or children with chronic lung disease or congenital cardiac disease who are <1 year of age at the onset of the RSV season.[233] A meta-analysis on the effectiveness of palivizumab against RSV hospitalisation reported 71% (95% CI 46 - 84) effectiveness in infants born at <35 weeks' gestational age, and ~45% in those with chronic lung disease or congenital heart disease (evidence level Ia).[234] Palivizumab should be given monthly for the duration of the RSV season (from February to July) in most of SA.[235,236]

Other strategies for prevention of severe RSV disease in infants, including antenatal vaccination of expectant mothers and long-acting monoclonal antibody preparations, are currently under investigation.

**Table 9. Indications for co-trimoxazole prophylaxis in children living with HIV**

| |
|---|
| **Indications to start prophylaxis** |
| Children 6 weeks - 1 year of age, irrespective of clinical stage or immunological status |
| Children 1 - 5 years of age with<br>CD4+ counts ≤25% or WHO stage 2 or greater |
| Children ≤5 years of age with<br>Prior PCP |
| Children >5 years of age with<br>CD4+ counts ≤200 cells/µL or WHO stage 2 or greater |
| **Indications to discontinue prophylaxis** |
| Children 1 - 5 years of age with<br>CD4+ counts >25%, regardless of clinical stage |
| Children >5 years of age with<br>CD4+ counts >200 cells/µL, regardless of clinical stage |

WHO = World Health Organization; PCP = *Pneumocystis jirovecii* pneumonia.

### Summary – prevention

1. Exclusive breastfeeding is recommended for all infants, irresepctive of HIV exposure status, for the first six months of life.
2. All children should receive BCG vaccine at birth.
3. All children should receive two doses of a primary series of 13-valent PCV during early infancy, at least two months apart (6 and 14 weeks of age), and a booster dose at 9 months of age (evidence level Ia).
4. 23-valent pneumococcal polysaccharide vaccine (Pneumovax 23) is recommended for children >2 years at risk of developing invasive pneumococcal disease, including children with chronic pulmonary or cardiovascular disease (evidence level III).
5. All children should receive at least three doses of HibCV. In SA, HibCV vaccination is recommended (as part of a combination vaccine) as 6, 10 and 14 weeks of age, and a booster dose at 15 - 18 months of age (evidence level Ib).[205]
6. All children should receive a three dose primary infant series of aP or wP containing vaccine at 6, 10 and 14 weeks of age; and a booster dose of vaccine at 15 - 18 months of age. In SA, only aP-containing vaccine preparations are currently available.
7. Influenza vaccine is recommended annually for children at high risk of severe influenza disease. The recommended schedule of sub-unit inactivated influenza vaccine who have not been vaccinated is two doses, spaced 1 month apart. Children who are >9 years or those who have been immunised previously require only a single dose of vaccine. The vaccine should be administered before the start of the influenza season.
8. Pregnant women should receive inactivated influenza vaccine at any stage of pregnancy, and should be prioritised for vaccination.[237]
9. All children should receive measles vaccination at 6 and 12 months of age.[222]
10. ART is life-saving in CLWH, who should be expedited onto treatment as soon as the diagnosis is confirmed (evidence level Ia).
11. Co-trimoxazole preventive therapy is crucial for prevention of PCP in young CLWH, and in older, ART-naïve or severely immunosuppressed children (Evidence level Ia).

12. IPT is under-utilised in SA, but should be used in CLWH of any age or an immunocompetent child under 5 years, with a household contact with tuberculosis (evidence level II). IPT is also indicated for any CLWH who is TST positive.

### Special circumstances: HIV infection or exposure

HIV infection rates in children have declined significantly with the advent of effective PMTCT interventions, from 30% to 1 - 3% in SA.[238] There is subsequently a large population of children born to HIV-infected mothers who are HEU.[238] HEU infants have an increased risk of pneumonia, particularly in the first 6 months of life (evidence level Ib).[10] The exact mechanisms of vulnerability in HEU infants may involve *in utero* exposure to HIV viral proteins, exposure to ART, lack of effective protective maternal antibodies, or abnormalities in immunological responses (evidence level III).[238,239]

Prior to the ART roll-out, CLWH had a 4 - 6-fold increased risk of developing severe pneumonia,[14,240] and a 4 - 6-fold increased risk of death once hospitalised for pneumonia (evidence level II).[9,240] However, widespread use of ART has markedly reduced this burden, with a decline in mortality rates among children on ART from 7.1 per 100 person-years to 0.6 per 100 person years in a multicentre prospective cohort study from the USA.[241]

HIV-infected infants not on ART, especially those with viral load ≥100 000 copies/mL and CD4 <15%, are at greatest risk of developing severe disease (evidence level Ib).[242] Additional risk factors for severe disease include poor nutritional status and anaemia (evidence level Ib).[240]

### Aetiology of pneumonia in HIV-infected children

In the era of ART, the aetiology of pneumonia is similar amongst CLWH, HEU and HIV-unexposed children (Supplementary Tables 1 and 2) [243] However, in HIV-infected infants, *P. jirovecii* is still the leading cause of hospitalisation.[62]

*M. tuberculosis* should be considered in HIV-infected children with pneumonia, as evidence suggests an increased risk for tuberculosis (evidence level Ib).[240] HIV-infected infants not on ART have a 20-fold higher risk of developing culture-confirmed tuberculosis compared with HIV-uninfected infants (evidence level 1b).[244] This risk declines with the introduction of ART.[245]

### Treatment

Empiric CAP treatment of HEU infants and HIV-infected children is the same as for HIV-unexposed children. There are currently no studies comparing regimens for and outcomes of HIV-infected and HEU infants.[246] HIV infection should be considered in all children with CAP, and appropriate testing must be provided (evidence level IVa).

#### Pneumocystis pneumonia

Few children currently acquire vertically transmitted HIV infection – most of these are diagnosed through early infant testing and receive ART and co-trimoxazole prophylaxis.[247] Women who tested HIV-negative during pregnancy may acquire HIV infection late in gestation or while breastfeeding – their infants are at risk of PCP (evidence level Ib).[248-251] Some children with primary immunodeficiencies or severe malnutrition are also at risk of PCP (evidence level IIb).[252] Empiric therapy for PCP should not be withheld pending results of confirmatory laboratory testing.

Co-trimoxazole administered intravenously or orally, 5 mg trimethroprim/25 mg sulfamethoxazole/kg/dose, 6-hourly for 21 days, reduces mortality from PCP in infants.[253] Following treatment, daily co-trimoxazole prophylaxis needs to be continued until CD4+ counts recover as per SA ART guidelines (Table 9).[224] There is conflicting evidence regarding the effect of adjunctive corticosteroids.[254] However, we recommend a short course of corticosteroids for children with PCP, initiated within 72 hours of diagnosis (prednisone 1 - 2 mg/kg orally daily for 7 days, tapered over the next 7 days) (evidence level IVa).[255]

#### Cytomegalovirus

CMV pneumonia should be considered in HIV-infected or HEU infants <6 months of age with severe hypoxaemia or requiring mechanical ventilation.[94,256] The optimal treatment remains unclear. Some clinicians initiate treatment if the whole-blood CMV viral load is >4.1 $\log_{10}$ copies/mL,[257] but this may delay therapy. Alternatively, initiate empiric ganciclovir and discontinue if CMV viral load results indicate low-level or absent viraemia (evidence level IVa).

In children with high-level CMV DNAemia, clinical improvement and/or a decline in viral load should prompt switching from intravenous ganciclovir to oral valganciclovir (evidence level IVa). Ganciclovir should be given at 5 mg/kg intravenously 12-hourly until oral therapy is tolerated, then switch to valganciclovir 16 mg/kg orally 12-hourly until completion of 21 days of treatment. Thereafter, administer valganciclovir 16 mg/kg orally daily to complete a total of 42 days of therapy.[106]

#### Atypical organisms

If 'atypical' organisms are considered in the differential diagnosis, a macrolide should be added (see above: Treatment of childhood pneumonia: Which empiric antibiotic?) (evidence level IVa).

### Summary – HIV infection or exposure

1. A broader range of pathogens is responsible for pneumonia in CLWH, encompassing opportunistic infections such as PCP and CMV, *S. aureus* and gram negative organisms (evidence level Ib).
2. *M. tuberculosis* should be considered in CLWH who have an increased risk for tuberculosis (evidence level Ib).
3. Empiric antibiotic treatment is the same in CLWH, HEU and HIV unexposed children, although treatment for PCP (evidence level Ib) and/or CMV pneumonia (evidence level IVa) should be considered in HIV-infected infants with severe hypoxaemia.

1. Sly PD, Zar HJ. The spectrum of lower respiratory tract illness in children after pneumococcal conjugate vaccination. Am J Resp Crit Care Med 2017;195(1):13-15. https://doi.org/10.1164/rccm.201607-1461ED
2. Roth GA, Abate D, Abate KH, et al. Global, regional, and national age-sex-specific mortality for 282 causes of death in 195 countries and territories, 1980–2017: A systematic analysis for the Global Burden of Disease Study 2017. Lancet 2018;392(10159):1736-1788. https://doi.org/10.1016/S0140-6736(18)32203-7
3. McAllister DA, Liu L, Shi T, et al. Global, regional, and national estimates of pneumonia morbidity and mortality in children younger than 5 years between 2000 and 2015: A systematic analysis. Lancet Glob Health 2019;7(1):e47-e57. https://doi.org/10.1016/S2214-109X(18)30408-X

4. Goga A, Slogrove A, Wedderburn CJ, et al. The impact of health programmes to prevent vertical transmission of HIV. Advances, emerging health challenges and research priorities for children exposed to or living with HIV: Perspectives from South Africa. S Afr Med J 2019;109(11b):77-82. https://doi.org/10.7196/SAMJ.2019.v109i11b.14292
5. Izu A, Solomon F, Nzenze SA, et al. Pneumococcal conjugate vaccines and hospitalization of children for pneumonia: A time-series analysis, South Africa, 2006-2014. Bull World Health Org 2017;95(9):618-628. https://doi.org/10.2471/BLT.16.187849
6. von Gottberg A, de Gouveia L, Tempia S, et al. Effects of vaccination on invasive pneumococcal disease in South Africa. New Engl J Med 2014;371(20):1889-1899. https://doi.org/10.1056/NEJMoa1401914
7. le Roux DM, Nicol MP, Myer L, et al. Lower respiratory tract infections in children in a well-vaccinated South African birth cohort: Spectrum of disease and risk factors. Clin Infect Dis 2019;69(9):1588-1596. https://doi.org/10.1093/cid/ciz017
8. Hutton HK, Zar HJ, Argent AC. Clinical features and outcome of children with severe lower respiratory tract infection admitted to a pediatric intensive care unit in South Africa. J Trop Pediatr 2018;65(1):46-54. https://doi.org/10.1093/tropej/fmy010
9. Cohen C, Walaza S, Moyes J, et al. Epidemiology of viral-associated acute lower respiratory tract infection among children <5 years of age in a high HIV prevalence setting, South Africa, 2009-2012. Pediatr Infect Dis J 2015;34(1):66-72. https://doi.org/10.1097/INF.0000000000000478
10. Cohen C, Moyes J, Tempia S, et al. Epidemiology of acute lower respiratory tract infection in HIV-exposed uninfected infants. Pediatrics 2016;137(4):e20153272. https://doi.org/10.1542/peds.2015-3272
11. McMorrow ML, Tempia S, Walaza S, et al. The role of human immunodeficiency virus in influenza-and respiratory syncytial virus-associated hospitalizations in South African children, 2011–2016. Clin Infect Dis 2018;68(5):773-780. https://doi.org/10.1093/cid/ciy532
12. le Roux SM, Abrams EJ, Donald KA, et al. Infectious morbidity of breastfed, HIV-exposed uninfected infants under conditions of universal antiretroviral therapy in South Africa: A prospective cohort study. Lancet Child Adolesc Health 2020;4(3):220-231. https://doi.org/10.1016/S2352-4642(19)30375-X
13. von Mollendorf C, von Gottberg A, Tempia S, et al. Increased risk for and mortality from invasive pneumococcal disease in HIV-exposed but uninfected infants aged <1 year in South Africa, 2009-2013. Clin Infect Dis 2015;60(9):1346-1356. https://doi.org/10.1093/cid/civ059
14. Jackson S, Mathews KH, Pulanic D, et al. Risk factors for severe acute lower respiratory infections in children: A systematic review and meta-analysis. Croat Med J 2013;54(2):110-121. https://doi.org/10.3325/cmj.2013.54.110
15. Sonego M, Pellegrin MC, Becker G, Lazzerini M. Risk factors for mortality from acute lower respiratory infections (ALRI) in children under five years of age in low and middle-income countries: A systematic review and meta-analysis of observational studies. PloS One 2015;10(1):e0116380. https://doi.org/10.1371/journal.pone.0116380
16. McCollum ED, Park DE, Watson NL, et al. Listening panel agreement and characteristics of lung sounds digitally recorded from children aged 1 - 59 months enrolled in the Pneumonia Etiology Research for Child Health (PERCH) case-control study. BMJ Open Respir Res 2017;4(1):e000193.https://doi.org/10.1136/bmjresp-2017-000193
17. World Health Organization. Revised WHO Classification and Treatment of Pneumonia in Children at Health Facilities: Evidence Summaries. Geneva: WHO, 2014.
18. Zar HJ. Hypoxia and signs of increased work of breathing are most strongly associated with radiographic pneumonia in children. BMJ Evidence-based Med 2018;23(2):77-78. https://doi.org/10.1136/bmjebm-2017-110874
19. Dean P, Florin TA. Factors associated with pneumonia severity in children: A systematic review. J Pediatr Infect Dis Soc 2018;7(4):323-334. https://doi.org/10.1093/jpids/piy046
20. Harris M, Clark J, Coote N, et al. British Thoracic Society guidelines for the management of community acquired pneumonia in children: Update 2011. Thorax 2011;66(Suppl 2):ii1-ii23. https://doi.org/10.1136/thoraxjnl-2011-200598
21. McCollum ED, King C, Deula R, et al. Pulse oximetry for children with pneumonia treated as outpatients in rural Malawi. Bull World Health Organ 2016;94(12):893. https://doi.org/10.2471/BLT.16.173401
22. Bradley JS, Byington CL, Shah SS, et al. The management of community-acquired pneumonia in infants and children older than 3 months of age: Clinical practice guidelines by the Pediatric Infectious Diseases Society and the Infectious Diseases Society of America. Clin Infect Dis 2011;53(7):25-76. https://doi.org/10.1093/cid/cir531
23. Andronikou S, Lambert E, Halton J, et al. Guidelines for the use of chest radiographs in community-acquired pneumonia in children and adolescents. Pediatr Radiol 2017;47(11):1405-1411. https://doi.org/10.1007/s00247-017-3944-4
24. Lynch T, Gouin S, Larson C, Patenaude Y. Should the lateral chest radiograph be routine in the diagnosis of pneumonia in children? A review of the literature. Paediatr Child Health 2003;8(9):566-568. https://doi.org/10.1093/pch/8.9.566
25. O'Grady KF, Torzillo PJ, Frawley K, Chang AB. The radiological diagnosis of pneumonia in children. Pneumonia (Nathan) 2014;5(Suppl 1):38-51. https://doi.org/10.15172/pneu.2014.5/482
26. García-Basteiro AL, López-Varela E, Augusto OJ, et al. Radiological findings in young children investigated for tuberculosis in Mozambique. PLoS ONE 2015;10(5):e0127323. https://doi.org/10.1371/journal.pone.0127323
27. Zar HJ, Andronikou S, Nicol MP. Advances in the diagnosis of pneumonia in children. BMJ 2017;358:j2739. https://doi.org/10.1136/bmj.j2739
28. Lissaman C, Kanjanauptom P, Ong C, Tessaro M, Long E, O'Brien A. Prospective observational study of point-of-care ultrasound for diagnosing pneumonia. Arch Dis Childh 2019;104(1):12-18. https://doi.org/10.1136/archdischild-2017-314496
29. Suren P, Try K, Eriksson J, Khoshnewiszadeh B, Wathne KO. Radiographic follow-up of community-acquired pneumonia in children. Acta Paediatr 2008;97(1):46-50. https://doi.org/10.1111/j.1651-2227.2007.00567.x
30. Iorio G, Capasso M, Prisco S, et al. Lung ultrasound findings undetectable by chest radiography in children with community-acquired pneumonia. Ultrasound Med Biol 2018;44(8):1687-1693. https://doi.org/10.1016/j.ultrasmedbio.2018.04.007
31. Pereda MA, Chavez MA, Hooper-Miele CC, et al. Lung ultrasound for the diagnosis of pneumonia in children: A meta-analysis. Pediatrics 2015;135(4):714-722. https://doi.org/10.1542/peds.2014-2833
32. Korppi M, Don M, Valent F, Canciani M. The value of clinical features in differentiating between viral, pneumococcal and atypical bacterial pneumonia in children. Acta Paediatr 2008;97(7):943-947. https://doi.org/10.1111/j.1651-2227.2008.00789.x
33. Ruuskanen O, Lahti E, Jennings LC, Murdoch DR. Viral pneumonia. Lancet 2011;377(9773):1264-1275. https://doi.org/10.1016/S0140-6736(10)61459-6
34. Scott JA, Wonodi C, Moisi JC, et al. The definition of pneumonia, the assessment of severity, and clinical standardization in the Pneumonia Etiology Research for Child Health study. Clin Infect Dis 2012;54(Suppl 2):S109-S116. https://doi.org/10.1093/cid/cir1065
35. Shi T, McAllister DA, O'Brien KL, et al. Global, regional, and national disease burden estimates of acute lower respiratory infections due to respiratory syncytial virus in young children in 2015: A systematic review and modelling study. Lancet 2017;390(10098):946-958. https://doi.org/10.1016/S0140-6736(17)30938-8
36. Mathew JL. Etiology of childhood pneumonia: What we know, and what we need to know!: Based on 5th Dr I C Verma Excellence Oration Award. Ind J Pediatr 2018;85(1):25-34. https://doi.org/10.1007/s12098-017-2486-y
37. Murdoch DR, O'Brien KL, Driscoll AJ, et al. Laboratory methods for determining pneumonia etiology in children. Clin Infect Dis 2012;54(Suppl 2):S146-S152. https://doi.org/10.1093/cid/cir1073
38. Principi N, Esposito S. Biomarkers in pediatric community-acquired pneumonia. Int J Mol Sci 2017;18(2):447. https://doi.org/10.3390/ijms18020447
39. Higdon MM, Le T, O'Brien KL, et al. Association of C-reactive protein with bacterial and respiratory syncytial virus-associated pneumonia among children aged <5 years in the PERCH study. Clin Infect Dis 2017;64(Suppl 3):S378-S386. https://doi.org/10.1093/cid/cix150
40. Hammitt LL, Murdoch DR, Scott JAG, et al. Specimen collection for the diagnosis of pediatric pneumonia. Clin Infect Dis 2012;54(Suppl 2):S132-S139. https://doi.org/10.1093/cid/cir1068
41. Driscoll AJ, Deloria Knoll M, Hammitt LL, et al. The effect of antibiotic exposure and specimen volume on the detection of bacterial pathogens in children with pneumonia. Clin Infect Dis 2017;64(Suppl 3):S368-S377. https://doi.org/10.1093/cid/cix101
42. Brent AJ, Ahmed I, Ndiritu M, et al. Incidence of clinically significant bacteraemia in children who present to hospital in Kenya: Community-based observational study. Lancet 2006;367(9509):482-488. https://doi.org/10.1016/S0140-6736(06)68180-4
43. Iroh Tam PY, Bernstein E, Ma X, Ferrieri P. Blood culture in evaluation of pediatric community-acquired pneumonia: A systematic review and meta-analysis. Hosp Pediatr 2015;5(6):324-336. https://doi.org/10.1542/hpeds.2014-0138
44. Madhi SA, Petersen K, Madhi A, Khoosal M, Klugman KP. Increased disease burden and antibiotic resistance of bacteria causing severe community-acquired lower respiratory tract infections in human immunodeficiency virus type 1-infected children. Clin Infect Dis 2000;31(1):170-176. https://doi.org/10.1086/313925
45. Zar HJ, Tannenbaum E, Hanslo D, Hussey G. Sputum induction as a diagnostic tool for community-acquired pneumonia in infants and young children from a high HIV prevalence area. Pediatr Pulmonol 2003;36(1):58-62. https://doi.org/10.1002/ppul.10302
46. Zar HJ, Hanslo D, Apolles P, Swingler G, Hussey G. Induced sputum versus gastric lavage for microbiological confirmation of pulmonary tuberculosis in infants and young children: A prospective study. Lancet 2005;365(9454):130-134. https://doi.org/10.1016/S0140-6736(05)17702-2
47. Muloiwa R, Dube FS, Nicol MP, Zar HJ, Hussey GD. Incidence and diagnosis of pertussis in South African children hospitalized with lower respiratory tract infection. Pediatr Infect Dis J 2016;35(6):611-616. https://doi.org/10.1097/INF.0000000000001132

48. Zar HJ, Barnett W, Stadler A, Gardner-Lubbe S, Myer L, Nicol MP. Aetiology of childhood pneumonia in a well vaccinated South African birth cohort: A nested case-control study of the Drakenstein Child Health Study. Lancet Respir Med 2016;4(6):463-472. https://doi.org/10.1016/S2213-2600(16)00096-5
49. Pneumonia Etiology Research for Child Health Study Group. Causes of severe pneumonia requiring hospital admission in children without HIV infection from Africa and Asia: The PERCH multi-country case-control study. Lancet 2019;394(10200):757-779. https://doi.org/10.1016/S0140-6736(19)30721-4
50. Schaaf HS, Marais BJ, Whitelaw A, et al. Culture-confirmed childhood tuberculosis in Cape Town, South Africa: A review of 596 cases. BMC Infect Dis 2007;7:140. https://doi.org/10.1186/1471-2334-7-140
51. Fairlie L, Beylis NC, Reubenson G, Moore DP, Madhi SA. High prevalence of childhood multi-drug resistant tuberculosis in Johannesburg, South Africa: A cross sectional study. BMC Infect Dis 2011;11:28. https://doi.org/10.1186/1471-2334-11-28
52. Moore BK, Anyalechi E, van der Walt M, et al. Epidemiology of drug-resistant tuberculosis among children and adolescents in South Africa, 2005 - 2010. Int J Tuberc Lung Dis 2015;19(6):663-669. https://doi.org/10.5588/ijtld.14.0879
53. Moore DP, Higdon MM, Hammitt LL, et al. The incremental value of repeated induced sputum and gastric aspirate samples for the diagnosis of pulmonary tuberculosis in young children with acute community-acquired pneumonia. Clin Infect Dis 2017;64(Suppl 3):S309-S316. https://doi.org/10.1093/cid/cix099
54. Michelow IC, Olsen K, Lozano J, et al. Epidemiology and clinical characteristics of community-acquired pneumonia in hospitalized children. Pediatrics 2004;113(4):701-707. https://doi.org/10.1542/peds.113.4.701
55. Blyth CC, Webb SA, Kok J, et al. The impact of bacterial and viral co-infection in severe influenza. Influenza Other Respir Viruses 2013;7(2):168-176. https://doi.org/10.1111/j.1750-2659.2012.00360.x
56. Scott JA, English M. What are the implications for childhood pneumonia of successfully introducing Hib and pneumococcal vaccines in developing countries? PLoS Med 2008;5(4):e86. https://doi.org/10.1371/journal.pmed.0050086
57. Cohen C, Walaza S, Moyes J, et al. Epidemiology of severe acute respiratory illness (SARI) among adults and children aged ≥5 years in a high HIV-prevalence setting, 2009-2012. PloS One 2015;10(2):e0117716. https://doi.org/10.1371/journal.pone.0117716
58. Jain S, Finelli L. Community-acquired pneumonia among U.S. children. New Engl J Med 2015;372(22):2167-2168. https://doi.org/10.1056/NEJMc1504028
59. Benet T, Sanchez Picot V, Messaoudi M, et al. Microorganisms associated with pneumonia in children <5 years of age in developing and emerging countries: The GABRIEL Pneumonia Multicenter, Prospective, Case-Control Study. Clin Infect Dis 2017;65(4):604-612. https://doi.org/10.1093/cid/cix378
60. le Roux DM, Zar HJ. Community-acquired pneumonia in children - a changing spectrum of disease. Pediatr Radiol 2017;47(11):1392-1398. https://doi.org/10.1007/s00247-017-3827-8
61. Moore DP, Baillie VL, Mudau A, et al. The etiology of pneumonia in HIV-uninfected South African children: Findings from the Pneumonia Etiology Research for Child Health (PERCH) Study. In press. 2020.
62. Moore DP, Baillie VL, Mudau A, et al. The etiology of pneumonia in HIV-1-infected South African children: Findings from the Pneumonia Etiology Research for Child Health (PERCH) Study. In press. 2020.
63. Zampoli M, Kappos A, Wolter N, et al. Etiology and incidence of pleural empyema in South African children. Pediatr Infect Dis J 2015;34(12):1305-1310. https://doi.org/10.1097/INF.0000000000000880
64. Krenke K, Urbankowska E, Urbankowski T, Lange J, Kulus M. Clinical characteristics of 323 children with parapneumonic pleural effusion and pleural empyema due to community acquired pneumonia. J InfectChemo 2016;22(5):292-297. https://doi.org/10.1016/j.jiac.2016.01.016
65. Krenke K, Sadowy E, Podsiadły E, Hryniewicz W, Demkow U, Kulus M. Etiology of parapneumonic effusion and pleural empyema in children. The role of conventional and molecular microbiological tests. RespMed 2016;116:28-33. https://doi.org/10.1016/j.rmed.2016.05.009
66. Zar HJ, Apolles P, Argent A, et al. The etiology and outcome of pneumonia in human immunodeficiency virus-infected children admitted to intensive care in a developing country. Pediatr Crit Care Med 2001;2(2):108-112. https://doi.org/10.1097/00130478-200104000-00003
67. McNally LM, Jeena PM, Gajee K, et al. Effect of age, polymicrobial disease, and maternal HIV status on treatment response and cause of severe pneumonia in South African children: A prospective descriptive study. Lancet 2007;369(9571):1440-1451. https://doi.org/10.1016/S0140-6736(07)60670-9
68. Chawana R, Baillie V, Izu A, et al. Potential of minimally invasive tissue sampling for attributing specific causes of childhood deaths in South Africa: A pilot, epidemiological study. Clin Infect Dis 2019;69(Suppl 4):S361-S373. https://doi.org/10.1093/cid/ciz550
69. Jeena PM, Pillay P, Pillay T, Coovadia HM. Impact of HIV-1 co-infection on presentation and hospital-related mortality in children with culture proven pulmonary tuberculosis in Durban, South Africa. Int J Tuberc Lung Dis 2002;6(8):672-678.
70. Moore DP, Klugman KP, Madhi SA. Role of Streptococcus pneumoniae in hospitalization for acute community-acquired pneumonia associated with culture-confirmed Mycobacterium tuberculosis in children: A pneumococcal conjugate vaccine probe study. Pediatr Infect Dis J 2010;29(12):1099-1104. https://doi.org/10.1097/inf.0b013e3181eaefff
71. Martinez L, le Roux DM, Barnett W, Stadler A, Nicol MP, Zar HJ. Tuberculin skin test conversion and primary progressive tuberculosis disease in the first 5 years of life: A birth cohort study from Cape Town, South Africa. Lancet Child Adolesc Health 2018;2:46-55. https://doi.org/10.1016/S2352-4642(17)30149-9
72. Shi T, McLean K, Campbell H, Nair H. Aetiological role of common respiratory viruses in acute lower respiratory infections in children under five years: A systematic review and meta-analysis. J Glob Health 2015;5(1):010408. https://doi.org/10.7189/jogh.05.010408
73. McAnerney JM, Cohen C, Moyes J, et al. Twenty-five years of outpatient influenza surveillance in South Africa, 1984-2008. J Infect Dis 2012;206(Suppl 1):S153-S158. https://doi.org/10.1093/infdis/jis575
74. Kyeyagalire R, Tempia S, Cohen AL, et al. Hospitalizations associated with influenza and respiratory syncytial virus among patients attending a network of private hospitals in South Africa, 2007-2012. BMC Infect Dis 2014;14:694. https://doi.org/10.1186/s12879-014-0694-x
75. Paules C, Subbarao K. Influenza. Lancet 2017;390(10095):697-708. https://doi.org/10.1016/S0140-6736(17)30129-0
76. Wei P, Cai Z, Hua J, et al. Pains and Gains from China's experiences with emerging epidemics: From SARS to H7N9. Biomed Res Int;2016:5717108. https://doi.org/10.1155/2016/5717108
77. Zumla A, Hui DS, Perlman S. Middle East respiratory syndrome. Lancet 2015;386(9997):995-1007. https://doi.org/10.1016/S0140-6736(15)60454-8
78. Huang C, Wang Y, Li X, et al. Clinical features of patients infected with 2019 novel coronavirus in Wuhan, China. Lancet 2020;395(10223):497-506. https://doi.org/10.1016/S0140-6736(20)30183-5
79. Chen ZM, Fu JF, Shu Q, et al. Diagnosis and treatment recommendations for pediatric respiratory infection caused by the 2019 novel coronavirus. World J Pediatr 2020 [Epub ahead of print.] https://doi.org/10.1007/s12519-020-00345-5
80. Department of Health, National Institute for Communicable Diseases. Clinical management of suspected or confirmed COVID-19 disease. Version 4. Johannesburg: Department of Health, National Institute for Communicable Diseases; 2020.
81. Pretorius MA, Madhi SA, Cohen C, et al. Respiratory viral coinfections identified by a 10-plex real-time reverse-transcription polymerase chain reaction assay in patients hospitalized with severe acute respiratory illness--South Africa, 2009-2010. J Infect Dis 2012;206(Suppl 1):S159-S165. https://doi.org/10.1093/infdis/jis538
82. van Gageldonk-Lafeber AB, Wever PC, van der Lubben IM, et al. The aetiology of community-acquired pneumonia and implications for patient management. Neth J Med 2013;71(7):418-425.
83. Brealey JC, Sly PD, Young PR, Chappell KJ. Viral bacterial co-infection of the respiratory tract during early childhood. FEMS Microbiol Let 2015;362(10):fnv062. https://doi.org/10.1093/femsle/fnv062
84. Coetzee S, Morrow BM, Argent AC. Measles in a South African paediatric intensive care unit: Again! J Paediatr Child Health 2014;50(5):379-385. https://doi.org/10.1111/jpc.12486
85. Dias FM, Marcal F, Oliveira J, Povoas M, Mouzinho A, Marques JG. Exuberant varicella-zoster exanthema and pneumonia as clinical clue for HIV infection. J Pediatrics 2015;166(1):199. https://doi.org/10.1016/j.jpeds.2014.09.047
86. Aebi C, Ahmed A, Ramilo O. Bacterial complications of primary varicella in children. Clinical Infect Dis 1996;23(4):698-705. https://doi.org/10.1093/clinids/23.4.698
87. Moss WJ. Measles. Lancet 2017;390(10111):2490-2502. https://doi.org/10.1016/S0140-6736(17)31463-0
88. Hussey H, Abdullahi L, Collins J, Muloiwa R, Hussey G, Kagina B. Varicella zoster virus-associated morbidity and mortality in Africa - a systematic review. BMC Infect Dis 2017;17(1):717. https://doi.org/10.1186/s12879-017-2815-9
89. Jeena PM, Coovadia HM, Chrystal V. Pneumocystis carinii and cytomegalovirus infections in severely ill, HIV-infected African infants. Ann Trop Paed 1996;16(4):361-368. https://doi.org/10.1080/02724936.1996.11747852
90. Zar HJ, Hanslo D, Tannenbaum E, et al. Aetiology and outcome of pneumonia in human immunodeficiency virus-infected children hospitalized in South Africa. Acta Paediatrica 2001;90(2):119-125.
91. Ruffini DD, Madhi SA. The high burden of Pneumocystis carinii pneumonia in African HIV-1-infected children hospitalized for severe pneumonia. AIDS 2002;16(1):105-112. https://doi.org/10.1097/00002030-200201040-00013
92. Jeena PM, Bobat B, Thula SA, Adhikari M. Children with Pneumocystis jiroveci pneumonia and acute hypoxaemic respiratory failure admitted to a PICU, Durban, South Africa. Arch Dis Child 2008;93(6):545. https://doi.org/10.1136/adc.2006.105890
93. Graham SM, Mtitimila EI, Kamanga HS, Walsh AL, Hart CA, Molyneux ME. Clinical presentation and outcome of Pneumocystis carinii pneumonia in Malawian children. Lancet 2000;355(9201):369-373. https://doi.org/10.1016/S0140-6736(98)11074-7
94. Goussard P, Kling S, Gie RP, et al. CMV pneumonia in HIV-infected ventilated infants. Pediatr Pulmonol 2010;45(7):650-655. https://doi.org/10.1002/ppul.21228

95. Jeena PM, Govender K, Parboosing R, Adhikari M. The significance of cytomegalovirus in children with pneumonia admitted for mechanical ventilation. Int J Tuberc Lung Dis 2017;21(12):1230-1236. https://doi.org/10.5588/ijtld.17.0026
96. Cox JA, Lukande RL, Lucas S, Nelson AM, Van Marck E, Colebunders R. Autopsy causes of death in HIV-positive individuals in sub-Saharan Africa and correlation with clinical diagnoses. AIDS Rev 2010;12(4):183-194.
97. Teira P, Battiwalla M, Ramanathan M, et al. Early cytomegalovirus reactivation remains associated with increased transplant-related mortality in the current era: A CIBMTR analysis. Blood 2016;127(20):2427-2438. https://doi.org/10.1182/blood-2015-11-679639
98. Paulsen G, Cumagun P, Mixon E, Fowler K, Feig D, Shimamura M. Cytomegalovirus and Epstein-Barr virus infections among pediatric kidney transplant recipients at a center using universal Valganciclovir prophylaxis. Pediatr Transplant 2019;23(3):e13382. https://doi.org/10.1111/petr.13382
99. Carrim M. Identification and prevalence of bacteria causing atypical pneumonia in patients with severe respiratory illness and influenza-like illness in South Africa, 2012-2013. Faculty of Health Sciences. Johannesburg: University of the Witwatersrand; 2015:108. Available at http://wiredspace.wits.ac.za/handle/10539/19566
100. Carrim M, Wolter N, Benitez AJ, et al. Epidemiology and molecular identification and characterization of Mycoplasma pneumoniae, South Africa, 2012-2015. Emerg Infect Dis 2018;24(3):506-513. https://doi.org/10.3201/eid2403.162052
101. Tramper-Stranders GA. Childhood community-acquired pneumonia: A review of etiology- and antimicrobial treatment studies. Paediatr Respir Rev 2018;26:41-48. https://doi.org/10.1016/j.prrv.2017.06.013
102. Lorgelly PK, Atkinson M, Lakhanpaul M, et al. Oral versus IV antibiotics for community-acquired pneumonia in children: A cost-minimisation analysis. Eur Respir J 2010;35(4):858-864. https://doi.org/10.1183/09031936.00087209
103. Nascimento-Carvalho CM. Community-acquired pneumonia among children: The latest evidence for an updated management. J Pediatr (Rio J) 2020;96(Suppl 1):29-38. https://doi.org/10.1016/j.jped.2019.08.003
104. American Academy of Pediatrics. Red Book: 2018 Report of the Committee on Infectious Diseases. 31st ed. Elk Grove Village, IL: AAP, 2018.
105. Mathur S, Fuchs A, Bielicki J, van den Anker J, Sharland M. Antibiotic use for community-acquired pneumonia in neonates and children: WHO evidence review. Paediatr Int Child Health 2018;38(Suppl 1):S66-S75. https://doi.org/10.1080/20469047.2017.1409455
106. National Department of Health. Standard Treatment Guidelines and Essential Medicines List for South African Hospital Level Paediatrics. Pretoria: NDoH, 2017.
107. Stern A, Skalsky K, Avni T, Carrara E, Leibovici L, Paul M. Corticosteroids for pneumonia. Cochrane Database Syst Rev 2017;(12):CD007720. https://doi.org/10.1002/14651858.CD007720.pub3
108. Goussard P, Gie R. Airway involvement in pulmonary tuberculosis. Paediatr Respir Rev 2007;8(2):118-123. https://doi.org/10.1016/j.prrv.2007.04.007
109. Hussey GD, Klein M. A randomized, controlled trial of vitamin A in children with severe measles. N Engl J Med 1990;323(3):160-164. https://doi.org/10.1056/NEJM199007193230304
110. Kirkwood BR, Gove S, Rogers S, Lob-Levyt J, Arthur P, Campbell H. Potential interventions for the prevention of childhood pneumonia in developing countries: A systematic review. Bull World Health Organ 1995;73(6):793-798.
111. Mehta S, Fawzi W. Effects of vitamins, including vitamin A, on HIV/AIDS patients. Vitamin Hormone 2007;75:355-383. https://doi.org/10.1016/s0083-6729(06)75013-0
112. Li W, Guo L, Li H, et al. Polymorphism rs2239185 in vitamin D receptor gene is associated with severe community-acquired pneumonia of children in Chinese Han population: A case-control study. Eur J Paediatr 2015;174(5):621-629. https://doi.org/10.1007/s00431-014-2433-0
113. Esposito S, Lelii M. Vitamin D and respiratory tract infections in childhood. BMC Infect Dis 2015;15:487. https://doi.org/10.1186/s12879-015-1196-1
114. Somnath SH, Biswal N, Chandrasekaran V, Jagadisan B, Bobby Z. Therapeutic effect of vitamin D in acute lower respiratory infection: A randomized controlled trial. Clin Nutr ESPEN 2017;20:24-28. https://doi.org/10.1016/j.clnesp.2017.02.003
115. Das RR, Singh M, Naik SS. Vitamin D as an adjunct to antibiotics for the treatment of acute childhood pneumonia. Cochrane Database Syst Rev 2018;(7):CD011597. https://doi.org/10.1002/14651858.CD011597.pub2
116. Smyth A, Carty H, Hart CA. Clinical predictors of hypoxaemia in children with pneumonia. Ann Trop Paediatr 1998;18(1):31-40.
117. Gie R, Madhi SA, Jeena P, Argent A, Zar HJ. Diagnosis and management of community-acquired pneumonia in childhood - South African Thoracic Society guidelines: Clinical guideline. South Afr J Epidemiol Infect 2009;24(1):25-36.
118. Coletti KD, Bagdure DN, Walker LK, Remy KE, Custer JW. High-flow nasal cannula utilization in pediatric critical care. Respir Care 2017;62(8):1023-1029. https://doi.org/10.4187/respcare.05153
119. Davison M, Watson M, Wockner L, Kinnear F. Paediatric high-flow nasal cannula therapy in children with bronchiolitis: A retrospective safety and efficacy study in a non-tertiary environment. Emerg Med Austr 2017;29(2):198-203. https://doi.org/10.1111/1742-6723.12741
120. Bressan S, Balzani M, Krauss B, Pettenazzo A, Zanconato S, Baraldi E. High-flow nasal cannula oxygen for bronchiolitis in a pediatric ward: A pilot study. Eur J Paediatr 2013;172(12):1649-1656. https://doi.org/10.1007/s00431-013-2094-4
121. Lopes CLS, Piva JP. Fluid overload in children undergoing mechanical ventilation. Rev Brasil Terap Intens 2017;29(3):346-353. https://doi.org/10.5935/0103-507x.20170045
122. Ingelse SA, Wiegers HM, Calis JC, van Woensel JB, Bem RA. Early fluid overload prolongs mechanical ventilation in children with viral-lower respiratory tract disease. Pediatr Crit Care Med 2017;18(3):e106-e111. https://doi.org/10.1097/pcc.0000000000001060
123. Willson DF, Thomas NJ, Tamburro R, et al. The relationship of fluid administration to outcome in the pediatric calfactant in acute respiratory distress syndrome trial. Pediatr Crit Care Med 2013;14(7):666-672. https://doi.org/10.1097/PCC.0b013e3182917cb5
124. Tagarro A, Martin MD, Del-Amo N, et al. Hyponatremia in children with pneumonia rarely means SIADH. Paediatr Child Health 2018;23(7):e126-e133. https://doi.org/10.1093/pch/pxy003
125. Don M, Valerio G, Korppi M, Canciani M. Hyponatremia in pediatric community-acquired pneumonia. Pediatr Nephrol 2008;23(12):2247-2253. https://doi.org/10.1007/s00467-008-0910-2
126. Wrotek A, Jackowska T. Hyponatremia in children hospitalized due to pneumonia. Adv Exp Med Biol 2013;788:103-108. https://doi.org/10.1007/978-94-007-6627-3_16
127. Sakellaropoulou A, Hatzistilianou M, Eboriadou M, Athanasiadou-Piperopoulou F. Hyponatraemia in cases of children with pneumonia. Arch Med Sci 2010;6(4):578-583. https://doi.org/10.5114/aoms.2010.14471
128. Shann F, Germer S. Hyponatraemia associated with pneumonia or bacterial meningitis. Arch Dis Child 1985;60(10):963-966.
129. Ramanathan S, Kumar P, Mishra K, Dutta AK. Isotonic versus hypotonic parenteral maintenance fluids in very severe pneumonia. Ind J Pediatr 2016;83(1):27-32. https://doi.org/10.1007/s12098-015-1791-6
130. El-Radhi AS. Fever management: Evidence vs current practice. World J Clin Pediatr 2012;1(4):29-33. https://doi.org/10.5409/wjcp.v1.i4.29
131. Chiappini E, Venturini E, Remaschi G, et al. 2016 update of the Italian Pediatric Society guidelines for management of fever in children. J Pediatr 2017;180:177-183. https://doi.org/10.1016/j.jpeds.2016.09.043
132. Soda EA, Barskey AE, Shah PP, et al. Vital signs: Health care-associated Legionnaires' disease surveillance data from 20 states and a large metropolitan area - United States, 2015. MMWR Morbid Mortal Wkly Rep 2017;66(22):584-589. https://doi.org/10.15585/mmwr.mm6622e1
133. Chang CC, Cheng AC, Chang AB. Over-the-counter (OTC) medications to reduce cough as an adjunct to antibiotics for acute pneumonia in children and adults. Cochrane Database Syst Rev 2014;(3):CD006088. https://doi.org/10.1002/14651858.CD006088.pub4
134. Warnock L, Gates A. Chest physiotherapy compared to no chest physiotherapy for cystic fibrosis. Cochrane Database Syst Rev 2015;(12):CD001401. https://doi.org/10.1002/14651858.CD001401.pub3
135. Lestari NE, Nurhaeni N, Chodidjah S. The combination of nebulization and chest physiotherapy improved respiratory status in children with pneumonia. Enferm Clin 2018;28(Suppl 1):19-22. https://doi.org/10.1016/S1130-8621(18)30029-9
136. Gilchrist FJ. Is the use of chest physiotherapy beneficial in children with community acquired pneumonia? Arch Dis Child 2008;93(2):176-178. https://doi.org/10.1136/adc.2007.127290
137. Damiani F, Adasme R. What is the effect of chest physiotherapy in hospitalized children with pneumonia? Medwave 2015;15(Suppl 2):e6287-e6287. https://doi.org/10.5867/medwave.2015.6287
138. Alonso-Quintela P, Oulego-Erroz I, Rodriguez-Blanco S, Muniz-Fontan M, Lapena-Lopez-de Armentia S, Rodriguez-Nunez A. Location of the central venous catheter tip with bedside ultrasound in young children: Can we eliminate the need for chest radiography? Pediatr Crit Care Med 2015;16(9):e340-e345. https://doi.org/10.1097/PCC.0000000000000491
139. Bassat Q, Lanaspa M, Machevo S, et al. Hypoxaemia in Mozambican children <5 years of age admitted to hospital with clinical severe pneumonia: Clinical features and performance of predictor models. Trop Med Int Health 2016;21(9):1147-1156. https://doi.org/10.1111/tmi.12738
140. Jayashree M, KiranBabu HB, Singhi S, Nallasamy K. Use of nasal bubble CPAP in children with hypoxemic clinical pneumonia - report from a resource limited set-up. J Trop Pediatr 2016;62(1):69-74. https://doi.org/10.1093/tropej/fmv063
141. Machen HE, Mwanza ZV, Brown JK, et al. Outcomes of patients with respiratory distress treated with bubble CPAP on a pediatric ward in Malawi. J Trop Pediatr 2015;61(6):421-427. https://doi.org/10.1093/tropej/fmv052
142. Milesi C, Essouri S, Pouyau R, et al. High flow nasal cannula (HFNC) versus nasal continuous positive airway pressure (nCPAP) for the initial respiratory management of acute viral bronchiolitis in young infants: A multicenter randomized controlled trial (TRAMONTANE study). Intens Care Med 2017;43(2):209-216. https://doi.org/10.1007/s00134-016-4617-8

143. Javouhey E, Barats A, Richard N, Stamm D, Floret D. Non-invasive ventilation as primary ventilatory support for infants with severe bronchiolitis. Intens Care Med 2008;34(9):1608-1614. https://doi.org/10.1007/s00134-008-1150-4
144. Kawaguchi A, Yasui Y, deCaen A, Garros D. The clinical impact of heated humidified high-flow nasal cannula on pediatric respiratory distress. Pediatr Crit Care Med 2017;18(2):112-119. https://doi.org/10.1097/pcc.0000000000000985
145. Bateman ST, Borasino S, Asaro LA, et al. Early high-frequency oscillatory ventilation in pediatric acute respiratory failure. A propensity score analysis. Am J Resp Crit Care Med 2016;193(5):495-503. https://doi.org/10.1164/rccm.201507-1381OC
146. Khemani RG, Parvathaneni K, Yehya N, Bhalla AK, Thomas NJ, Newth CJL. Positive end-expiratory pressure lower than the ARDS network protocol is associated with higher pediatric acute respiratory distress syndrome mortality. Am J Resp Crit Care Med 2018;198(1):77-89. https://doi.org/10.1164/rccm.201707-1404OC
147. Mikhailov TA, Kuhn EM, Manzi J, et al. Early enteral nutrition is associated with lower mortality in critically ill children. J Parenter Enteral Nutr 2014;38(4):459-466. https://doi.org/10.1177/0148607113517903
148. Wilson B, Typpo K. Nutrition: A primary therapy in pediatric acute respiratory distress syndrome. Front Pediatr 2016;4:108. https://doi.org/10.3389/fped.2016.00108
149. Mehta NM, Bechard LJ, Cahill N, et al. Nutritional practices and their relationship to clinical outcomes in critically ill children – an international multicenter cohort study. Crit Care Med 2012;40(7):2204-2211. https://doi.org/10.1097/CCM.0b013e31824e18a8
150. Fivez T, Kerklaan D, Mesotten D, et al. Early versus late parenteral nutrition in critically ill children. N Engl J Med 2016;374(12):1111-1122. https://doi.org/10.1056/NEJMoa1514762
151. Vanhorebeek I, Verbruggen S, Casaer MP, et al. Effect of early supplemental parenteral nutrition in the paediatric ICU: A preplanned observational study of post-randomisation treatments in the PEPaNIC trial. Lancet Respir Med 2017;5(6):475-483. https://doi.org/10.1016/s2213-2600(17)30186-8
152. Sochet AA, McGee JA, October TW. Oral nutrition in children with bronchiolitis on high-flow nasal cannula is well tolerated. Hosp Pediatr 2017;7(5):249-255. https://doi.org/10.1542/hpeds.2016-0131
153. Slain KN, Martinez-Schlurmann N, Shein SL, Stormorken A. Nutrition and high-flow nasal cannula respiratory support in children with bronchiolitis. Hosp Pediatr 2017;7(5):256-262. https://doi.org/10.1542/hpeds.2016-0194
154. Schuetz P, Muller B, Christ-Crain M, et al. Procalcitonin to initiate or discontinue antibiotics in acute respiratory tract infections. Cochrane Rev J 2013;8(4):1297-1371. https://doi.org/10.1002/ebch.1927
155. Timsit JF, Bassetti M, Cremer O, et al. Rationalizing antimicrobial therapy in the ICU: A narrative review. Intens Care Med 2019;45(2):172-189. https://doi.org/10.1007/s00134-019-05520-5
156. Morrow B. Chest Physiotherapy in the pediatric intensive care unit. J Pediatr Intens Care 2015;4:174-181.
157. Corten L, Jelsma J, Morrow BM. Chest physiotherapy in children with acute bacterial pneumonia. S Afr J Physiother 2015;71(1):256. https://doi.org/10.4102/sajp.v71i1.256
158. Wright AL, Bauer M, Naylor A, Sutcliffe E, Clark L. Increasing breastfeeding rates to reduce infant illness at the community level. Pediatrics 1998;101(5):837-844. https://doi.org/10.1542/peds.101.5.837
159. Lamberti LM, Zakarija-Grkovic I, Fischer Walker CL, et al. Breastfeeding for reducing the risk of pneumonia morbidity and mortality in children under two: A systematic literature review and meta-analysis. BMC Public Health 2013;13(Suppl 3):S18. https://doi.org/10.1186/1471-2458-13-S3-S18
160. Doherty T, Sanders D, Goga A, Jackson D. Implications of the new WHO guidelines on HIV and infant feeding for child survival in South Africa. Bull World Health Organ 2011;89(1):62-67. https://doi.org/10.2471/BLT.10.079798
161. Kuhn L, Kroon M. Breastfeeding and the 2015 South African guidelines for prevention of mother-to-child transmission of HIV. S Afr J HIV Med 2015;16(1). https://doi.org/10.4102/sajhivmed.v16i1.377
162. National Department of Health. Road to Health Booklet 2018. http://www.health.gov.za/index.php/shortcodes/2015-03-29-10-42-47/2015-04-30-08-29-27/2015-04-30-08-33-30/category/545-immunisation-survey?download=3388:road-to-health-booklet. (Accessed xx)
163. Wayse V, Yousafzai A, Mogale K, Filteau S. Association of subclinical vitamin D deficiency with severe acute lower respiratory infection in Indian children under 5 y. Eur J Clin Nutr 2004;58(4):563-567. https://doi.org/10.1038/sj.ejcn.1601845
164. Charan J, Goyal JP, Saxena D, Yadav P. Vitamin D for prevention of respiratory tract infections: A systematic review and meta-analysis. J Pharmacol Pharmacother 2012;3(4):300-303. https://doi.org/10.4103/0976-500X.103685
165. Choudhary N, Gupta P. Vitamin D supplementation for severe pneumonia – a randomized controlled trial. Indian Pediatr 2012;49(6):449-454. https://doi.org/10.1007/s13312-012-0073-x
166. Membership of Scientific Advisory Committee on Nutrition: Working Group on Vitamin D. Vitamin D and Health. Scientific Advisory Committee on Nutrition; 2016. https://assets.publishing.service.gov.uk/government/uploads/system/uploads/attachment_data/file/537616/SACN_Vitamin_D_and_Health_report.pdf (Acessed xx)
167. Theodoratou E, Al-Jilaihawi S, Woodward F, et al. The effect of case management on childhood pneumonia mortality in developing countries. Int J Epidemiol 2010;39(Suppl 1):i155-i171. https://doi.org/10.1093/ije/dyq032
168. Lee GY, Han SN. The role of vitamin E in immunity. Nutrients 2018;10(11):1614. https://doi.org/10.3390/nu10111614
169. Bhandari N, Bahl R, Taneja S, et al. Effect of routine zinc supplementation on pneumonia in children aged 6 months to 3 years: Randomised controlled trial in an urban slum. BMJ 2002;324(7350):1358. https://doi.org/10.1136/bmj.324.7350.1358
170. Bhutta ZA, Black RE, Brown KH, et al. Prevention of diarrhea and pneumonia by zinc supplementation in children in developing countries: Pooled analysis of randomized controlled trials. J Pediatr 1999;135(6):689-697. https://doi.org/10.1016/s0022-3476(99)70086-7
171. Gibbs K, Collaco JM, McGrath-Morrow SA. Impact of tobacco smoke and nicotine exposure on lung development. Chest 2016;149(2):552-561. https://doi.org/10.1378/chest.15-1858
172. Steyn K, Yach D, Stander I, Fourie JM. Smoking in urban pregnant women in South Africa. S Afr Med J 1997;87(4):460-463.
173. Baumgartner J, Schauer JJ, Ezzati M, et al. Patterns and predictors of personal exposure to indoor air pollution from biomass combustion among women and children in rural China. Indoor Air 2011;21(6):479-488. https://doi.org/10.1111/j.1600-0668.2011.00730.x
174. Admasie A, Kumie A, Worku A. Children under five from houses of unclean fuel sources and poorly ventilated houses have higher odds of suffering from acute respiratory infection in Wolaita-Sodo, Southern Ethiopia: A case-control study. J Environ Public Health 2018;2018:9320603. https://doi.org/10.1155/2018/9320603
175. American Academy of Pediatrics Committee on Infectious Diseases. Infection prevention and control in pediatric ambulatory settings. Pediatrics 2007;120(3):650-665. https://doi.org/10.1542/peds.2007-1734
176. Hellewell J, Abbott S, Gimma A, et al. Feasibility of controlling COVID-19 outbreaks by isolation of cases and contacts. Lancet Glob Health 2020;8(4):E488-E496. https://doi.org/10.1016/S2214-109X(20)30074-7
177. Little P, Stuart B, Hobbs FDR, et al. An internet-delivered handwashing intervention to modify influenza-like illness and respiratory infection transmission (PRIMIT): A primary care randomised trial. Lancet 2015;386(10004):1631-1639. https://doi.org/https://doi.org/10.1016/S0140-6736(15)60127-1
178. Willmott M, Nicholson A, Busse H, MacArthur GJ, Brookes S, Campbell R. Effectiveness of hand hygiene interventions in reducing illness absence among children in educational settings: A systematic review and meta-analysis. Arch Dis Childh 2016;101(1):42-50. https://doi.org/10.1136/archdischild-2015-308875
179. Walker JH. Pediatric influenza and 'universal respiratory etiquette': Preparing and protecting your staff and the public. J Emerg Nurs 2004;30(4):367-370. https://doi.org/10.1016/j.jen.2004.04.006
180. Chu DK, Akl EA, Duda S, Solo K, Yaacoub S, Schünemann HJ. Physical distancing, face masks, and eye protection to prevent person-to-person transmission of SARS-CoV-2 and COVID-19: A systematic review and meta-analysis. Lancet 2020;395(10242):1973-1987. https://doi.org/10.1016/s0140-6736(20)31142-9
181. MacIntyre CR, Wang Q. Physical distancing, face masks, and eye protection for prevention of COVID-19. Lancet 2020;395. https://doi.org/10.1016/s0140-6736(20)31183-1
182. Hoest C, Seidman JC, Lee G, et al. Vaccine coverage and adherence to EPI schedules in eight resource poor settings in the MAL-ED cohort study. Vaccine 2017;35(3):443-451. https://doi.org/10.1016/j.vaccine.2016.11.075
183. Madhi SA, Cumin E, Klugman KP. Defining the potential impact of conjugate bacterial polysaccharide-protein vaccines in reducing the burden of pneumonia in human immunodeficiency virus type 1-infected and -uninfected children. Pediatr Infect Dis J 2002;21(5):393-399. https://doi.org/10.1097/00006454-200205000-00009
184. Madhi SA, Kuwanda L, Cutland C, Holm A, Käyhty H, Klugman KP. Quantitative and qualitative antibody response to pneumococcal conjugate vaccine among African human immunodeficiency virus-infected and uninfected children. Pediatr Infect Dis J 2005;24(5):410-416. https://doi.org/10.1097/01.inf.0000160942.84169.14
185. Madhi SA, Kuwanda L, Saarinen L, et al. Immunogenicity and effectiveness of Haemophilus influenzae type b conjugate vaccine in HIV infected and uninfected African children. Vaccine 2005;23(48-49):5517-5525. https://doi.org/10.1016/j.vaccine.2005.07.038
186. Klugman KP, Madhi SA, Huebner RE, et al. A trial of a 9-valent pneumococcal conjugate vaccine in children with and those without HIV infection. N Engl J Med 2003;349(14):1341-1348. https://doi.org/10.1056/NEJMoa035060
187. Madhi SA, Adrian P, Kuwanda L, et al. Long-term immunogenicity and efficacy of a 9-valent conjugate pneumococcal vaccine in human immunodeficient virus infected and non-infected children in the absence of a booster dose of vaccine. Vaccine 2007;25(13):2451-2457. https://doi.org/10.1016/j.vaccine.2006.09.019

188. Madhi SA, Adrian P, Cotton MF, et al. Effect of HIV infection status and antiretroviral treatment on quantitative and qualitative antibody responses to pneumococcal conjugate vaccine in infants. J Infect Dis 2010;202(3):355-361. https://doi.org/10.1086/653704
189. Simani OE, Izu A, Violari A, et al. Effect of HIV-1 exposure and antiretroviral treatment strategies in HIV-infected children on immunogenicity of vaccines during infancy. AIDS 2014;28(4):531-541. https://doi.org/10.1097/QAD.0000000000000127
190. Dzanibe S, Adrian PV, Mlacha SZK, Dangor Z, Kwatra G, Madhi SA. Reduced transplacental transfer of group B streptococcus surface protein antibodies in HIV-infected mother-newborn dyads. J Infect Dis 2017;215(3):415-419. https://doi.org/10.1093/infdis/jiw566
191. Jallow S, Madhi SA. Pneumococcal conjugate vaccine in HIV-infected and HIV-exposed, uninfected children. Expert Rev Vaccines 2017;16(5):453-465. https://doi.org/10.1080/14760584.2017.1307740
192. Simani OE, Izu A, Nunes MC, et al. Effect of HIV exposure and timing of antiretroviral therapy initiation on immune memory responses to diphtheria, tetanus, whole cell pertussis and hepatitis B vaccines. Expert Rev Vaccines 2019;18(1):95-104. https://doi.org/10.1080/14760584.2019.1547195
193. Oliwa JN, Karumbi JM, Marais BJ, Madhi SA, Graham SM. Tuberculosis as a cause or comorbidity of childhood pneumonia in tuberculosis-endemic areas: A systematic review. Lancet Respir Med 2015;3(3):235-243. https://doi.org/10.1016/S2213-2600(15)00028-4
194. Colditz GA, Brewer TF, Berkey CS, et al. Efficacy of BCG vaccine in the prevention of tuberculosis. Meta-analysis of the published literature. JAMA 1994;271(9):698-702.
195. Brewer TF. Preventing tuberculosis with bacillus Calmette-Guérin vaccine: A meta-analysis of the literature. Clin Infect Dis 2000;31(Suppl 3):S64-S67. https://doi.org/10.1086/314072
196. Schaltz-Buchholzer F, Biering-Sørensen S, Lund N, et al. Early BCG vaccination, hospitalizations, and hospital deaths: Analysis of a secondary outcome in 3 randomized trials from Guinea-Bissau. J Infect Dis 2019;219(4):624-632. https://doi.org/10.1093/infdis/jiy544
197. Alicino C, Paganino C, Orsi A, et al. The impact of 10-valent and 13-valent pneumococcal conjugate vaccines on hospitalization for pneumonia in children: A systematic review and meta-analysis. Vaccine 2017;35(43):5776-5785. https://doi.org/10.1016/j.vaccine.2017.09.005
198. World Health Organization. 23-valent pneumococcal polysaccharide vaccine WHO position paper. Wkly Epidemiol Rec 2008;42:373-384.
199. Wang Y, Li J, Wang Y, Gu W, Zhu F. Effectiveness and practical uses of 23-valent pneumococcal polysaccharide vaccine in healthy and special populations. Hum Vaccin Immunother 2018;14(4):1003-1012. https://doi.org/10.1080/21645515.2017.1409316
200. Moore MR, Link-Gelles R, Schaffner W, et al. Effect of use of 13-valent pneumococcal conjugate vaccine in children on invasive pneumococcal disease in children and adults in the USA: Analysis of multisite, population-based surveillance. Lancet Infect Dis 2015;15(3):301-309. https://doi.org/10.1016/S1473-3099(14)71081-3
201. Berical AC, Harris D, Dela Cruz CS, Possick JD. Pneumococcal vaccination strategies. An update and perspective. Ann Am Thorac Soc 2016;13(6):933-944. https://doi.org/10.1513/AnnalsATS.201511-778FR
202. Tsaban G, Ben-Shimol S. Indirect (herd) protection, following pneumococcal conjugated vaccines introduction: A systematic review of the literature. Vaccine 2017;35(22):2882-2891. https://doi.org/10.1016/j.vaccine.2017.04.032
203. World Health Organization. Pneumococcal conjugate vaccines in infants and children under 5 years of age: WHO position paper.. Wkly Epidemiol Rec 2019;94(8):85-104.
204. Von Gottberg A, de Gouveia L, Madhi SA, et al. Impact of conjugate Haemophilus influenzae type b (Hib) vaccine introduction in South Africa. Bull World Health Organ 2006;84(10):811-818. https://doi.org/10.2471/blt.06.030361
205. Von Gottberg A, Cohen C, Whitelaw A, et al. Invasive disease due to Haemophilus influenzae serotype b ten years after routine vaccination, South Africa, 2003 - 2009. Vaccine 2012;30(3):565-571. https://doi.org/10.1016/j.vaccine.2011.11.066
206. Barger-Kamate B, Knoll MD, Kagucia EW, et al. Pertussis-associated pneumonia in infants and children from low- and middle-income countries participating in the PERCH Study. Clin Infect Dis 2016;63(Suppl 4):S187-S196. https://doi.org/10.1093/cid/ciw546
207. Soofie N, Nunes MC, Kgagudi P, et al. The burden of pertussis hospitalization in HIV-exposed and HIV-unexposed South African infants. Clin Infect Dis 2016;63(Suppl 4):S165-S173. https://doi.org/10.1093/cid/ciw545
208. Warfel JM, Zimmerman LI, Merkel TJ. Acellular pertussis vaccines protect against disease but fail to prevent infection and transmission in a nonhuman primate model. Proc Natl Acad Sci USA 2014;111(2):787-792. https://doi.org/10.1073/pnas.1314688110
209. Van den Biggelaar AHJ, Poolman JT. Predicting future trends in the burden of pertussis in the 21st century: Implications for infant pertussis and the success of maternal immunization. Expert Rev Vaccines 2016;15(1):69-80. https://doi.org/10.1586/14760584.2016.1105136
210. Marlow R, Kuriyakose S, Mesaros N, et al. A phase III, open-label, randomised multicentre study to evaluate the immunogenicity and safety of a booster dose of two different reduced antigen diphtheria-tetanus-acellular pertussis-polio vaccines, when co-administered with measles-mumps-rubella vaccine in 3 and 4-year-old healthy children in the UK. Vaccine 2018;36(17):2300-2306. https://doi.org/10.1016/j.vaccine.2018.03.021
211. Campbell H, Gupta S, Dolan GP, et al. Review of vaccination in pregnancy to prevent pertussis in early infancy. J Med Microbiol 2018;67(10):1426-1456. https://doi.org/10.1099/jmm.0.000829
212. Belongia EA, Simpson MD, King JP, et al. Variable influenza vaccine effectiveness by subtype: A systematic review and meta-analysis of test-negative design studies. Lancet Infect Dis 2016;16(8):942-951. https://doi.org/10.1016/S1473-3099(16)00129-8
213. Madhi SA, Dittmer S, Kuwanda L, et al. Efficacy and immunogenicity of influenza vaccine in HIV-infected children: A randomized, double-blind, placebo controlled trial. AIDS 2013;27(3):369-379. https://doi.org/10.1097/QAD.0b013e32835ab5b2
214. Grohskopf LA, Sokolow LZ, Broder KR, Walter EB, Fry AM, Jernigan DB. Prevention and control of seasonal influenza with vaccines: Recommendations of the Advisory Committee on Immunization Practices - United States, 2018 - 19 influenza season. MMWR Recomm Rep 2018;67(3):1-20. https://doi.org/10.15585/mmwr.rr6703a1
215. Nunes MC, Cutland CL, Jones S, et al. Duration of infant protection against influenza illness conferred by maternal immunization: Secondary analysis of a randomized clinical trial. JAMA Pediatr 2016;170(9):840-847. https://doi.org/10.1001/jamapediatrics.2016.0921
216. Omer SB, Clark DR, Aqil AR, et al. Maternal influenza immunization and prevention of severe clinical pneumonia in young infants: Analysis of randomized controlled trials conducted in Nepal, Mali and South Africa. Pediatr Infect Dis J 2018;37(5):436-440. https://doi.org/10.1097/INF.0000000000001914
217. Cohen C, Walaza S, Treurnicht FK, et al. In- and out-of-hospital mortality associated with seasonal and pandemic influenza and respiratory syncytial virus in South Africa, 2009 - 2013. Clin Infect Dis 2018;66(1):95-103. https://doi.org/10.1093/cid/cix740
218. World Health Organization. WHO Guidelines for Epidemic Preparedness and Response to Measles Outbreaks. Geneva: WHO, 1999.
219. Ntshoe GM, McAnerney JM, Archer BN, et al. Measles outbreak in South Africa: Epidemiology of laboratory-confirmed measles cases and assessment of intervention, 2009 - 2011. PLoS ONE 2013;8(2):e55682. https://doi.org/10.1371/journal.pone.0055682
220. Simani OE, Adrian PV, Violari A, et al. Effect of in-utero HIV exposure and antiretroviral treatment strategies on measles susceptibility and immunogenicity of measles vaccine. AIDS 2013;27(10):1583-1591. https://doi.org/10.1097/QAD.0b013e32835fae26
221. Jallow S, Cutland CL, Masbou AK, Adrian P, Madhi SA. Maternal HIV infection associated with reduced transplacental transfer of measles antibodies and increased susceptibility to disease. J Clin Virol 2017;94:50-56. https://doi.org/10.1016/j.jcv.2017.07.009
222. World Health Organization. Measles vaccines: WHO position paper. Wkly Epidemiol Rec 2017;92(17):205-228.
223. Mutsaerts EAML, Nunes MC, Bhikha S, et al. Immunogenicity and safety of an early measles vaccination schedule at 6 and 12 months of age in human immunodeficiency virus (HIV)-unexposed and HIV-exposed, uninfected South African children. J Infect Dis 2019;220(9):1529-1538. https://doi.org/10.1093/infdis/jiz348
224. Department of Health. 2019 ART Clinical Guidelines for the Management of HIV in Adults, Pregnancy, Adolescents, Children, Infants and Neonates. Pretoria: DoH, 2019.
225. Lockman S, Hughes M, Powis K, et al. Effect of co-trimoxazole on mortality in HIV-exposed but uninfected children in Botswana (the Mpepu study): A double-blind, randomised, placebo-controlled trial. Lancet Glob Health 2017;5(5):e491-e500. https://doi.org/10.1016/S2214-109X(17)30143-2
226. Daniels B, Coutsoudis A, Moodley-Govender E, et al. Effect of co-trimoxazole prophylaxis on morbidity and mortality of HIV-exposed, HIV-uninfected infants in South Africa: A randomised controlled, non-inferiority trial. Lancet Glob Health 2019;7(12):e1717-e1727. https://doi.org/10.1016/S2214-109X(19)30422-X
227. Schaaf HS, Cotton MF, Boon GPG, Jeena PM. Isoniazid preventive therapy in HIV-infected and -uninfected children (0 - 14 years) 2013. S Afr Med J 2013;103(10):714-715. https://doi.org/10.7196/SAMJ.7189
228. Zar HJ, Cotton MF, Strauss S, et al. Effect of isoniazid prophylaxis on mortality and incidence of tuberculosis in children with HIV: Randomised controlled trial. BMJ 2007;334(7585):136-136. https://doi.org/10.1136/bmj.39000.486400.55
229. Madhi SA, Nachman S, Violari A, et al. Primary isoniazid prophylaxis against tuberculosis in HIV-exposed children. N Engl J Med 2011;365(1):21-31. https://doi.org/10.1056/NEJMoa1011214
230. McIlleron H, Denti P, Cohn S, et al. Prevention of TB using rifampicin plus isoniazid reduces nevirapine concentrations in HIV-exposed infants. J Antimicrob Chemother 2017;72(7):2028-2034. https://doi.org/10.1093/jac/dkx112
231. Seddon JA, Hesseling AC, Finlayson H, et al. Preventive therapy for child contacts of multidrug-resistant tuberculosis: A prospective cohort study. Clin Infect Dis 2013;57(12):1676-1684. https://doi.org/10.1093/cid/cit655

232. Mofenson LM, Brady MT, Danner SP, et al. Guidelines for the prevention and treatment of opportunistic infections among HIV-exposed and HIV-infected children: Recommendations from CDC, the National Institutes of Health, the HIV Medicine Association of the Infectious Diseases Society of America, the Pediatric Infectious Diseases Society, and the American Academy of Pediatrics. MMWR Recomm Rep 2009;58(Rr-11):1-166.
233. IMpact-RSV Study Group. Palivizumab, a humanized respiratory syncytial virus monoclonal antibody, reduces hospitalization from respiratory syncytial virus infection in high-risk infants. Pediatrics 1998;102(3):531-537. https://doi.org/10.1542/peds.102.3.531
234. Morris SK, Dzolganovski B, Beyene J, Sung L. A meta-analysis of the effect of antibody therapy for the prevention of severe respiratory syncytial virus infection. BMC Infect Dis 2009;9:106-106. https://doi.org/10.1186/1471-2334-9-106
235. Green RJ, Zar HJ, Jeena PM, Madhi SA, Lewis H. South African guideline for the diagnosis, management and prevention of acute viral bronchiolitis in children. S Afr Med J 2010;100(5):320-325. https://doi.org/10.7196/samj.4016
236. Zar HJ, White DA, Morrow B, et al. The bronchiolitis season is upon us - recommendations for the management and prevention of acute viral bronchiolitis. S Afr Med J 2015;105(7):525-526. https://doi.org/10.7196/SAMJnew.8040
237. World Health Organization. Vaccines against influenza WHO position paper – November 2012. Wkly Epidemiol Rec 2012;87(47):461-476.
238. Evans C, Jones CE, Prendergast AJ. HIV-exposed, uninfected infants: New global challenges in the era of paediatric HIV elimination. Lancet Infect Dis 2016;16(6):e92-e107. https://doi.org/10.1016/S1473-3099(16)00055-4
239. Slogrove AL, Frigati L, Gray DM. Maternal HIV and paediatric lung health. Paediatr Respir Rev 2017;21:47-53. https://doi.org/10.1016/j.prrv.2016.08.004
240. Rabie H, Goussard P. Tuberculosis and pneumonia in HIV-infected children: An overview. Pneumonia 2016;8:19. https://doi.org/10.1186/s41479-016-0021-y
241. Brady MT, Oleske JM, Williams PL, et al. Declines in mortality rates and changes in causes of death in HIV-1-infected children during the HAART era. J Acquir Imm Def Syndr 2010;53(1):86-94. https://doi.org/10.1097/QAI.0b013e3181b9869f
242. Steenhoff AP, Josephs JS, Rutstein RM, et al. Incidence of and risk factors for community acquired pneumonia in US HIV-infected children, 2000-2005. AIDS 2011;25(5):717-720. https://doi.org/10.1097/QAD.0b013e3283440583
243. Gray DM, Zar HJ. Community-acquired pneumonia in HIV-infected children: A global perspective. Curr Opin Pulm Med 2010;16(3):208-216. https://doi.org/10.1097/MCP.0b013e3283387984
244. Hesseling AC, Cotton MF, Jennings T, et al. High incidence of tuberculosis among HIV-infected infants: Evidence from a South African population-based study highlights the need for improved tuberculosis control strategies. Clin Infect Dis 2009;48(1):108-114. https://doi.org/10.1086/595012
245. Rabie H, Frigati L, Hesseling AC, Garcia-Prats AJ. Tuberculosis: Opportunities and challenges for the 90-90-90 targets in HIV-infected children. J Int AIDS Soc 2015;18(Suppl 6):20236. https://doi.org/10.7448/IAS.18.7.20236
246. Punpanich W, Groome M, Muhe L, Qazi SA, Madhi SA. Systematic review on the etiology and antibiotic treatment of pneumonia in human immunodeficiency virus-infected children. Pediatr Infect Dis J 2011;30(10):e192-e202. https://doi.org/10.1097/INF.0b013e31822d989c
247. Sherman GG, Mazanderani AH, Barron P, et al. Toward elimination of mother-to-child transmission of HIV in South Africa: How best to monitor early infant infections within the prevention of mother-to-child transmission program. J Glob Health 2017;7(1):010701. https://doi.org/10.7189/jogh.07.010701
248. Johnson LF, Stinson K, Newell ML, et al. The contribution of maternal HIV seroconversion during late pregnancy and breastfeeding to mother-to-child transmission of HIV. J Acquir Immune Defic Syndr 2012;59(4):417-425. https://doi.org/10.1097/QAI.0b013e3182432f27
249. Dinh TH, Delaney KP, Goga A, et al. Impact of maternal HIV seroconversion during pregnancy on early mother to child transmission of HIV (MTCT) measured at 4 - 8 weeks postpartum in South Africa 2011 - 2012: A national population-based evaluation. PLoS ONE 2015;10(5):e0125525. https://doi.org/10.1371/journal.pone.0125525
250. Dinh TH, Mushavi A, Shiraishi RW, et al. Impact of timing of antiretroviral treatment and birth weight on mother-to-child human immunodeficiency virus transmission: Findings from an 18-month prospective cohort of a nationally representative sample of mother-infant pairs during the transition from option A to option B+ in Zimbabwe. Clin Infect Dis 2018;66(4):576-585. https://doi.org/10.1093/cid/cix820
251. Tippett Barr BA, van Lettow M, van Oosterhout JJ, et al. National estimates and risk factors associated with early mother-to-child transmission of HIV after implementation of option B+: A cross-sectional analysis. Lancet HIV 2018;5(12):e688-e695. https://doi.org/10.1016/s2352-3018(18)30316-3
252. Avino LJ, Naylor SM, Roecker AM. Pneumocystis jirovecii pneumonia in the non-HIV-infected population. Ann Pharmacother 2016;50(8):673-679. https://doi.org/10.1177/1060028016650107
253. Chintu C, Bhat GJ, Walker AS, et al. Co-trimoxazole as prophylaxis against opportunistic infections in HIV-infected Zambian children (CHAP): A double-blind randomised placebo-controlled trial. Lancet 2004;364(9448):1865-1871. https://doi.org/10.1016/S0140-6736(04)17442-4
254. Terblanche AJ, Green RJ, Rheeder P, Wittenberg DF. Adjunctive corticosteroid treatment of clinical Pneumocystis jiroveci pneumonia in infants less than 18 months of age – a randomised controlled trial. S Afr Med J 2008;98(4):287-290.
255. Newberry L, O'Hare B, Kennedy N, et al. Early use of corticosteroids in infants with a clinical diagnosis of Pneumocystis jiroveci pneumonia in Malawi: A double-blind, randomised clinical trial. Paediatr Int Child Health 2017;37(2):121-128. https://doi.org/10.1080/20469047.2016.1260891
256. Kitchin OP, Wessels F, Masekela R, Becker P, Green RJ. Costs of admission for paediatric pneumonia in a setting of human immunodeficiency virus infection. Int J Tuberc Lung Dis 2011;15(12):1702-1707. https://doi.org/10.5588/ijtld.11.0167
257. Hsiao NY, Zampoli M, Morrow B, Zar HJ, Hardie D. Cytomegalovirus viraemia in HIV exposed and infected infants: Prevalence and clinical utility for diagnosing CMV pneumonia. J Clin Virol 2013;58(1):74-78. https://doi.org/10.1016/j.jcv.2013.05.002
258. Murphy CG, van de Pol AC, Harper MB, Bachur RG. Clinical predictors of occult pneumonia in the febrile child. Acad Emerg Med 2007;14(3):243-249. https://doi.org/10.1197/j.aem.2006.08.022
259. Rutman MS, Bachur R, Harper MB. Radiographic pneumonia in young, highly febrile children with leukocytosis before and after universal conjugate pneumococcal vaccination. Pediatr Emerg Care 2009;25(1):1-7. https://doi.org/10.1097/PEC.0b013e318191dab2
260. World Health Organization. Oxygen Therpy for Children: A Manual for Health Workers. Geneva: WHO, 2016.
261. Kneyber MCJ, de Luca D, Calderini E, et al. Recommendations for mechanical ventilation of critically ill children from the Paediatric Mechanical Ventilation Consensus Conference (PEMVECC). Intens Care Med 2017;43(12):1764-1780. https://doi.org/10.1007/s00134-017-4920-z
