# Diagnosis and management of community-acquired pneumonia in children: South African Thoracic Society guidelines

**DOI:** 10.7196/AJTCCM.2020.v26i3.104

**Published:** 2020-10-13

**Authors:** H J Zar, D P Moore, S Andronikou, A C Argent, T Avenant, C Cohen, R J Green, G Itzikowitz, P Jeena, R Masekela, M P Nicol, A Pillay, G Reubenson, S A Madhi

**Affiliations:** 1 Department of Paediatrics and Child Health, Red Cross War Memorial Children’s Hospital and Faculty of Health Sciences, University of Cape Town, South Africa; 2 South African Medical Research Council Unit on Child and Adolescent Health, University of Cape Town, South Africa; 3 Department of Paediatrics and Child Health, Chris Hani Baragwanath Academic Hospital, and Faculty of Health Sciences, University of the Witwatersrand, Johannesburg, South Africa; 4 Department of Pediatric Radiology, Perelman School of Medicine, University of Philadephia, USA; 5 Department of Paediatrics and Child Health, Faculty of Health Sciences, University of Pretoria, South Africa; 6 Centre for Respiratory Diseases and Meningitis, National Institute for Communicable Diseases, Johannesburg, South Africa; 7 Department of Paediatrics and Child Health, Nelson R Mandela School of Medicine, School of Clinical Medicine, College of Health Sciences, University of KwaZulu-Natal, Durban, South Africa; 8 Division of Medical Microbiology, Department of Pathology, Faculty of Health Sciences, University of Cape Town, South Africa; and Division of Infection and Immunity, School of Biomedical Sciences, University of Western Australia, Perth, Australia; 9 Department of Paediatrics and Child Health, Rahima Moosa Mother and Child Hospital, Faculty of Health Sciences, University of the Witwatersrand, Johannesburg, South Africa; 10 South African Medical Research Council Vaccine and Infectious Diseases Analytics Unit, University of the Witwatersrand, Johannesburg, South Africa; 11 Department of Science and Technology/National Research Foundation: South African Research Chair in Vaccine Preventable Diseases, Faculty of Health Sciences, University of the Witwatersrand, Johannesburg, South Africa

**Keywords:** pneumonia, childhood, guideline, aetiology, prevention, treatment

## Abstract

**Background:**

Pneumonia remains a major cause of morbidity and mortality amongst South African children. More comprehensive immunisation regimens, strengthening of HIV programmes, improvement in socioeconomic conditions and new preventive strategies have impacted on the epidemiology of pneumonia. Furthermore, sensitive diagnostic tests and better sampling methods in young children improve aetiological diagnosis.

**Objectives:**

To produce revised guidelines for pneumonia in South African children under 5 years of age.

**Methods:**

The Paediatric Assembly of the South African Thoracic Society and the National Institute for Communicable Diseases established seven expert subgroups to revise existing South African guidelines focusing on: (i) epidemiology; (ii) aetiology; (iii) diagnosis; (iv) antibiotic management and supportive therapy; (v) management in intensive care; (vi) prevention; and (vii) considerations in HIV-infected or HIVexposed, uninfected (HEU) children. Each subgroup reviewed the published evidence in their area; in the absence of evidence, expert opinion was accepted. Evidence was graded using the British Thoracic Society (BTS) grading system. Sections were synthesized into an overall guideline which underwent peer review and revision.

**Recommendations:**

Recommendations include a diagnostic approach, investigations, management and preventive strategies. Specific recommendations for HIV infected and HEU children are provided.

**Validation:**

The guideline is based on available published evidence supplemented by the consensus opinion of SA paediatric experts. Recommendations are consistent with those in published international guidelines.

## Background

Community-acquired pneumonia forms part of a broad spectrum of acute lower respiratory tract illness (LRTI) in children. This terminology recognises that LRTI is a spectrum of illness ranging from airway to parenchymal disease, dependent on the pathogen/s and the host response.^[1]^ As prevention and management strategies for childhood pneumonia have been strengthened, so reductions in pneumonia incidence, severity and shifts in aetiology have occurred. Besides the impact on under-5 mortality, pneumonia in early childhood may reduce lung function, setting a trajectory for long-term impairment of lung health including the development of asthma or chronic obstructive pulmonary disease (COPD) in adulthood.

In South Africa (SA), with socioeconomic improvements, reduction in perinatal HIV transmission, increasing numbers of HIV-exposed uninfected (HEU) children, effective combination antiretroviral therapy (ART) programmes, and improved immunisation, the epidemiology and aetiology of childhood pneumonia is changing.

In addition, improved diagnostic methods have highlighted the importance of multiple pathogens contributing to co-infection in the aetiology of respiratory illness, and the importance of organism interactions. Current treatment and preventive strategies for childhood pneumonia therefore require revision.

## Epidemiology

Pneumonia is one of the most common causes of morbidity and mortality in SA children, despite improvements in immunisation and HIV management programmes. In 2017 there were ~320 000 pneumonia episodes and 4 100 deaths in SA children aged <5 years.^[2]^ The incidence of pneumonia in children <5 years old in SA has declined by ~50% from 2000 - 2015, including an estimated 71% reduction in children living with HIV (CLWH).^[3]^ The rollout of interventions to prevent mother-to-child transmission (PMTCT) of HIV and increased provision of ART to HIV-infected individuals has contributed to the decline in pneumonia cases.^[4]^ Furthermore, following introduction of pneumococcal conjugate vaccine (PCV) into the SA public immunisation programme in 2009, PCV immunisation was estimated to have reduced under-5 hospitalisations for all-cause pneumonia by 33% and 39% in HIV-uninfected and HIV-infected children, respectively, by 2014.^[5]^ Invasive pneumococcal disease has also declined by 69% among children <2 years, including an 89% reduction in PCV7 serotypes and a 57% reduction in PCV13 serotypes in 2012.^[6]^ Despite these reductions, pneumonia remains one of the most important causes of death in SA children <5 years and is a major cause of healthcare utilisation and morbidity.^[7,8]^ There are several determinants of pneumonia severity and mortality. The case fatality risk (CFR) among children hospitalised with pneumonia in 5 SA hospitals was 2% from 2009 - 2012,^[8,9]^ with HIV infection an important risk factor for hospitalisation and in-hospital mortality.^[10]^ HEU infants have an increased risk of hospitalisation compared to HIV-unexposed infants and an increased risk of in-hospital death, predominantly in the first 6 months of life.^[11,12]^ Increased risk of hospitalisation in HEU infants has also been found for specific pathogens such as pneumococcus, respiratory syncytial virus (RSV) and influenza virus.^[10,11,13]^ Additional risk factors for severe pneumonia include infancy (particularly those <4 months), premature birth, incomplete immunisation, maternal smoking or household tobacco smoke exposure, indoor air pollution, low birthweight, malnutrition, lack of exclusive breastfeeding and overcrowding.^[9,14,15]^

## Summary – epidemiology

1. In SA, pneumonia incidence in children has declined by around 50% from 2000 to 2015, with an estimated 70% reduction in episodes in HIV-infected children.
2. A reduction in vertical transmission of HIV, increased provision of ART, and PCV have contributed to this reduction.
3. Pneumonia remains a major cause of morbidity and death in SA children.
4. Risk factors for pneumonia hospitalisation include: HIV infection, HIV exposure (predominantly in infants), young age (particularly those <4 months), premature birth, incomplete immunisation, maternal smoking or household tobacco smoke exposure, indoor air pollution, low birthweight, malnutrition, non-exclusive breastfeeding and overcrowding.

## Diagnosis

### Clinical diagnosis

**Clinical presentation**

The main symptoms of pneumonia are cough, difficulty breathing or tachypnoea. Physical examination should include: assessment of the child’s general appearance, measurement of respiratory rate, evaluation of the work of breathing and pulse oximetry. Auscultation of the chest should be done where possible (evidence level II); however, there is wide inter- and intra-observer variability in the interpretation of auscultatory sounds in paediatric pneumonia.^[16]^

**Fig. 1:**
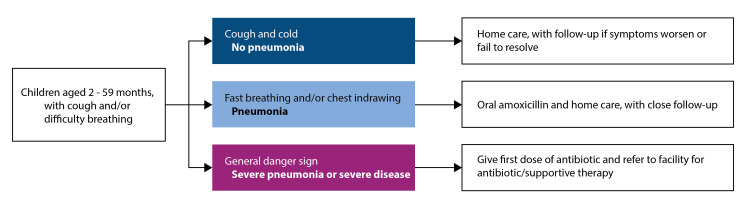
Revised World Health Organization classification and treatment of childhood pneumonia at health facilities.^[2]^

The World Health Organization (WHO) guidelines classify children with cough or difficulty breathing into three categories, based on clinical signs – severe pneumonia, pneumonia or no pneumonia (evidence level Ia) (Fig. 1; Table 1).^[17]^ Children with lower chest indrawing are now classified as having pneumonia, rather than severe pneumonia. Treatment is based on these categories – severe pneumonia requires referral to hospital and antibiotics; pneumonia requires oral antibiotics and outpatient management with follow-up; and no pneumonia is treated symptomatically. However, children living with HIV (CLWH), malnourished children or immunocompromised children, who present with lower chest indrawing, should be regarded as having severe pneumonia and referred to hospital for appropriate management (evidence level Ib).

**Assessment of severity**

Assessment of the **general appearance** of the child is helpful to evaluate severity of illness. Any child with a general danger sign requires referral to hospital. All children <2 months of age with signs of pneumonia require hospital admission (Table 2).

Excessive **work of breathing**, as indicated by grunting, nasal flaring or very severe chest wall indrawing, is a useful indicator of severity (evidence level Ia).^[18,19]^ British Thoracic Society (BTS) guidelines recommend that signs indicating excessive work of breathing are more specific for diagnosing severe pneumonia than respiratory rate (evidence level II).^[20]^

Assessment of **oxygenation** is important as a measure of severity (evidence level Ia).^[21]^ Pulse oximetry should be performed in all children, using a paediatric wrap-around probe (evidence level Ib). A saturation of <92% or <90% at higher altitudes (≥1 800 m) indicates the need for hospital admission and supplemental oxygen (evidence level Ia).^[20,22]^

### Radiological diagnosis

A chest X-ray (CXR) may be useful for confirming the presence of pneumonia or complications such as a lung abscess or empyema (Table 3). A CXR cannot accurately discriminate between viral and bacterial pneumonia (evidence level II).^[20,22,23]^ Overall, a CXR does not influence outcome and rarely informs changes of treatment in the ambulatory setting (evidence level Ib).^[23]^ There is also no evidence that a lateral CXR improves the diagnostic yield in children with pneumonia,^[24]^ except for detection of hilar adenopathy if tuberculosis (TB) is suspected (evidence level II).^[25,26]^

The use of a CXR has several limitations, including radiographic features being masked by anatomical structures; a normal CXR in the early stages of pneumonia; and lack of inter-reader agreement in interpretation.^[27]^ Clinician-led point-of-care ultrasound is increasingly being used, with higher inter-observer agreement than for a CXR (evidence level Ib).^[27,28]^ Evidence suggests a similar or higher yield in the diagnosis of consolidation or pleural effusion when using ultrasound (evidence level Ib). However, ultrasound is not yet routinely available for the diagnosis of pneumonia, and a CXR remains the standard investigation.^[12]^

Computed tomography (CT) is not recommended as a first-line diagnostic tool, but where available, can be considered for detecting complications of pneumonia in the acute or subacute phase (for diagnosing a suppurative complication such as necrotising pneumonia, abscess or empyema) and in the chronic phase (for diagnosing bronchopleural fistula or detecting and localising bronchiectasis); it can also be useful for differentiating pneumonia from other pathological conditions, including endobronchial lesions/foreign bodies causing atelectasis and for demonstrating previously undiagnosed, underlying congenital lesions.^[20,22]^ Radiation dose is less of a concern, as low-dose scans (at doses of ~10 CXRs or 3 - 5 anteroposterior (AP) and lateral CXRs) can be performed.

**Follow-up chest X-ray**

A follow-up CXR after acute uncomplicated pneumonia is not indicated if there is clinical improvement (evidence level II).^[22,29]^ A follow-up CXR at 2 - 4 weeks should be done:

- in children with lobar collapse;
- to document resolution of a round pneumonia (as this may mimic the appearance of a Ghon focus);
- and in those with ongoing respiratory symptoms.^[20,22]^

A chest ultrasound scan should be considered as an alternative to a repeat CXR in children with unresolving or worsening signs and symptoms to detect complications such as pleural effusion (evidence level Ib).^[28,30,31]^

### Aetiological diagnosis

Clinical assessment and chest radiography cannot determine the aetiology of pneumonia.^[20,32-34]^ Diffuse bilateral wheezing is, however, often associated with a viral infection, especially respiratory syncytial virus (RSV) (evidence level Ib).^[33]^ Various diagnostic modalities are available for aetiological diagnosis, such as microscopy, molecular diagnostics, culture and antigen detection (Table 4).

**Table 4:** Summary of investigations in children hospitalised for pneumonia^[12]^

	Advantages	Disadvantages
**Vital signs**		
Pulse oximetry	Accurate measure of hypoxaemia; guides the use of supplemental oxygen	
****Radiological tests****		
Chest X-ray	Assess extent of pneumonia.	Unable to distinguish aetiology.
	Detect complications.	Poor intra- and inter-observer agreement for interpretation of some features.
Lung ultrasound	Higher inter- and intrapersonal agreement of radiological findings compared with CXR. May be more sensitive than CXR for detecting abnormalities. Easily repeatable, no radiation. Can be done by non-radiologists with minimal training.	Not widely available Few clinicians have expertise in its use
**Blood**		
Culture for	Relative ease of collection.	Low sensitivity; therefore,
bacterial pathogens	Positive culture with a clinically	high cost per case detected
	significant pathogen has high specificity.	
	Able to guide empirical antibiotic susceptibility patterns.	
Molecular testing	More sensitive than blood culture for some targets, e.g. pneumococcal lytA. Useful for CMV viral load.	Lacks specificity for disease, e.g. lytA detection may reflect pneumococcal carriage
Serology	Useful for epidemiological studies and for specific pathogens, e.g. B. pertussi	Usually requires acute and convalescent sera; therefore, not useful for guiding acute treatment decisions
Biomarker detection	Potential to discriminate bacterial vs. viral infection	Accuracy for distinguishing bacterial vs. viral pneumonia is suboptimal for available biomarkers (CRP, ESR and PCT)
HIV infection	HIV testing essential in hospitalised children whose HIV status is unknown.	
	HIV infection or HIV exposure may impact on the spectrum of pathogens considered in empirical antibiotic therapy.	
**Nasopharyngeal or nasal swab** **or aspirate**		
Bacterial culture, molecular or antigen detection of bacteria and viruses	Ease of collection, relatively good correlation of results with sputum testing, method of choice for some viruses (e.g. RSV, influenza, para-influenza virus, SARS-CoV-2), bacteria (*B. pertussis*) and *P. jirovecii*	Colonisation or infection of the upper airway does not imply that organisms are causing pneumonia. Predictive value of attributing causality is high for RSV, influenza virus, para-influenza virus 3 and *M. tuberculosis*.
		Limited value for most other bacteria and viruses
**Sputum** **(expectorated or induced)**		
Bacterial culture, molecular or antigen detection of bacteria (*M. tuberculosis, B. pertussis*) or *P. jirovecii*	Relative ease of collection. Incremental yield over testing of upper respiratory samples for *M. tuberculosis, B. pertussis* and *P. jirovecii*	Requires expertise, and should be conducted in a dedicated space that is well ventilated.May also detect organisms colonising or infecting upper airway
**Urine antigen testing**		
Antigen detection	Relative ease of collection	Poor specificity for pneumococcal disease in children due to high prevalence of nasopharyngeal carriage
**Tracheal aspiration or bronchoalveolar lavage**		
Bacterial culture, molecular or antigen detection of bacteria, *P. jirovecii* and viruses	More representative of organisms in the lower respiratory tract. Less likely to be contaminated by upper respiratory tract flora	Few comparative studies vs. other sample types. Costly, invasive, requires expertise
**Percutaneous lung aspiration**		
Bacterial culture, molecular or antigen detection of bacteria and viruses	Most representative of lower respiratory tract, least contamination with upper airway respiratory tract flora	Useful mainly for peripheral infective foci in the right lung. Invasive, and requires expertise. Small risk of serious complications.

CXR = chest X-ray

CMV = cytomegalovirus

*B. pertussis* = *Bordetella pertussis*

CRP = C-reactive protein

ESR = erythrocyte sedimentation rate

PCT = procalcitonin

RSV = respiratory syncytial virus

*P. jirovecii* = *Pneumocystis jirovecii*

*M. tuberculosis* = *Mycobacterium tuberculosis*

Recent advances in understanding the aetiology have highlighted that pneumonia may be due to interactions or co-infection with several organisms, including viral-viral and viral-bacterial infections.^[22,35]^ Testing of upper respiratory samples may not, however, discriminate between colonising and pathogenic organisms, making it difficult to attribute aetiology. Identification of organisms such as *Bordetella pertussis*, RSV, influenza virus, parainfluenza virus, severe acute respiratory syndrome coronavirus 2 (SARS-CoV-2) in children with clinical and/or radiological features of pneumonia, or *Mycobacterium tuberculosis* in upper respiratory samples among children is, however, strongly attributable to the aetiology of lower respiratory tract infection (evidence level Ia).^[36,37]^

**The following should be considered when investigating the aetiology of lower respiratory tract infection:**

- General tests for infection, including acute-phase reactants (erythrocyte sedimentation rate (ESR), C-reactive protein (CRP), white cell count (WCC), neutrophil count and procalcitonin (PCT)) do not reliably differentiate bacterial from viral pneumonia and should not be routinely used (evidence level Ib).^[22,38]^ CRP concentrations ≥40 mg/L with radiological confirmation of pneumonia suggests bacterial pneumonia (evidence level II)^[39]^
- HIV status should be determined in all children requiring hospital admission for pneumonia
- Microbiological investigations on blood, pleural fluid or respiratory samples should only be done in children requiring hospital admission (i.e. in those with severe disease or complications or in outbreak situations) (evidence level IVa)^[20,22]^
- For detection of viruses, polymerase chain reaction (PCR) and/or immunofluorescence on nasal samples may be useful (evidence level Ib). Viruses strongly associated with pneumonia include RSV, influenza and parainfluenza virus and SARS-CoV-2 virus in symptomatic children. Detection of adenovirus, human metapneumovirus (HMPV) or rhinovirus, even though associated with pneumonia, should be interpreted with caution, as healthy children or those with upper respiratory tract infection (URTI) may have a positive test (evidence level Ia).^[35,40]^ Testing for SARS-CoV-2 using PCR can be done on mid-tubinate nasal swabs
- Blood culture has a very low diagnostic yield. Antibiotic pre-exposure and specimen volume impact on blood culture yield.^[41]^ Overall, ~5% of blood cultures of suspected bacterial pneumonia cases are positive; the yield is higher in severe pneumonia^[42,43]^ and in CLWH^[44]^
- Induced sputum provides a higher yield than upper respiratory secretions for *B. pertussis*, *Pneumocystis jirovecii* and *M. tuberculosis*^[45,49]^
- Pulmonary TB should be considered in a child presenting with severe pneumonia or penumonia with a known TB contact, if the tuberculin skin test is positive, if the child is malnourished or has lost weight, and in CLWH or in those who are HIV-exposed (evidence level 1b).^[50,52]^ Two sequential respiratory samples, preferably expectorated or induced sputum, should be tested with Xpert MTB/RIF Ultra (Cepheid, USA) and mycobacterial culture with drug susceptibility testing (evidence level Ib)^[53]^
- Pleural fluid can be tested for microscopy, culture, pneumococcal antigen (by latex agglutination), PCR for bacteria, mycobacterial culture and Xpert MTB/RIF Ultra (evidence level II).

### Section summary: Diagnosis

1. A diagnosis of pneumonia should be considered in any child who has an acute onset of cough, fast breathing or difficulty breathing.
2. Revised WHO guidelines classify children with cough or difficulty breathing into:
  - severe pneumonia
  - pneumonia
  - no pneumonia
  Malnourished or immunocompromised children with lower chest indrawing should be managed as severe pneumonia (evidence level Ib).
3. Excessive work of breathing, as indicated by grunting, nasal flaring or severe chest wall indrawing, is an important indicator of severity (evidence level Ia).
4. Pulse oximetry should be performed on all children, with referral to hospital for oxygen if saturation is <92% at sea level or <90% at an altitude >1 800 m (evidence level Ia).
5. A CXR should not be done routinely (evidence level Ib), but should be performed in severe cases to confirm pneumonia and detect complications or when TB is suspected.
6. A follow-up CXR should only be done if the condition of a child does not improve or complications are suspected (evidence level II).
7. Evidence for point-of-care chest ultrasound for diagnosis is accumulating. A chest ultrasound scan, rather than a repeat CXR, should be considered in children with ongoing symptoms (evidence level II).
8. CRP ≥40 mg/L with radiological confirmation of pneumonia is supportive of a bacterial aetiology (evidence level II).
9. Microbiological investigations should not be performed routinely on children, but only in those requiring hospitalisation or in outbreak settings (evidence level IVa).
10. In children with pneumonia, testing of nasal samples with PCR is useful for detecting RSV, influenza virus, parainfluenza virus or SARS-CoV-2; other viruses should be cautiously interpreted, as healthy children or those with URTI may have a positive test.
11. Induced sputum may provide a higher yield than upper respiratory samples for *B. pertussis*, *P. jirovecii* and *M. tuberculosis*.
12. Investigations for TB should be done in children with severe pneumonia or pneumonia with a history of a TB contact, a positive tuberculin skin test, loss of weight or malnutrition or if HIV-infected.

### Aetiology diagnosis

A wide spectrum of pathogens, ranging from viral and bacterial to fungal and parasitic organisms, is implicated in pneumonia pathogenesis, and disease may be due to multiple organisms (evidence level Ia). If children are hospitalised with pneumonia, bacterial-viral co-infection is often the norm (evidence level Ib).^[54,55]^ In the current era of conjugate vaccines targeting *Haemophilus influenzae* type b (Hib) and *Streptococcus pneumoniae*, there has been a shift in the spectrum of pathogens causing pneumonia, with viruses contributing to a greater proportion of severe LRTI episodes (evidence level Ib) and non-typable *H. influenzae* and *S. aureus* emerging as important bacterial pathogens.^[48,49,56-60]^

**Bacterial organisms**

The Drakenstein Child Health Study and the Pneumonia Etiology Research for Child Health (PERCH) case-control studies indicate that non-typable *H. influenzae* and *S. aureus* are now the leading bacterial causes of severe pneumonia in SA children (Supplementary Table 1 - http://ajtccm.org.za/public/sup/104.pdf) (evidence level Ib).^[48,61,62]^

Children presenting with empyema or pleural effusion, confluent dense consolidation or pneumatocoele on CXR associated with high-grade pyrexia and elevation of acute phase reactants (e.g. CRP), or those with features of suppurative lung disease, are highly likely to have a bacterial cause (evidence level Ib).^[63,64]^ The most frequently isolated bacteria are *S. aureus*, non-typable *H. influenzae*, *S. pneumoniae* and *Streptococcus pyogenes* (Group A *Streptococcus*).^[63,65]^

Gram-negative enteric organisms, particularly *Klebsiella pneumoniae*, *Escherichia coli*, *Enterobacter cloacae* and *Salmonella* spp. are important pneumonia pathogens in HIV-infected or malnourished children in sub-Saharan Africa (evidence level Ib).^[44,66,67]^ In the Child Health and Mortality Prevention Surveillance (CHAMPS) Program, a study designed to establish the infectious aetiology of fatal illness in children dying in hospital, *K. pneumoniae* was identified in 16% of community-acquired pneumonia deaths, and the most common organism isolated in death from hospital-acquired infection in Soweto.^[68]^ Non-fermenting Gram-negatives, such as *Moraxella catarrhalis* or *Pseudomonas aeruginosa* are increasingly recognised as contributors to bacterial pneumonia in children (evidence level II).

*Bordetella pertussis* is an important cause of pneumonia in young infants who are unimmunised or incompletely immunised. In Cape Town, *B. pertussis* prevalence among young children hospitalised with pneumonia was 16% in CLWH, 11% in HEU and 5% in HIV-unexposed children.^[47]^ In the PERCH study, *B. pertussis* was detected in 2% of HIVuninfected and 1% of HIV-infected children hospitalised with severe or very severe pneumonia.^[61,62]^

**Tuberculosis**

*M. tuberculosis* is increasingly recognised as an important pathogen in acute pneumonia in settings with a high burden of tuberculosis and HIV (evidence level Ib). In SA, 43 - 85% of children with culture-confirmed tuberculosis presented with acute cough (<14 days) (evidence level Ib).^[50,67,69,70]^ In the Drakenstein study, the incidence of tuberculin skin test (TST) conversion was 12 per 100 child years, and that of pulmonary tuberculosis 2.9 per 100 child years (evidence level Ib).^[71]^ In the PERCH study, 3% of SA children hospitalised with WHO-defined severe pneumonia had microbiologically confirmed tuberculosis (evidence level Ib).^[53]^ Given the suboptimal sensitivity of mycobacterial culture, the proportion of SA paediatric pneumonia cases associated with *M. tuberculosis* is likely to be ~7 - 15% (evidence level IVa).^[61,62,71]^

If pulmonary tuberculosis is diagnosed, the child must be notified and treatment should be initiated in accordance with the South African Guidelines for the Management of Tuberculosis in Children (2013; https://health-e.org.za/wp-content/uploads/2014/06/National-Childhood-TB-Guidelines-2013.pdf)

### Respiratory viruses

Respiratory viruses are the leading cause of pneumonia and of hospitalisation in young children (evidence level Ia).^[9,58,72]^ Respiratory virus-related illness follows predictable seasonal shifts; in SA this season is between March and September (autumn to early spring) (evidence level II).^[73,74]^ Respiratory syncytial virus causes 18 - 31% of pneumonia episodes (evidence level Ia).^[35]^ Other viruses associated with pneumonia include influenza, parainfluenza, HMPV, human rhinovirus and adenovirus (Supplementary Table 2 - http://ajtccm.org.za/public/sup/104.pdf) (evidence level Ib).^[9,48,49]^

Respiratory viruses are diverse, continually adapting and prone to causing intermittent epidemics and pandemics, particularly when adapting from animal reservoirs to the human host. Numerous pandemic influenza events occurred in the 20th century,^[75]^ and the pandemic 2009 H1N1 influenza strain was implicated in the first influenza pandemic of the 21st century. Coronaviruses, too, have been associated with epidemics in recent decades.^[76–79]^ Highly virulent influenza and coronavirus outbreaks may be associated with considerable case fatality risk, and it is for this reason that all cases of severe acute respiratory syndrome in which no alternative aetiological diagnosis is made be investigated for novel respiratory viruses. Such cases constitute a Category 1 Notifiable Medical Condition in SA (https://www.nicd.ac.za/wp-content/uploads/2017/06/NMC-list_2018.pdf).

Although novel coronavirus disease 2019 (COVID-19) appears to affect children mildy, with most developing asymptomatic or mild illness,^[80]^ children with acute lower respiratory illness requiring hospitalisation should be tested for SARS-CoV-2 through the epidemic. Evidence on COVID-19 in children is rapidly evolving and current management of paediatric cases should defer to recommendations from the National Department of Health (https://www.nicd.ac.za/diseases-a-z-index/covid-19/covid-19-guidelines/). Children infected with SARS-CoV-2 may present with non-respiratory illness including multisystem inflammatory disease; and so, where resources permit, hospitalised children may be screened for infection, especially if there is a history of contact with a positive adult.

Viral-viral co-infections and viral-bacterial co-infections are increasingly recognised in the pathogenesis of severe pneumonia (evidence level Ib).^[81]^ Preceding respiratory viral infection may prime the respiratory tract for new acquisition of bacterial colonisation, and increase in density of colonisation or vice versa (evidence level Ib).^[33,82,83]^

Measles and varicella-zoster virus occasionally cause severe, or fatal, pneumonia in the context of outbreaks or epidemics (evidence level II). Typically, malnourished or immunocompromised children tend to develop the most severe forms of pneumonia related to these organisms.^[84,85]^ Children hospitalised with measles or varicella-zoster virus-associated pneumonia frequently develop superimposed bacterial pneumonia (primarily *S. aureus* and *S. pyogenes*) and require treatment with broad-spectrum antibiotic therapy (evidence level IVa).^[86–88]^

**Opportunistic organisms**

*Pneumocystis jirovecii*, either alone or as a co-pathogen with cytomegalovirus (CMV), is a leading infection in CLWH not on ART in SA (evidence level Ib).^[62,89-92]^ Typically, pneumocystis pneumonia (PCP) affects young infants, between 6 weeks and 6 months of age; presentation is with severe hypoxia, dyspnoea, low-grade fever and normal chest auscultation (evidence level Ib).^[93]^ However, *P. jirovecii* also commonly colonises the airways in infants.

The association of CMV alone with severe childhood pneumonia in HIV-infected and HEU children is contentious (evidence level II),^[94,95]^ although histological evidence of CMV pneumonia is frequently seen in HIV-infected children dying of pneumonia.^[96]^ Fatal disseminated disease associated with CMV is also well described in children who are solid organ or bone marrow transplant recipients.^[97,98]^

**Atypical bacteria**

*Chlamydophila pneumoniae*, **Chlamydia trachomatis**, *Mycoplasma pneumoniae* and *Legionella* spp. are infrequently associated with pneumonia in SA children (evidence level II),^[48,61,62,99]^ but should be considered in the differential diagnosis in children with progressive respiratory failure, despite broad-spectrum antibiotic cover (evidence level IVa). Age <5 years and HIVinfection were associated with hospitalisation for severe M. pneumoniae pneumonia in SA.^[100]^

### Summary – aetiology

1. Co-infections are common in pneumonia pathogenesis.
2. The most frequently isolated bacterial pathogens following PCV and Hib immunisation are non-typable *H. influenzae* and *S. aureus*.
3. *B. pertussis* is implicated in severe pneumonia aetiology in those with incomplete immunisation.
4. *M. tuberculosis* is an important pathogen in settings with a high burden of tuberculosis.
5. Gram-negative bacteria are important pathogens in HIV-infected and malnourished children.
6. Respiratory viruses, particularly RSV, are responsible for most pneumonia episodes. Influenza, parainfluenza, adenovirus and human bocavirus, HMPV and rhinovirus are common, although not always pathogenic.
7. In HIV-infected children not on ART, *P. jirovecii* either alone or as a co-pathogen with CMV, is an important opportunistic infection.

## Treatment of childhood pneumonia

### Antibiotic treatment

Choice of empiric antibiotic treatment depends on the child’s age, possible aetiology, antimicrobial resistance patterns, previous treatment, as well as factors affecting host susceptibility, including HIV, nutritional and vaccination status. All children with signs of pneumonia or severe pneumonia should receive antibiotics (evidence level Ib).^[17,101]^

**Adaptation of guidance to address antibiotic resistance**

Substantial (>80%) reductions in the incidence of invasive pneumococcal disease were observed within 4 years of pneumococcal conjugate vaccine (PCV) introduction.^[3]^ High-dose amoxicillin is effective against pneumococci with low- and intermediate-level penicillin non-susceptibility causing pneumonia. Empiric therapy for hospitalised children with community-acquired pneumonia (CAP) should cover non-typable *H. influenzae* and *S. aureus*. ^[61,62]^

**Which empiric antibiotic?**

For severe pneumonia in children >1 month of age, amoxicillin clavulanate (90 mg/kg/day of amoxicillin component) is recommended. Oral therapy (45 mg/kg/day 12-hourly of amoxicillin component) is preferable, but intravenous therapy (30 mg/kg/dose of intravemous amoxicillin component 8-hourly) should be initiated in children who are unable to tolerate oral medications, if there is a concern about oral absorption, or if the child is severely ill.

For children <1 month of age, initial therapy should be intravenous ampicillin (40 mg/kg/dose 6-hourly) and gentamicin (7.5 mg/kg/ dose daily) to cover common neonatal pathogens, including Listeria spp. (evidence level II). Group B Streptococcus, *S. aureus*, *Chlamydia trachomatis* and viruses should also be considered as causes of neonatal pneumonia. Consideration should be given to broadening cover if there is no clinical improvement within 48 hours of initiation of therapy

A macrolide should be included when ‘atypical’ pathogens (e.g. Mycoplasma spp., Chlamydophila spp., pertussis) are suspected (evidence level IVa)

For ambulatory treatment of pneumonia, amoxicillin (45 mg/kg/ dose 12-hourly) remains the preferred antibiotic for children >1 month old. Outpatient management should not be considered for infants <1 month of age (Table 5).

HIV infection or exposure influences investigation and management of children with pneumonia (see below: Special circumstances: HIV infection or exposure)

**Table 5:** Empiric antibiotic therapy

**Age**		**Outpatients**		**Inpatients**	
0 - 1 month		Children <1 month of age should be hospitalised		Ampicillin 50 mg/kg IV 6-hourly, or benzylpenicillin 50 000 U/kg IM/IV 6-hourly and gentamicin 7.5 mg/kg IM/IV daily	
				If poor response	
				Ceftriaxone 50 mg/kg IV 12-hourly × 5d or	
				Cefotaxime 50 mg/kg IV 8-hourly × 5d	
				If cultures are negative, switch to oral amoxicillin-clavulanate when clinically improving and taking well orally – complete a total antibiotic duration of 5d.	
				If cultures are positive, use targeted therapy according to the organism’s susceptibility pattern.	
				Step down to oral antibiotic therapy as soon as the patient is clinically stable.	
				**Add**	
				Azithromycin 10 mg/kg daily orally × 5d if	
				*C trachomatis* is suspected.	
				(alternative: clarithromycin 7.5 mg/kg/d orally	
				12-hourly × 5d; erythromycin is	
				contraindicated in this age group).	
>1 month		Amoxicillin 45 mg/kg/dose		Amoxicillin-clavulanate 30 mg/kg/dose	
		12-hourly orally × 5d		(of amoxicillin component)	
		If poor response		8-hourly IV × 5d or	
		Amoxicillin-clavulanate 45 mg/kg/dose 12-hourly × 5d		Amoxicillin-clavulanate 45 mg/kg/dose orally 12-hourly × 5d	
				If cultures are positive, use targeted therapy,	
		**Add**		according to the organism’s susceptibility pattern.	
		Azithromycin 10 mg/kg orally daily × 5 d if *M. pneumoniae, C. pneumoniae* *or C. trachomatis* suspected (alternatives: clarithromycin 7.5 mg/kg/d orally every 12 h for 10 d or erythromycin 50 mg/kg/d for 10 - 14 d)		Step down to oral antibiotic therapy as soon as the patient is clinically stable. For susceptible *S. aureus*, use Flucloxacillin 50 mg/kg orally 6-hourly × 2 - 4 weeks If poor response Ceftriaxone 50 mg/kg IV 12-hourly × 5 d or Cefotaxime 50 mg/kg IV 8-hourly × 5 d	
				**Add**	
				Vancomycin 10 - 20 mg/kg/dose 6-hourly or Clindamycin for suspected CA-MRSA 1 month - 16 years: 20 - 40 mg/kg IV or IM/d, in 3 - 4 equally divided doses Use higher doses for treatment of more severe infections	
				**Add**	
				Azithromycin 10 mg/kg orally daily × 5 d if *M. pneumoniae, C. pneumoniae*	
				or *C. trachomatis* suspected (alternative: clarithromycin or erythromycin)	

IV = intravenous

IM = intramuscular

CA-MRSA = community-acquired methicillin-resistant *S. aureus*

**Route of administration**

Oral therapy is preferable; however, parenteral antibiotics should be used for children requiring intensive care unit (ICU) admission or for those too ill to tolerate oral medication. There are, however, risks and costs associated with intravenous use, and oral de-escalation is recommended as soon as feasible (evidence level IIIa).^[102,103]^

**Duration of antimicrobial therapy**

In general, 5 days of antibiotic therapy is recommended, but longer duration may be needed in children with severe or complicated disease, if there is a poor response to therapy, or as informed by microbiology results.

Bacteraemic staphylococcal pneumonia should be treated for 14 - 28 days, dependent on complications and response to treatment.^[104]^ Uncomplicated presumed staphylococcal pneumonia (i.e. blood culture negative, but with suggestive clinical or CXR features) may be appropriately managed with a 7- to 10-day course of targeted antibiotic therapy, depending on clinical response (evidence level IVa).^[105]^

### Management of a child who is not responding to therapy

A poor response to treatment has many possible explanations. Consider infection with *M. tuberculosis*, viruses, fungi or atypical organisms. Evaluate for the presence of a foreign body, empyema, heart disease or underlying immunodeficiency.^[106]^ HIV infection is by far the most common immunodeficiency state to consider in SA children with recurrent or severe episodes of pneumonia. If HIV infection is excluded through age-appropriate testing, primary immunodeficiencies should be considered in the differential diagnosis.

Change to amoxicillin-clavulanate if there is a poor clinical response or deterioration in a child treated with amoxicillin. For children initially treated with amoxicillin-clavulanate, change to ceftriaxone (evidence level IVa).^[17]^ Where laboratory support is available, it is strongly recommended to repeat a microbiological work-up, including blood culture, before changing antibiotics.

## Summary: Antibiotic therapy for community-acquired pneumonia

1. Oral amoxicillin is recommended for children >1 month of age who do not require hospitalisation (evidence level Ia).
  - Treatment duration should be 5 days, with review after 3 days to evaluate response.
  - Switch to amoxicillin-clavulanate if there is clinical deterioration, and consider referral for further investigation.
2. Children <1 month of age should be hospitalised and treated with ampicillin and an aminoglycoside (evidence level Ib).
3. Amoxicillin-clavulanate (intravenously or orally) is recommended for treatment of hospitalised children >1 month of age with severe pneumonia (evidence level IVa).
  - Treatment duration should be 5 days.
  - If there is clinical deterioration, switch to ceftriaxone or cefotaxime for 5 days (evidence level IVa).
4. Treatment duration should be prolonged for severe or complicated disease, and depend on microbiology testing.
  - Bacteraemic *S. aureus* pneumonia may require 14 - 28 days of antibiotic therapy, depending on clinical response (evidence level IVa).
5. Macrolide antibiotics should be used if pertussis, mycoplasma or chlamydia is suspected (evidence level IVa).

### Adjunctive therapies

**Antiviral treatment**

Oseltamivir is of limited benefit and is not recommended for routine use. Consider use during the influenza season in children at high risk for severe influenza, who present soon after symptom onset.^[8,24-36]^

**Corticosteroid therapy**

Corticosteroids should be used in children with suspected or confirmed pneumocystis pneumonia (PCP) (see below: Special circumstances: HIV infection or exposure ‒ Treatment), or in pulmonary tuberculosis with nodal airway compression and obstruction.^[107,108]^

**Vitamin and micronutrient supplementation**

Vitamin A
  Vitamin A supplementation reduces severity of respiratory complications of measles,^[109]^ but is not recommended for routine use.^[110]^ Consider routine vitamin A supplementation for HIV-infected or malnourished children with CAP.^[111]^
Vitamin D
  Vitamin D supplementation does not appear to improve CAP outcomes and is not routinely recommended (evidence level Ia).^[112–115]^

### Summary: Adjuvant therapies

1. Oseltamivir should be considered as early empiric therapy in children at risk of severe influenza-related pneumonia, who are hospitalised during the influenza season (evidence level II).
2. Routine use of corticosteroids for childhood CAP is discouraged (evidence level Ib).
3. Vitamin A is indicated for measles-associated pneumonia, or in those with vitamin A deficiency (evidence level Ib).
4. Do not routinely use vitamin D supplementation (evidence level Ia).

### When can a hospitalised child be discharged?

Apyrexial children no longer requiring oxygen, with adequate oral intake and acceptable home circumstances, can generally be safely discharged (Table 6).^[22]^

### General and supportive measures

**Oxygen therapy**

Assess oxygenation with regular pulse oximetry. If <92% at sea level (or <90% at altitude ≥1 800 m), administer oxygen via nasal cannula or face mask to maintain oxygen saturation 92 - 94% (evidence level II).^[116]^ If pulse oximetry is unavailable, administer oxygen if there is central cyanosis, grunting, restlessness, inability to drink or feed, or if respiratory rate is ≥70/breaths per minute.^[117]^

**Respiratory support**

High-flow humidified nasal oxygen (HFHNO) or nasal continuous positive airway pressure (nCPAP) systems provide support to children with severe respiratory disease (see below: Care of the child with pneumonia in the paediatric ICU or high care).^[118,119]^ These can be safely provided in adequately staffed and equipped high-care areas and district hospitals.^[119,120]^

**Blood transfusion**

In general, children who are haemodynamically stable should not be transfused if the haemoglobin (Hb) level is ≥7 g/dL (evidence level Ib); if their Hb is <5 g/dL, then transfuse packed red cells to raise the Hb to above the transfusion threshold (i.e. not to ‘normal ranges’) (evidence level II). For children with Hb 5 - 7 g/dL, evaluate their overall clinical status when deciding whether to transfuse.

**Fluids and electrolytes**

Fluid overload is associated with worse outcomes in severe CAP, particularly in those undergoing mechanical ventilation.^[121–123]^ Hyponatraemia is common secondary to high antidiuretic hormone secretion and is related to the severity of infection,^[124–128]^ but is less likely with isotonic intravenous manintenance fluids.^[129]^

Generally, children should be fed enterally; if this is not possible, then intravenous isotonic fluids should be administered at <80% of maintenance, with monitoring of sodium levels.

**Antipyretics and analgesia**

Hospitalised children with pneumonia, who often have fever and may have chest pain, should receive treatment,^[130,131]^ including:^[132]^

- paracetamol, orally (loading dose 20 mg/kg/dose, then 15 mg/kg/ dose 4- to 6-hourly)
- ibuprofen, orally (10 mg/kg/dose 8-hourly) with meals
  - where an anti-inflammatory effect is required
  - can be used in combination with paracetamol or opioids
- tilidine (1 drop per 2.5 kg body weight, i.e. 1 mg/kg/dose)
  - intermediate-efficacy opioid.

### Other measures

Over-the-counter cough medications are not effective in the management of CAP (evidence level Ia).^[133]^

Chest physiotherapy may be of benefit for children with lobar collapse,^[134]^ or when used in conjunction with nebulisation;^[135]^ however, routine chest physiotherapy is not recommended (evidence level Ia).^[136,137]^

Vaccination status should be reviewed and catch-up provided, including booster immunisation, as indicated.

## Summary: Treatment of pneumonia – supportive measures

1. Children with room air oxygen saturations of <92% (at sea level) or <90% (at altitude ≥1 800 m) should be treated with oxygen (evidence level II).
2. Most children may receive oxygen via nasal cannula, but the route of oxygen administration should be individualised (evidence level IVa).
3. Children who are haemodynamically stable should not be transfused if their Hb is ≥7 g/dL (evidence level II).
4. Children should be fed enterally; if this is not possible, administer intravenous isotonic fluids at <80% of maintenance with monitoring of sodium levels (evidence level Ib/II).
5. Children with fever or chest pain should be treated with appropriate antipyretics or analgesics (evidence level IVa).
6. Over-the-counter cough medications are not recommended (evidence level Ia).
7. Chest physiotherapy may benefit children with lobar collapse (evidence level Ia).
8. Vaccination status should be reviewed and catch-up provided, including booster immunisation, as indicated (evidence level IVb).

## Care of the child with pneumonia in the paediatric intensive care or high-care unit

### Introduction

A proportion of hospitalised children with CAP require admission to high care or the paediatric ICU (PICU); many of them have comorbid disease or other underlying susceptibilities.

### Admission criteria

Specific criteria for PICU admission depend on available resources and vary between institutions (Table 7).

### Investigations

**Microbiology**

See ‘Aetiological diagnosis’ and ‘Aetiology of pneumonia in children’.

**Radiological investigations**

These should be individualised to evaluate for complications (e.g. pleural effusion, pneumothorax or segmental/lobar collapse), and associated cardiac disease. CXR or point-of-care chest ultrasound, where available, is recommended on admission to the PICU to define central line positioning, following endotracheal intubation, or with any significant deterioration.^[138]^

### Oxygen therapy and monitoring

Clinical signs are inadequate for detecting hypoxia;^[139]^ therefore, continuous pulse oximetry monitoring is required. 

### Respiratory support

**High-flow oxygen**

See above: General and supportive measures: Respiratory support.

**Nasal continuous positive airway pressure systems**

nCPAP use for children with severe pneumonia (including bronchiolitis) is increasing as evidence emerges that supports its safety and efficacy.^[140–144]^

**Invasive ventilation**

Invasive ventilation is best provided in units experienced in such care, but often needs to be initiated prior to referral. High-frequency oscillatory ventilation may be considered for children requiring high mean airway pressure (MAP) using conventional ventilation.^[145]^

Children with significant airflow obstruction or hypercapnia require special consideration. Higher positive end-expiratory pressure (PEEP) and MAP may be required for children with refractory hypoxia (evidence level II).^[146]^

### Nutrition

Critically ill children should be provided with enteral nutrition as soon as possible (evidence level Ib).^[147,148]^

Many ventilated children receive inadequate dietary intake, and provision of a higher proportion of prescribed dietary goals is associated with improved outcomes.^[149]^ Parenteral nutrition administration is associated with higher mortality and increased complications (evidence level Ib).^[149–151]^

Children on HFHNO or non-invasive positive-pressure ventilation may receive enteral feeding without risk of aspiration.^[152,153]^

### Antibiotic therapy

While PICU-specific issues should be considered, the principles of antibiotic therapy are similar as for other children with CAP. Consider broader antimicrobial therapy for children whose clinical status worsens despite initial empiric therapy. Therapy should be deescalated based on microbiology investigations. Extrapolating from adult ICU evidence, procalcitonin levels may guide discontinuation of antibiotics,^[154]^ although there are limited paediatric data.^[155]^

### Corticosteroids, fluid and blood transfusion

These should be administered as per children with CAP managed outside of the ICU (see above: Adjunctive therapies: Corticoisteroid therapy).

### Physiotherapy

Current evidence is insufficient to provide strong recommendations for chest physiotherapy in the PICU.^[156,157]^

## Summary: High care and intensive care of children with pneumonia

1. Where possible, blood cultures should be obtained from children requiring PICU admission, but should not delay initiation of antibiotic therapy (evidence level III).
2. CXR or chest ultrasound should be done to identify complications at PICU admission and after interventions, such as endotracheal intubation, chest drain or central line placement (evidence level III), and after clinical deterioration (evidence level III).
3. Oxygen saturation levels should be monitored continuously (evidence level IVa). Where possible, FiO_2_ should be adjusted to achieve saturations of 92 - 96% (evidence level III).
4. nCPAP improves outcomes compared with nasal cannula oxygen (evidence level Ib), while nCPAP and HFHNO have similar efficacy in patients with severe bronchiolitis (evidence level Ib).
5. Antibiotic therapy should be de-escalated and discontinued as soon as possible (evidence level II).
6. Routine chest physiotherapy should not be provided for children (evidence level III), although some patients may benefit. Ongoing chest physiotherapy should be based on clinical improvement and lack of clinical deterioration (evidence level III).

## Prevention of childhood pneumonia

### General preventive strategies 

General preventive strategies that reduce the incidence and severity of pneumonia are the following, and are summarised in Table 8:

**Nutrition**

Adequate nutrition and growth monitoring should be encouraged, as malnutrition predisposes children to pneumonia and severe illness. Breastfeeding has been shown to decrease the incidence of pneumonia in young children by up to 32%.^[158]^ Shorter duration of breastfeeding is associated with pneumonia mortality, particularly among infants <5 months of age.^[159]^ Mortality among infants who are not breastfed compared with exclusively breastfed infants through 5 months of age is ~15-fold higher.^[159]^ Breastfeeding should be encouraged for the first 6 months of a child’s life, irrespective of maternal HIV or ART use,^[160]^ and may be considered for the first 2 years in CLWH.^[161]^

**Micronutrient supplementation**

Specific micronutrients that may play a role in the prevention of pneumonia are discussed below.

Vitamin A
  Vitamin A supplementation reduces severity of respiratory complications of measles.^[109]^ However, a meta-analysis of the impact of vitamin A supplementation on all-cause pneumonia morbidity and mortality showed no consistent effect on pneumonia-specific mortality.^[110]^ Provision of vitamin A supplementation in children with vitamin A deficiency has been associated with improved outcomes. Vitamin A supplementation should be administered as per national guidelines: 100 000IU at 6 months, 200 000IU at 12 and 18 months, and 200 000IU every 6 months from 2 to 5 years of age.^[162]^
Vitamin D
  While empiric therapy using vitamin D in hospitalised children with CAP is not beneficial, observational studies have identified an increased risk of pneumonia in children <5 years old with subclinical vitamin D deficiency (evidence level III).^[163]^ A meta-analysis of the role of vitamin D supplementation in pneumonia prevention found a significant protective effect (evidence level Ia).^[164]^ However, in a clinical trial conducted in Asian children <5 years of age, oral doses of vitamin D had no protective effect on the incidence of the first episode of pneumonia (evidence level Ib).^[165]^ Currently, there is lack of consensus as to what serum levels of vitamin D are protective against LRTI, and also little evidence as to the best supplemental regimen (evidence level Iva).^[113]^ British guidelines recommend a daily intake of 400 IU vitamin D, regardless of age group (evidence level Ia).^[166]^
Vitamin E
  There is very little evidence to support vitamin E supplementation for the prevention of pneumonia in children (evidence level IVa).^[167,168]^
Zinc
  Daily prophylactic elemental zinc, 10 mg (infants) and 20 mg (older children), may substantially reduce the incidence of pneumonia, particularly in malnourished children.^[169]^ A pooled analysis of randomised controlled trials of zinc supplementation in wellnourished and malnourished children found that children who received zinc supplementation had a significant reduction in pneumonia incidence compared with those who received placebo (odds ratio (OR) 0.59; CI 0.41 - 0.83) (evidence level Ia).^[170]^

**Reduction in tobacco smoke or indoor fuel exposure**

Active and passive exposure to tobacco should be strongly discouraged in women of child-bearing age, particularly among pregnant women,^[171]^ and more generally in the household.^[172]^

Exposure to fumes from indoor cooking fuels should be limited by opening windows and doors when cooking; the chimney should function well; the stove should be cleaned and maintained; and there should be safer child location practices while fires are burning in the house.^[173]^ The practice of carrying children on caregivers’ backs while cooking is as an independent risk factor for LRTI morbidity and mortality.^[174]^ Children should sleep in rooms separate from where food is cooked or where open fires or paraffin burners are used (evidence level Ib).

**Infection prevention, control, use of masks and physical distancing**

Hand hygiene and respiratory etiquette are crucial in limiting transmission of respiratory pathogens.^[175,176]^ Reinforcement of hand hygiene decreases the prevalence of respiratory tract illness in adults by 14% (95% CI 11 - 17) in non-pandemic influenza seasons^[177]^ A systematic review and meta-analysis of the effect of hand hygiene in limiting illness in children suggested that in primary and secondary schools, hand hygiene may decrease the incidence of respiratory tract infections among learners (evidence level Ia).^[178]^ Although young children are not generally able to adhere to respiratory etiquette practices,^[179]^ older children, caregivers and health workers should adopt these practices to limit the transmission of respiratory pathogens.^[175]^

Universal use of cloth face masks by children and adults in public is an effective public health intervention to reduce transmission of respiratory viruses, including SARS-CoV-2, in addition to other public health measures.^[180]^ In health facilities, all healthcare workers should wear a surgical mask in addition to practising hand hygiene, physical distancing and environmental decontamination to prevent SARS-CoV-2 transmission. N95 or respiratory masks should be used when taking care of children with confirmed or suspected COVID-19, or when doing aerosolising procedures.^[180,181]^

### Specific preventive strategies

**Immunisation**

Routine immunisations
  All children should receive routine vaccines, including bacillus Calmette-Guérin (BCG), measles, diphtheria-pertussis-tetanus (DPT) toxoid, Hib, polysaccharide-protein conjugate vaccine (HibCV) and PCV as per the SA immunisation schedule.^[182]^ The nature and degree of immunosuppression in CLWH may impact on the efficacy and duration of vaccine-induced protection.^[183–187]^ CLWH treated with ART from early infancy, and responding well to such therapy, demonstrate similar immunogenicity and anamnestic immune responses to most childhood vaccines compared with HIV-unexposed infants.^[188,189]^ HEU infants may have lower concentrations of transplacental acquired antibodies for some vaccine-preventable diseases,^[190,191]^ which could increase their susceptibility to pneumonia during early infancy. The immune responses to all vaccines are, however, similar or more immunogenic for HEU than for HIV-unexposed infants,^[188,189]^ and there is similar persistence of protective antibody concentrations and memory responses.^[192]^
Specific vaccines
  **BCG vaccine.***M. tuberculosis* may be a direct pathogen in pneumonia or may predispose to bacterial infection (including from pneumococcus).^[70,193]^ A birth dose of BCG vaccine is effective in preventing disseminated tuberculosis in young children, but has variable effectiveness (average 50%; range 0 - 84% effectiveness) in prevention of pulmonary tuberculosis, with lower effectiveness in studies on children from tropical countries.^[194,195]^ A birth dose has also been shown to have nonspecific benefits in improving overall child survival in some settings.^[196]^
  **Pneumococcal vaccine.** Multiple post-licensure effectiveness studies (using 10- and 13-valent PCV) in a diversity of settings have demonstrated a 17% (95% CI 11 - 22) and 31% (95% CI 26 - 35 ) reduction in hospitalisation rates for clinically and radiologically confirmed pneumonia, respectively.^[197]^ In children aged 24 - 59 months a meta-analysis found a reduction of 9% (95% CI 5 - 14) and 24% (95% CI 12 - 33) in hospitalisation rates for clinically and radiologically confirmed pneumonia, respectively (evidence level Ia).^[197]^
    In SA, PCV (currently 13-valent) is administered at 6 and 14 weeks of age, followed by a booster dose at 9 months of age (evidence level Ib). This schedule has been shown to be effective in reducing all-cause pneumonia hospitalisation by 33% and 39% in CLWH and HIV-uninfected children, respectively.^[5]^
    The 23-valent pneumococcal polysaccharide vaccine (Pneumovax 23) is recommended for children >2 years old, who are at risk of developing invasive pneumococcal disease, including those with sickle cell disease, chronic pulmonary disease and cardiovascular disease,^[198,199]^ and is included in the SA Essential Drugs List for Paediatrics for administration to such patients. It should be preceded by a single dose of PCV, given at least 1 month before (evidence level III).^[200,201]^
    The need for further booster doses of PCV in older CLWH remains to be determined, but the indirect effect of childhood PCV immunisation in reducing transmission and circulation of vaccineserotype pneumococci could mitigate waning of immunity in CLWH and other high-risk groups that remain susceptible to developing severe pneumonia in later childhood.^[202]^ The WHO currently recommends that a booster dose of PCV may be considered in the second year of life in CLWH (evidence level II).^[203]^
  **Hib conjugate vaccine.** Vaccination with HibCV, as part of a combination vaccine, is recommended as a 3-dose primary series, and includes a booster dose at 15 - 18 months of age in SA. HibCV is less effective in CLWH not on ART;^[204]^ however, the immunogenicity of the vaccine and persistence of memory responses in the second year of life are similar in CLWH vaccinated when already initiated on ART at the time of immunisation.^[205]^
  **Pertussis vaccine.** Pertussis remains one of the most poorly controlled vaccine-preventable diseases globally and causes severe disease in young infants (especially in those <3 months of age) and incompletely immunised children (evidence level Ib).^[206,207]^
    Pertussis vaccine formulations include whole-cell containing (wP) and *B. pertussis* protein-only component acellular vaccines (aP). Whole-cell pertussis vaccines, but not aP protein-containing vaccines, induce mucosal immunity and protect against *B. pertussis* mucosal infection and transmission.^[208]^ Furthermore, the duration of protection of wP is 8 - 12 years compared with 4 - 5 years for aP vaccines.^[209]^ Currently, only aP-containing combination vaccines are available in SA.
    Pertussis outbreaks have been temporally associated with transitioning from wP to aP formulations in many high-resource settings, attributed to the waning of immunity in the absence of repeat booster doses at school entry and beyond.^[209]^ Children primed with aP-containing vaccines should receive booster doses of aP vaccines (dTaP) at school entry and possibly every 10 years thereafter (not yet part of the Expanded Programme on Immunisation (EPI)) (evidence level II).^[210]^
    Prevention of pertussis in very young infants, who are at greatest risk of severe disease, is not achievable through infant immunisation, and transitioning from wP to aP could increase the burden of pertussis in in this group.^[53]^ Acellular pertussis vaccination of pregnant women is 90% effective in reducing pertussis in infants <3 months of age (evidence level Ia).^[211]^
  **Influenza vaccine.** Only the sub-unit inactivated influenza vaccine is available in SA for annual administration. There are limited data on its efficacy in children, ranging from 33% to 73%, depending on vaccine preparation and influenza subtype targeted.^[212]^ Current evidence suggests that influenza vaccination is safe in CLWH; however, a randomised controlled trial failed to demonstrate vaccine efficacy.^[213]^ Nonetheless, there remains a recommendation that CLWH should be offered influenza vaccination before the start of winter, particularly if they have underlying chronic lung disease (evidence level III).
    Children >6 months of age with underlying medical conditions are considered to be a high risk for complications of influenza, and are prioritised for annual vaccination. Such children comprise those with chronic pulmonary disease (including asthma), cardiac disease, chronic renal or hepatic diseases, diabetes mellitus, metabolic disorders, sickle cell anaemia and other haemoglobinopathies, morbid obesity, immunosuppression, cerebral palsy or other neuromuscular conditions.^[214]^ Family members and siblings of such patients should also be vaccinated.^[214]^ Two doses of inactivated influenza vaccine, administered 1 month apart, are recommended for children 6 months - 9 years of age who have never been vaccinated; and a single dose if immunised in previous seasons.^[214]^
    Recent randomised controlled trials have demonstrated that influenza vaccination of pregnant women was 50% efficacious in reducing PCR-confirmed influenza illness in their infants until 24 weeks of age. In SA and Mali, vaccination of pregnant women was more effective in preventing influenza illness in infants during the first 3 months of life (vaccine efficacy ‒ 85%) with subsequent waning and a non-significant reduction between 3 and 6 months of age.^[215]^ Maternal influenza vaccination also reduced all-cause clinically diagnosed severe pneumonia or pneumonia hospitalisation by 30% in infants during the first 6 months.^[215,216]^
    Influenza immunisation should ideally be administered prior to the onset of the influenza season (which typically occurs from May to September in SA),^[73,217]^ but can also be given during the influenza season. Due to the potential of year-on-year genetic drift of seasonal influenza virus strains, current vaccine formulations are updated annually, and a repeat vaccination is required each year
  **Measles vaccine.** Measles remains a public health concern, and failure to achieve and sustain high immunisation coverage rates (>95%) against this highly contagious virus results in ongoing outbreaks in a diversity of settings, including SA.^[217,218]^ Recent changes in the epidemiology of measles include a greater susceptibility of disease in very young infants (as early as 4 months of age).^[219]^ This is due to lower antibody concentrations in pregnant women who have acquired immunity through vaccination, rather than through wild-type virus exposure, as well as possible waning of immunity in women living with HIV.^[220,221]^
    The WHO recommends that children receive 2 doses of the measles vaccine, the first at 9 months of age and a booster dose at 15 - 18 months of age.^[222]^ However, for infants born to women living with HIV, and in settings with a high risk of measles in young infants, an additional dose is recommended at 6 months of age.^[222]^ In SA, 2-dose measles vaccination is administered at 6 and 12 months of age. This induces seroprotective titres in ~55% of infants following the first dose of vaccine, and in >98% in HIV-exposed and HIV-unexposed children after the second dose of vaccine.^[223]^

**Combination antiretroviral therapy**

The use of ART to reconstitute immunity is very effective for decreasing the incidence of pneumonia and opportunistic infections in CLWH. Combination ART should be initiated on diagnosis of HIV in all children, irrespective of clinical or immunological staging. Screening for HIV infection in newborns of HIV-infected women by means of PCR testing at birth and repeated PCR testing during the infant and breastfeeding period is standard of care in SA, with initiation of ART as soon as possible after confirmation of HIV infection, and continued lifelong thereafter.

**Prophylaxis**

Prevention of *Pneumocystis jirovecii* pneumonia
  Updated recommendations for the management of CLWH were published in 2019 (SA ART guidelines: https://sahivsoc.org/Files/2019%20Abridged%20ART%20Guidelines%2010%20October%202019.pdf) (Table 9).^[224]^
    Although the WHO recommends PCP prophylaxis for HEU infants from 4 to 6 weeks of age until HIV infection has been excluded after complete cessation of breastfeeding, two southern African randomised controlled trials have shown that co-trimoxazole confers no survival advantage over placebo in this subset of children; therefore, this is not recommended in SA.^[225,226]^
Prevention of tuberculosis
  All children <5 years of age exposed to a household tuberculosis contact or other close tuberculosis contact should be given isoniazid preventive therapy (IPT) (10 mg/kg; maximum dose 300 mg) daily for 6 months once tuberculosis disease has been excluded. CLWH exposed to a household contact should be given prophylaxis for 6 months, irrespective of their age. A 6-month course of IPT should also be given to tuberculin skin test (TST)-positive HIV-infected children, even in the absence of a known household contact.^[227]^ There are conflicting data on the use of primary IPT in CLWH in the absence of a tuberculosis contact.^[228,229]^ Newly HIV-diagnosed and clinically symptomatic CLWH may benefit from a 6-month course of IPT, irrespective of TST results.
    Short-course preventive therapy using rifampicin and isoniazid must not be used in the context of tuberculosis prevention in HIVexposed neonates born to mothers with active tuberculosis, as the rifampicin component interferes with the prevention of mother-tochild transmission (ART) regimens.^[230]^
    Current WHO guidelines encourage use of preventive therapy with multidrug-resistant tuberculosis (MDR-TB) based on individualised risk assessment for children exposed to source cases. In children exposed to a source case with ofloxacin-susceptible *M. tuberculosis*, a 6-month course of ofloxacin, ethambutol and high-dose isoniazid has been found to be well tolerated.^[231]^
Prevention of cytomegalovirus disease in HIV-infected children
  There is no evidence to support a specific intervention in the prevention of CMV disease in CLWH.^[232]^
Prevention of respiratory syncytial virus
  Although the humanised monoclonal-specific antibody for the prevention of RSV infections (palivizumab) is available, it is very expensive. Children most likely to benefit are those at risk of severe RSV infection, i.e. babies born prematurely who are <6 months of chronological age at the onset of the RSV season, or children with chronic lung disease or congenital cardiac disease who are <1 year of age at the onset of the RSV season.^[233]^ A meta-analysis on the effectiveness of palivizumab against RSV hospitalisation reported 71% (95% CI 46 - 84) effectiveness in infants born at <35 weeks’ gestational age, and ~45% in those with chronic lung disease or congenital heart disease (evidence level Ia).^[234]^ Palivizumab should be given monthly for the duration of the RSV season (from February to July) in most of SA.^[235,236]^
    Other strategies for prevention of severe RSV disease in infants, including antenatal vaccination of expectant mothers and longacting monoclonal antibody preparations, are currently under investigation.

## Summary – prevention

1. Exclusive breastfeeding is recommended for all infants, irresepctive of HIV exposure status, for the first six months of life.
2. All children should receive BCG vaccine at birth.
3. All children should receive two doses of a primary series of 13-valent PCV during early infancy, at least two months apart (6 and 14 weeks of age), and a booster dose at 9 months of age (evidence level Ia).
4. 23-valent pneumococcal polysaccharide vaccine (Pneumovax 23) is recommended for children >2 years at risk of developing invasive pneumococcal disease, including children with chronic pulmonary or cardiovascular disease (evidence level III).
5. All children should receive at least three doses of HibCV. In SA, HibCV vaccination is recommended (as part of a combination vaccine) as 6, 10 and 14 weeks of age, and a booster dose at 15 - 18 months of age (evidence level Ib).^[205]^
6. All children should receive a three dose primary infant series of aP or wP containing vaccine at 6, 10 and 14 weeks of age; and a booster dose of vaccine at 15 - 18 months of age. In SA, only aPcontaining vaccine preparations are currently available.
7. Influenza vaccine is recommended annually for children at high risk of severe influenza disease. The recommended schedule of sub-unit inactivated influenza vaccine who have not been vaccinated is two doses, spaced 1 month apart. Children who are >9 years or those who have been immunised previously require only a single dose of vaccine. The vaccine should be administered before the start of the influenza season.
8. Pregnant women should receive inactivated influenza vaccine at any stage of pregnancy, and should be prioritised for vaccination.^[237]^
9. All children should receive measles vaccination at 6 and 12 months of age.^[222]^
10. ART is life-saving in CLWH, who should be expedited onto treatment as soon as the diagnosis is confirmed (evidence level Ia).
11. Co-trimoxazole preventive therapy is crucial for prevention of PCP in young CLWH, and in older, ART-naïve or severely immunosuppressed children (Evidence level Ia).
12. IPT is under-utilised in SA, but should be used in CLWH of any age or an immunocompetent child under 5 years, with a household contact with tuberculosis (evidence level II). IPT is also indicated for any CLWH who is TST positive.

## Special circumstances: HIV infection or exposure

HIV infection rates in children have declined significantly with the advent of effective PMTCT interventions, from 30% to 1 - 3% in SA.^[238]^ There is subsequently a large population of children born to HIV-infected mothers who are HEU.^[238]^ HEU infants have an increased risk of pneumonia, particularly in the first 6 months of life (evidence level Ib).^[10]^ The exact mechanisms of vulnerability in HEU infants may involve *in utero* exposure to HIV viral proteins, exposure to ART, lack of effective protective maternal antibodies, or abnormalities in immunological responses (evidence level III).^[238,239]^

Prior to the ART roll-out, CLWH had a 4 - 6-fold increased risk of developing severe pneumonia,^[14,240]^ and a 4 - 6-fold increased risk of death once hospitalised for pneumonia (evidence level II).^[9,240]^ However, widespread use of ART has markedly reduced this burden, with a decline in mortality rates among children on ART from 7.1 per 100 person-years to 0.6 per 100 person years in a multicentre prospective cohort study from the USA.^[241]^

HIV-infected infants not on ART, especially those with viral load ≥100 000 copies/mL and CD4 <15%, are at greatest risk of developing severe disease (evidence level Ib).^[242]^ Additional risk factors for severe disease include poor nutritional status and anaemia (evidence level Ib).^[240]^

### Aetiology of pneumonia in HIV-infected children

In the era of ART, the aetiology of pneumonia is similar amongst CLWH, HEU and HIV-unexposed children (Supplementary Tables 1 and 2 - http://ajtccm.org.za/public/sup/104.pdf)^[243]^ However, in HIV-infected infants, *P. jirovecii* is still the leading cause of hospitalisation.^[62]^

*M. tuberculosis* should be considered in HIV-infected children with pneumonia, as evidence suggests an increased risk for tuberculosis (evidence level Ib).^[240]^ HIV-infected infants not on ART have a 20-fold higher risk of developing culture-confirmed tuberculosis compared with HIV-uninfected infants (evidence level 1b).^[244]^ This risk declines with the introduction of ART.^[245]^

### Treatment

Empiric CAP treatment of HEU infants and HIV-infected children is the same as for HIV-unexposed children. There are currently no studies comparing regimens for and outcomes of HIV-infected and HEU infants.^[246]^ HIV infection should be considered in all children with CAP, and appropriate testing must be provided (evidence level IVa).

**Pneumocystis pneumonia**

Few children currently acquire vertically transmitted HIV infection ‒ most of these are diagnosed through early infant testing and receive ART and co-trimoxazole prophylaxis.^[247]^ Women who tested HIV-negative during pregnancy may acquire HIV infection late in gestation or while breastfeeding ‒ their infants are at risk of PCP (evidence level Ib).^[248–251]^ Some children with primary immunodeficiencies or severe malnutrition are also at risk of PCP (evidence level IIb).^[252]^ Empiric therapy for PCP should not be withheld pending results of confirmatory laboratory testing.

Co-trimoxazole administered intravenously or orally, 5 mg trimethroprim/25 mg sulfamethoxazole/kg/dose, 6-hourly for 21 days, reduces mortality from PCP in infants.^[253]^ Following treatment, daily co-trimoxazole prophylaxis needs to be continued until CD4+ counts recover as per SA ART guidelines (Table 9).^[224]^ There is conflicting evidence regarding the effect of adjunctive corticosteroids.^[254]^ However, we recommend a short course of corticosteroids for children with PCP, initiated within 72 hours of diagnosis (prednisone 1 - 2 mg/kg orally daily for 7 days, tapered over the next 7 days) (evidence level IVa).^[255]^

**Cytomegalovirus**

CMV pneumonia should be considered in HIV-infected or HEU infants <6 months of age with severe hypoxaemia or requiring mechanical ventilation.^[94,256]^ The optimal treatment remains unclear. Some clinicians initiate treatment if the whole-blood CMV viral load is >4.1 log_10_ copies/mL,^[257]^ but this may delay therapy. Alternatively, initiate empiric ganciclovir and discontinue if CMV viral load results indicate low-level or absent viraemia (evidence level IVa). 

In children with high-level CMV DNAemia, clinical improvement and/or a decline in viral load should prompt switching from intravenous ganciclovir to oral valganciclovir (evidence level IVa). Ganciclovir should be given at 5 mg/kg intravenously 12-hourly until oral therapy is tolerated, then switch to valganciclovir 16 mg/kg orally 12-hourly until completion of 21 days of treatment. Thereafter, administer valganciclovir 16 mg/kg orally daily to complete a total of 42 days of therapy.^[106]^

**Atypical organisms**

If ‘atypical’ organisms are considered in the differential diagnosis, a macrolide should be added (see above: Treatment of childhood pneumonia: Which empiric antibiotic?) (evidence level IVa).

## Summary – HIV infection or exposure

1. A broader range of pathogens is responsible for pneumonia in CLWH, encompassing opportunistic infections such as PCP and CMV, *S. aureus* and gram negative organisms (evidence level Ib).
2. *M. tuberculosis* should be considered in CLWH who have an increased risk for tuberculosis (evidence level Ib).
3. Empiric antibiotic treatment is the same in CLWH, HEU and HIV unexposed children, although treatment for PCP (evidence level Ib) and/or CMV pneumonia (evidence level IVa) should be considered in HIV-infected infants with severe hypoxaemia.

## Supplementary Tables

**Epidemiology and Aetiology of Community-Acquired Pneumonia in Children** – South African Thoracic Society Guidelines (Part 1)
Supplementary Table 1: Bacterial Organisms Implicated in Childhood Community-acquired Pneumonia
Supplementary Table 2: Respiratory Viral Pathogens Implicated in Childhood Community-acquired Pneumonia

## Figures and Tables

**Table 1:** Categories of pneumonia – World Health Organization classification^[2]^

**Category**		**Characteristics**
Severe pneumonia		**Any child with a general danger sign**	
		Inability to drink	
		Convulsions	
		Abnormal sleepiness	
		Persistent vomiting	
		*or*	
		Oxygen saturation <90% (at altitude >1 800 m) or <92% at sea level or central cyanosis	
		*or*	
		Severe respiratory distress (grunting, very severe chest indrawing)	
		**Infant <2 months of age with**	
		A general danger sign	
		*or*	
		Chest wall indrawing	
		*or*	
		Tachypnoea (≥60 breaths per min)	
		**Children living with HIV,** **immune-compromised or malnourished children with**	
		Lower chest indrawing	
	
Pneumonia		**Child >2 months of age with**	
		Lower chest indrawing	
		*or*	
		Tachypnoea	
		≥50 breaths per min for infants 2 - 11 months of age	
		≥40 breaths per min for children 1 - 5 years of age	
	
No pneumonia		No signs of pneumonia or severe pneumonia, i.e. upper respiratory tract infection	

**Table 2:** Indications for hospital admission

**All children <2 months of age**
**Children >2 months of age with**
A general danger sign
Grunting, severe lower chest indrawing
Stridor in a calm child
Room air arterial oxygen saturation <92% at sea level or <90% at high altitude, or central cyanosis
Severe malnutrition
**HIV-infected, immune-compromised or malnourished child with lower chest indrawing**
**Family unable to provide appropriate care**

**Table 3:** Indications for chest X-ray (evidence level Ib)

• Severe pneumonia
• Suspected pulmonary tuberculosis
• Suspected foreign body aspiration
• Pneumonia unresponsive to standard management
• Consider in children <5 years of age, presenting with fever (>39°C), leukocytosis and no obvious source of infection, as ~18% of such patients have radiographic pneumonia (evidence level III)^[14,15]^

**Table 6:** Criteria for discharge from hospital*

• Clinical improvement, indicated by improved activity, appetite, and resolution of fever for at least 12 hours. Do not discharge if increased work of 7 breathing or tachycardia
• Pulse oximetry measurements consistently ≥90% at altitude (≥1 800 m) or ≥92% at sea level in room air for at least 12 hours
• Stable and/or return to baseline mental status
• If a chest tube was placed, no intrathoracic air leak for at least 12 - 24 hours after removal of the tube
• Ability to administer antibiotics at home, and child able to tolerate oral feeding and antibiotics
• Acceptable home circumstances and ability to return to hospital if clinical deterioration

* Adapted from the Pediatric Infectious Diseases Society and the Infectious Diseases Society of America guidelines.^[20]^

**Table 7:** Indications for paediatric intensive care unit admission

**• Rapidly deteriorating clinical condition despite appropriate management**
**• Need for respiratory support as evidenced by**
• apnoea (particularly in small infants)
• increasing oxygen requirements, i.e. any child requiring FiO_2_ >60% to maintain arterial saturations >88%^[43–45]^
• increasing effort of breathing (as assessed by respiratory rate, chest-wall retractions, noisy breathing),
with imminent respiratory collapse.
• hypercarbia resulting in respiratory acidosis
**• Deterioration in level of consciousness or seizures, particularly if any concern about maintaining airway patency and avoiding aspiration**
**• Cardiovascular instability as reflected by severe tachycardia/hypotension/inotrope requirement**

**Table 8:** Summary: Measures to prevent pneumonia in children

**General preventive strategies**	**Summary**
**Nutrition**	Malnourished children are at increased risk for severe pneumonia and mortality
	Breastfeeding is protective
**Micronutrient supplementation**	
Vitamin A	Vitamin A should be dosed according to the Road to Health card schedule
	100 000 IU stat at 6 months;
	200 000 IU stat at 12 months;
	200 000 IU stat at 18 months.
	From 24 months onwards,
	200 000 IU every 6 months from 2 to 5 years of age.
Vitamin D	Vitamin D-deficient children are at increased risk for CAP,
	supplement with vitamin D 400 IU daily.
Zinc	Zinc 10 mg (for infants) and 20 mg (for older children) daily significantly reduces the risk of pneumonia.
**Reduction in passive smoking and indoor fuel exposure.**	Environmental exposure to cigarette smoke or indoor air pollution is strongly correlated with impaired lung health in children.
**Infection prevention and control and physical distancing.**	Careful attention to limiting transmission of respiratory pathogens reduces the burden of respiratory illness.
	Hand hygiene
	Cough etiquette
	Decontamination of environmental surfaces
	Use of masks
	
**Specific preventive strategies**
**Immunisation**	Administered at:
BCG	Birth
Pneumococcal conjugate vaccine	6 weeks, 14 weeks and 9 months
Hib conjugate vaccine and pertussis vaccine	6 weeks, 10 weeks, 14 weeks and 18 months
	as part of the hexavalent vaccine.
Influenza vaccine	Not routinely administered in the SA EPI, but should be
	considered annually for children ≥6 months of age at risk
	for severe influenza, including those with congenital
	cardiac disease, chronic lung disease, immunosuppression
	and neuromuscular disease
Measles-containing vaccine	6 months and 12 months
**Combination ART**	Expeditious initiation of ART at the earliest opportunity must be implemented routinely for all CLWH to restore immunological function and prevent infectious complications of HIV; ideally, the diagnosis should be made at birth and ART initiated within the first week of life, as per National ART guidelines
**Prophylaxis**	
Prevention of PCP	Co-trimoxazole prophylaxis is crucial in the prevention of PCP and all-cause mortality in CLWH and those with immunosuppression from other causes; refer to the SA paediatric ART guidelines for details and Table 9.
Prevention of tuberculosis	INH is an under-utilised preventive strategy in SA children exposed to a household contact with tuberculosis – INH 10 mg/kg × 6 months is recommended CLWH or other underlying immunosuppression with a positive tuberculin skin test, even in the absence of a contact, should be given INH × 6 months. This may also be considered for children newly diagnosed with HIV. The maximum daily dose of INH is 300 mg.
Prevention of CMV	Although CMV pneumonia is considered to be an important infection in HIV-infected infants, no recommendation on chemoprophylaxis has been adopted
Prevention of RSV	The cost of monoclonal antibody prophylaxis (palivizumab) against RSV is very high ‒ therefore, widespread use is not feasible at a programmatic level; ex-premature infants <6 months of age, and those with congenital cardiac disease or chronic lung disease <1 year of age during the course of the RSV season, benefit most from this preventive measure given monthly through the season

CAP = community-acquired pneumonia

BCG = bacillus Calmette-Guérin

Hib = *Haemophilus influenzae* type b

SA = South Africa

EPI = Expanded Programme on Immunisation

ART = antiretroviral therapy

CLWH = children living with HIV

PCP = *Pneumocystis jirovecii* pneumonia

IPT = isoniazid-preventive therapy

INH = isoniazid

CMV = cytomegalovirus

RSV = respiratory syncytial virus

**Table 9:** Indications for co-trimoxazole prophylaxis in children living with HIV

**Indications to start prophylaxis**
Children 6 weeks - 1 year of age, irrespective of clinical stage or immunological status
Children 1 - 5 years of age with
CD4+ counts ≤25% or WHO stage 2 or greater
Children ≤5 years of age with
Prior PCP
Children >5 years of age with
CD4+ counts ≤200 cells/µL or WHO stage 2 or greater

**Indications to discontinue prophylaxis**
Children 1 - 5 years of age with
CD4+ counts >25%, regardless of clinical stage
Children >5 years of age with
CD4+ counts >200 cells/µL, regardless of clinical stage

WHO = World Health Organization

PCP = Pneumocystis jirovecii pneumonia
