# Supplementary material for: Diagnosis and management of community-acquired pneumonia in children: South African Thoracic Society guidelines
Source: Afr J Thorac Crit Care Med. 2020 Oct 13;26(3):10.7196/AJTCCM.2020.v26i3.104. doi: 10.7196/AJTCCM.2020.v26i3.104 (PMC7433705; doi:10.7196/AJTCCM.2020.v26i3.104)
Supplement: Supplementary file 1 — Epidemiology and Aetiology of Community-Acquired Pneumonia in Children – South African Thoracic Society Guidelines (Part 1) Supplementary Table 1: Bacterial Organisms Implicated in Childhood Community-acquired Pneumonia Supplementary Table 2: Respiratory Viral Pathogens Implicated in Childhood Community-acquired Pneumonia [file AJTCCM-26-3-104-S001.pdf]

Epidemiology and Aetiology of Community-Acquired Pneumonia in Children – South African Thoracic Society Guidelines (Part 1)
Supplementary Tables

Supplementary Table 1: Bacterial Organisms Implicated in Childhood Community-acquired Pneumonia				
Pathogen	Clinical Features	Populations at Risk	Other Risk Factors	Comments
Streptococcus pneumoniae	Common cause of otitis media.[1-3] Pneumococcal pneumonia usually manifests as high-grade fever, radiologically-confirmed pneumonia, elevated CRP,[4] rapid defervescence after receipt of antibiotic therapy. Occasionally presents with pleural effusion/ empyema.	Children with HIV, or HEU children; Children with missed doses of PCV; Children with chronic lung disease;[5-7] Children with PTB.[8]	Vaccine-serotype pneumococcal disease is more prevalent in children living with HIV (CLWH) and HEU children. Malnutrition, household crowding, household air pollution and second hand smoke exposure are also important risk factors.[5-7]	Pneumococcus was the single largest contributor to hospitalised bacterial CAP in young children in the pre-PCV era.[9]
Non-type b *Haemophilus influenzae*	Common cause of otitis media and pharyngitis.[2, 3, 10, 11] Radiologically-confirmed pneumonia, fever, elevated CRP. Occasionally presents with pleural effusion/ empyema.	Children with HIV, or HEU children; Children with chronic lung disease.		In the era of widespread access to HibCV, non-type b *H. influenzae* is implicated in a greater proportion of child CAP cases than is Hib.[12]
Haemophilus influenzae type b (Hib)	Radiologically-confirmed pneumonia, fever. Often complicates with invasive spread, e.g. pleural effusions, empyema, osteomyelitis, epiglottitis, and meningitis.	In the pre-HibCV era, CLWH had a greater risk of developing invasive Hib disease.[13] CLWH have less robust responses to vaccination	Children unvaccinated with HibCV, living in isolated communities with low herd immunity to Hib.	In the era of widespread HibCV coverage, invasive disease attributed to Hib has become a rarity.

		are than do HIV-uninfected children.[14, 15]		If encountered in the clinical setting, Hib must be notified (Category 2 Notifiable Medical Condition).[16]
Staphylococcus aureus	Occasionally presents as pharyngitis or otitis media.[3, 10]<br>High-grade fever, radiologically-confirmed pneumonia often associated with pneumatocoeles and 'breakdown' on chest radiograph. The most prevalent organism identified in empyema in the PCV era.[17, 18]	Children with HIV, especially once graduated beyond the PCP 'at risk' age category (>12 months of age).[19]<br>Being pre-ART, or having underlying chronic lung disease, are important risk factors in CLWH.[13, 20]	Foreign body aspiration is an important contributor to *S. aureus* lobar pneumonia and empyema in older children.[21, 22]	In South Africa, most CAP associated with *S. aureus* emanates from cloxacillin-susceptible strains.<br>If fever does not settle on targeted antibiotic therapy, look for metastatic *S. aureus* disease (endocarditis, osteomyelitis, soft tissue collections).
Streptococcus pyogenes (GAS)	Common cause of pharyngitis and otitis media.[3, 10, 11]<br>Occasionally presents as pneumonia, which may complicate with pleural effusions or empyema.	HIV-infection, malnutrition and presence of skin lesions (e.g. burns, eczema, scabies and varicella-zoster) are associated with invasive GAS disease.[23]		Invasive GAS infection complicates with pneumonia in 23% cases.[23] Case fatality rates are high (23%) in children with invasive GAS-associated pneumonia.[23]
Gram Negative bacteria<br>• Fermenters<br>o *Klebsiella pneumoniae*<br>o *Escherichia coli*	Radiologically-confirmed pneumonia, fever, elevated CRP. Occasionally presents with pleural effusion/ empyema.	Children with HIV, children with chronic lung disease.	Foreign body aspiration is an important contributor to Gram negative lobar pneumonia and	*Klebsiella pneumoniae* was the commonest bacterial organism identified in children dying from

○ *Enterobacter cloacae*<br>○ Salmonella spp.			empyema in older children.[21, 22]	community-acquired pneumonia in the CHAMPS study.[24]
Gram Negative bacteria<br>• Non-fermenters<br>○ *Moraxella catarrhalis*<br>○ *Pseudomonas aeruginosa*<br>○ *Burkholderia cepacia*	*Moraxella catarrhalis* is a common cause of otitis media.[3]<br>Radiologically-confirmed pneumonia, high CRP.	High-grade fever, tachypnoea and high CRP are associated with severe *P. aeruginosa* pneumonia.[25]<br>*B. cepacia* is a rare, but important cause of pneumonia and persistent infection in children with immunodeficiencies.[26]		*M. catarrhalis* is commonly identified in respiratory secretions of previously healthy children hospitalised with CAP.[27]
Bordetella pertussis	Paroxysmal cough, followed by a whoop; apnoea in young infants; post-tussive vomiting.	Young infants too young to have completed a primary series of vaccination against pertussis.	There is conflicting evidence as to the role that HIV plays in promoting clinically severe disease caused by *B. pertussis*.[19, 28, 29]	*B. pertussis* infection in young infants has a high mortality and mortality rate.[30, 31]<br>Suspected cases should be investigated appropriately, and definitive therapy must not be withheld pending results of investigations.<br>Pertussis is a Category 1 Notifiable Medical Condition in South Africa, and as such should be notified within 24 hours of diagnosis.[32]

Chlamydophila pneumoniae	Usually associated with non-severe pneumonia.	Uncommon cause of hospitalised pneumonia in South African children.[33]		
Legionella spp	Non-specific syndrome (termed "Pontiac Fever") of high-grade fever, myalgia and headache which resolves in 3-5 days without specific therapy may go undiagnosed.[34]<br>Occasionally presents as severe pneumonia not responding to conventional antibiotic therapy.	Uncommon cause of pneumonia in children, although case series and reports have been published.[35, 36]	Water births have been ascribed as being a risk factor for Legionnaires' disease in neonates in developed world settings.[37, 38]	If encountered in the clinical setting, Legionnaires' disease must be notified (Category 2 Notifiable Medical Condition).[16]
Mycoplasma pneumoniae	Usually associated with non-severe pneumonia.	Uncommon cause of hospitalised pneumonia in South African children.[39]	Age <5 years and HIV-infection are associated with severe disease.[40]	Meta-analyses suggest that there is insufficient evidence to justify the use of macrolides in the treatment of children with non-severe pneumonia associated with *M. pneumoniae*.[41, 42]
Mycobacterium tuberculosis	Frequently present acutely, with symptoms <14 days in duration. Also, frequently associated with bacterial or viral respiratory co-infection.[43]<br>No chest radiographic picture is diagnostic, but	CLWH and HEU children are at greatest risk for tuberculosis, however due to the burden of HIV and tuberculosis in South Africa *M. tuberculosis* is frequently isolated in HIV-unexposed children.	In pre-school aged children, a household contact with infectious tuberculosis must be sought.[48]<br>Community-acquired tuberculosis is more commonly seen in	Refer to South African National Tuberculosis Guidelines for management of tuberculosis in children.[54]

	suggestive radiographic features include widening of the mediastinum, bronchial compression by enlarge lymph nodes, and miliary pattern.	Malnutrition is an important risk factor for tuberculosis in children.[44] Other medical conditions, including insulin dependent diabetes mellitus,[45] nephrotic syndrome[46] and cancer[47] are also important risk factors.	children who use public transport systems,[49] and those that attend crèche or school.[50-52] Institutional outbreaks occur, e.g. in children's homes. Low socioeconomic status remains a crucial determinant of the burden of tuberculosis.[53]	When encountered in the clinical setting, tuberculosis must be notified (Category 2 Notifiable Medical Condition).[16]

Abbreviations: ART = antiretroviral therapy; CAP = community-acquired pneumonia; CLWH = children living with HIV; CRP = C-reactive protein; GAS = Group A Streptococcus; HEU = HIV-exposed, -uninfected; Hib = *Haemophilus influenzae* type b; HibCV = *Haemophilus influenzae* type b conjugate vaccine; HIV = human immunodeficiency virus type-1; PCP = pneumocystis pneumonia; PCV = pneumococcal conjugate vaccine; PTB = pulmonary tuberculosis.

Supplementary Table 2: Respiratory Viral Pathogens Implicated in Childhood Community-acquired Pneumonia				
Pathogen	Clinical Features	Populations at Risk	Other Risk Factors	Comments
Respiratory syncytial virus (RSV)	Bronchiolitis classically presents as a wheeze-associated lower respiratory tract infection in children <2 years of age.[55, 56] The radiographic picture is that of patchy infiltrates and, occasionally, lobar consolidation. Infants with severe disease are frequently well-grown, or obese for age.[57]<br>HIV-infected infants tend to present with bronchopneumonia, rather than bronchiolitis, when infected with RSV with crackles rather than wheezes on chest auscultation.[58, 59]	Ex-premature (≤35 weeks gestational age) children with chronic lung disease, and infants with haemodynamically significant congenital heart disease are at greatest risk for severe RSV-associated lower respiratory tract infection necessitating ventilation.<br>CLWH and HEU children are at greater risk for hospitalisation with RSV-associated lower respiratory tract infection.[60, 61]	Second hand smoke exposure, low birth weight, male sex, lack of breastfeeding, crowding and sibling crèche attendance are important general risk factors for RSV infection.[62]	RSV-associated lower respiratory tract illness has a strong seasonal association, with the RSV "respiratory season" in South Africa extending from February through August.[63]
Human rhinovirus	Usually implicated in mild upper respiratory tract infection, but occasionally found as an important pneumonia pathogen often as a co-infection with bacteria or other respiratory viruses.<br>Rhinovirus C has been linked to wheeze-associated lower respiratory tract infection in children <5 years of age.	CLWH and HEU children are at risk for rhinovirus-associated hospitalisation.[61]	Important risk factors for virus-associated lower respiratory tract infections include: siblings in the household, smokers in the household, and lack of breastfeeding.[64]<br>Crèche attendance and eosinophilia have been specifically linked to rhinovirus-	Human rhinovirus infection has been shown to facilitate transmission and acquisition of *S. pneumoniae* in families.[65]

			associated lower respiratory tract infection.[64]	
Parainfluenza virus	Typically causes upper respiratory tract infection and mild disease.[66] Laryngotracheobronchitis (croup): fever, barking cough, stridor. Occasionally associated with severe upper airway obstruction, necessitating emergency intubation, and mechanical ventilation.[67]	Infection with parainfluenza virus types 1 and 3 is linked to more severe disease than is infection with parainfluenza virus 2 and 4. Young age, and narrow tracheal diameter, is a risk for severe airways obstruction. HEU are at greater risk for severe disease requiring hospitalisation than are HIV-unexposed children.[61]	Important risk factors for virus-associated lower respiratory tract infections include: siblings in the household, smokers in the household, and lack of breastfeeding.[64]	Clinical severity scores, such as the Westley score are useful in the evaluation of children with croup.[67, 68] The majority of children with croup can be safely managed at home if family circumstances permit.[69]
Influenza virus	Typically causes upper respiratory tract infection and mild disease. Associated with severe pneumonia hospitalisation in young children, which may be complicated by bacterial co-infection.	HIV-infection and exposure are important risk factors for influenza-associated hospitalisation in South African children.[60, 61] Prematurity, age <2 years, immunosuppression and diabetes are important risk factors for influenza-associated hospitalisation.[70]	Important risk factors for virus-associated lower respiratory tract infections include: siblings in the household, smokers in the household, and lack of breastfeeding.[64] Asthma and underlying neurological conditions were risk	Highly seasonal epidemics occur, with the “flu season” in South Africa typically spanning from June through July, and lasting 10 weeks (Range 6-18 weeks).[72] Influenza cases occurring within the context of a global pandemic (i.e. due to

			factors for influenza-associated hospitalisation in children residing in the United Kingdom during the course of the 2009/2010 H1N1 influenza pandemic.[71]	a novel influenza A virus) is a Category 1 Notifiable Medical Condition in South Africa, and as such should be notified within 24 hours of diagnosis.
Human bocavirus	Frequently detected in children with wheeze-associated, radiologically-confirmed lower respiratory tract infection in young children.[73, 74]<br>Mean CRP was noted to be uncharacteristically high for a viral infection (67±79 mg/dL) amongst 80 children with single-pathogen infection in a Spanish time series report.[74]	Children with human bocavirus-associated lower respiratory tract infection tend to be older than those with disease attributable to other respiratory viruses.[74]	Associated with severe pneumonia hospitalisation in HIV-uninfected children in whom no other pathogen was detected.[75, 76]	Implicated as an emerging diarrhoea-causing pathogen in African children with and without lower respiratory tract infection.[77]
Adenovirus	Typically causes mild disease, with upper respiratory tract symptoms, conjunctivitis and occasionally gastrointestinal symptoms.[78]<br>Occasionally causes severe pneumonia, requiring hospitalisation.[78, 79]	CLWH and HEU children are at greater risk for hospitalisation with adenovirus-associated lower respiratory tract infection than are HIV-unexposed children.[58, 61, 80]	Severe disease requiring ICU admission and concomitant bacteraemia are independently associated with mortality in South African children with adenovirus-associated pneumonia.[79]	

Human metapneumovirus (HMPV)	Wide spectrum of clinical illness, from mild upper respiratory tract infection, to bronchiolitis and pneumonia.[81]<br>An important cause of wheeze-associate lower respiratory tract infection, necessitating hospitalisation in young children.	CLWH and HEU children have a higher burden of hospitalisation related to HMPV than do HIV-uninfected children.[61, 82]<br>Higher rates of concomitant bacteraemia and mortality have been noted in CLWH with HMPV-associated lower respiratory tract infection, compared to HIV-uninfected children.[82]	Important risk factors for virus-associated lower respiratory tract infections include: malnutrition, low birth weight, lack of breastfeeding, exposure to solid fuels in the household, and crowding.[83]	HMPV was first discovered in 2001 in the Netherlands.[81]<br>Peak seasonality of HMPV in South Africa is in the autumn and winter months (April through August).[82]
Human coronavirus	Usually cause mild upper respiratory tract infections.<br>Occasionally implicated in severe pneumonia requiring hospitalisation in children, especially in cases that test negative for the 'big six' respiratory viruses (RSV, HMPV, rhinovirus, influenza virus, parainfluenza virus, adenovirus).[84]	Younger children are at higher risk for severe coronavirus-associated lower respiratory tract infection necessitating hospitalisation.[85]	Important risk factors for virus-associated lower respiratory tract infections include: siblings in the household, smokers in the household, and lack of breastfeeding.[64]<br>History of travel to countries experiencing epidemics of severe acute respiratory infection in patients presenting with ARDS should prompt immediate critical care support, isolation, and work-up for	Coronavirus cases occurring within the context of an outbreak ascribed to a novel respiratory pathogen (e.g. MERS-coronavirus, SARS-coronavirus or COVID-2019) is a Category 1 Notifiable Medical Condition in South Africa, and as such should be notified within 24 hours of diagnosis.

			disease caused by a novel coronavirus.[86]	
Parechovirus/ enterovirus	Usually implicated in mild upper respiratory tract infection, including otitis media.[87]<br>Uncommonly causes severe wheeze-associated lower respiratory tract infection necessitating hospitalisation.[88]			Enterovirus-68 is an emerging pathogen, associated with outbreaks of wheeze-associated respiratory illness requiring hospitalisation.[88, 89]<br>Acute flaccid paralysis has also been identified in children recovering from Enterovirus-68 infection.[90]
Measles	An acute, febrile illness (≥38.3°C) associated with an erythematous maculopapular rash, characterised by the presence of at least one of the three "Cs":<br>o cough,<br>o coryza or<br>o conjunctivitis.[91]<br>Otitis media and croup may occur during the course of illness, but pneumonia is the most frequent severe complication of the infection, and has high associated morbidity and mortality.[91]	Malnourished and immunocompromised children are at greatest risk for severe measles.[91]<br>Measles pneumonia is usually caused by secondary viral or bacterial infection.[91]	42% of all measles-related deaths in 2016 occurred in Africa.[92]	Measles is the most infectious human pathogen known, with a basic reproduction number (R_0) of 13.7 to 18.0.[93] Children with suspected or confirmed measles must be nursed in isolation.<br>Measles is a Category 1 Notifiable Medical Condition in South Africa, and as such

				should be notified within 24 hours of diagnosis.[32]
Varicella-zoster	Usually causes a benign illness with fever, generalised pruritic vesicular skin rash, and a vesicular enanthem. Pneumonia develops in 2-10% of varicella-zoster cases.[94]	Varicella-zoster pneumonia may occur in malnourished, HIV-infected, or otherwise immunocompromised children.[94]		The R_0 for varicella-zoster virus is 9.0.[93] Children with varicella or zoster must be nursed in isolation.

Abbreviations: ARDS = acute respiratory distress syndrome; CLWH = children living with HIV; COVID-2019 = Coronavirus Disease 2019; HEU = HIV-exposed, -uninfected; HIV = human immunodeficiency virus type-1; HMPV = human metapneumovirus; MERS = Middle-East Respiratory Syndrome; R_0 = basic reproduction number; RSV = respiratory syncytial virus; SARS = severe acute respiratory syndrome.

References

1. Marom T, Tan A, Wilkinson GS, Pierson KS, Freeman JL, Chonmaitree T. Trends in otitis media-related health care use in the United States, 2001-2011. *JAMA Pediatr*. 2014;168(1):68-75. https://doi.org/10.1001/jamapediatrics.2013.3924

2. Van Dyke MK, Pirçon J-Y, Cohen R, et al. Etiology of Acute Otitis Media in Children Less Than 5 Years of Age: A Pooled Analysis of 10 Similarly Designed Observational Studies. *Pediatr Infect Dis J*. 2017;36(3):274-281. https://doi.org/10.1097/INF.0000000000001420

3. Madhi SA, Govender N, Dayal K, et al. Bacterial and Respiratory Viral Interactions in the Etiology of Acute Otitis Media in HIV-infected and HIV-uninfected South African Children. *Pediatr Infect Dis J*. 2015;34(7):753-760. https://doi.org/10.1097/INF.0000000000000733

4. Higdon MM, Le T, O'Brien KL, et al. Association of C-Reactive Protein With Bacterial and Respiratory Syncytial Virus-Associated Pneumonia Among Children Aged <5 Years in the PERCH Study. *Clin Infect Dis*. 2017;64(suppl_3):S378-s386. https://doi.org/10.1093/cid/cix150

5. von Mollendorf C, Cohen C, de Gouveia L, et al. Risk factors for invasive pneumococcal disease among children less than 5 years of age in a high HIV prevalence setting, South Africa, 2010 to 2012. *Pediatr Infect Dis J*. 2015;34(1):27-34. https://doi.org/10.1097/INF.0000000000000484

6. von Mollendorf C, von Gottberg A, Tempia S, et al. Increased risk for and mortality from invasive pneumococcal disease in HIV-exposed but uninfected infants aged <1 year in South Africa, 2009-2013. *Clin Infect Dis*. 2015;60(9):1346-1356. https://doi.org/10.1093/cid/civ059

7. Verani JR, Groome MJ, Zar HJ, et al. Risk Factors for Presumed Bacterial Pneumonia Among HIV-uninfected Children Hospitalized in Soweto, South Africa. *Pediatr Infect Dis J*. 2016;35(11):1169-1174. https://doi.org/10.1097/INF.0000000000001264

8. Moore DP, Klugman KP, Madhi SA. Role of Streptococcus pneumoniae in hospitalization for acute community-acquired pneumonia associated with culture-confirmed Mycobacterium tuberculosis in children: a pneumococcal conjugate vaccine probe study. *Pediatr Infect Dis J*. 2010;29(12):1099-1004.

9. O'Brien KL, Wolfson LJ, Watt JP, et al. Burden of disease caused by Streptococcus pneumoniae in children younger than 5 years: global estimates. *Lancet*. 2009;374(9693):893-902. https://doi.org/10.1016/S0140-6736(09)61204-6

10. Klein MR. Infections of the Oropharynx. *Emerg Med Clin North Am*. 2019;37(1):69-80. https://doi.org/10.1016/j.emc.2018.09.002

11. Levy C, Varon E, Ouldali N, et al. Bacterial causes of otitis media with spontaneous perforation of the tympanic membrane in the era of 13 valent pneumococcal conjugate vaccine. *PloS One*. 2019;14(2):e0211712-e0211712. https://doi.org/10.1371/journal.pone.0211712

12. Soeters HM, Blain A, Pondo T, et al. Current Epidemiology and Trends in Invasive Haemophilus influenzae Disease-United States, 2009-2015. *Clin Infect Dis*. 2018;67(6):881-889. https://doi.org/10.1093/cid/ciy187

13. Madhi SA, Petersen K, Madhi A, Khoosal M, Klugman KP. Increased disease burden and antibiotic resistance of bacteria causing severe community-acquired lower respiratory tract infections in human immunodeficiency virus type 1-infected children. *Clin Infect Dis*. 2000;31(1):170-176. https://doi.org/10.1086/313925

14. Madhi SA, Petersen K, Khoosal M, et al. Reduced effectiveness of Haemophilus influenzae type b conjugate vaccine in children with a high prevalence of human immunodeficiency virus type 1 infection. *Pediatr Infect Dis J*. 2002;21(4):315-321. https://doi.org/10.1097/00006454-200204000-00011

15. von Gottberg A, de Gouveia L, Madhi SA, et al. Impact of conjugate Haemophilus influenzae type b (Hib) vaccine introduction in South Africa. *Bull World Health Organ*. 2006;84(10):811-818. https://doi.org/10.2471/blt.06.030361

16. National Institute for Communicable Diseases. Category 2 Notifiable Medical Conditions. National Institute for Communicable Diseases; 2018.

17. Zampoli M, Kappos A, Wolter N, et al. Etiology and Incidence of Pleural Empyema in South African Children. *Pediatr Infect Dis J*. 2015;34(12):1305-1310. https://doi.org/10.1097/INF.0000000000000880

18. Ghoor A, Mabaso T, Mopeli K, et al. Empyema in children hospitalised at Chris Hani Baragwanath Academic Hospital, Johannesburg, South Africa: A retrospective study. *S Afr Med J*. 2018;108(12):1055-1058. https://doi.org/10.7196/SAMJ.2018.v108i12.13099

19. Moore DP, Baillie VL, Mudau A, et al. The etiology of pneumonia in HIV-1-infected South African children: Findings from the Pneumonia Etiology Research for Child Health (PERCH) Study. *In press*. 2020.

20. Verwey C, Velaphi S, Khan R. Bacteria isolated from the airways of paediatric patients with bronchiectasis according to HIV status. *S Afr Med J*. 2017;107(5):435-439. https://doi.org/10.7196/SAMJ.2017.v107i5.10692

21. Li L, Lang Y, Chen N, Shen B. The results and drug susceptibility of respiratory secretion culture of children with trachea bronchial foreign bodies. *Lin Chung Er Bi Yan Hou Tou Jing Wai Ke Za Zhi*. 2016;30(5):389-395.

22. Xie LS, Jiang YZ, Li Q. Experience in children airway foreign bodies: an analysis of 220 cases. *Lin Chung Er Bi Yan Hou Tou Jing Wai Ke Za Zhi*. 2016;30(18):1479-1482. https://doi.org/10.13201/j.issn.1001-1781.2016.18.013

23. Seale AC, Davies MR, Anampiu K, et al. Invasive Group A Streptococcus Infection among Children, Rural Kenya. *Emerg Infect Dis*. 2016;22(2):224-232. https://doi.org/10.3201/eid2202.151358

24. Chawana R, Baillie V, Izu A, et al. Potential of Minimally Invasive Tissue Sampling for Attributing Specific Causes of Childhood Deaths in South Africa: A Pilot, Epidemiological Study. *Clin Infect Dis*. 2019;69(Suppl 4):S361-S373. https://doi.org/10.1093/cid/ciz550

25. Chen J, Chen Y, Hu P, Zhou T, Xu X, Pei X. Risk assessment of infected children with Pseudomonas aeruginosa pneumonia by combining host and pathogen predictors. *Infect Genet Evol*. 2018;57:82-87. https://doi.org/10.1016/j.meegid.2017.11.015

26. Lewis ERG, Torres AG. The art of persistence-the secrets to Burkholderia chronic infections. *Pathog Dis*. 2016;74(6):ftw070. https://doi.org/10.1093/femspd/ftw070

27. Sy MG, Robinson JL. Community-acquired Moraxella catarrhalis pneumonia in previously healthy children. *Pediatr Pulmonol*. 2010;45(7):674-678. https://doi.org/10.1002/ppul.21243

28. Muloiwa R, Dube FS, Nicol MP, Zar HJ, Hussey GD. Incidence and Diagnosis of Pertussis in South African Children Hospitalized With Lower Respiratory Tract Infection. *Pediatr Infect Dis J*. 2016;35(6):611-616. https://doi.org/10.1097/inf.0000000000001132

29. Moore DP, Baillie VL, Mudau A, et al. The etiology of pneumonia in HIV-uninfected South African children: Findings from the Pneumonia Etiology Research for Child Health (PERCH) Study. *In press*. 2020.

30. Barger-Kamate B, Deloria Knoll M, Kagucia EW, et al. Pertussis-Associated Pneumonia in Infants and Children From Low- and Middle-Income Countries Participating in the PERCH Study. *Clin Infect Dis*. 2016;63(suppl 4):S187-s196. https://doi.org/10.1093/cid/ciw546

31. Soofie N, Nunes MC, Kgagudi P, et al. The Burden of Pertussis Hospitalization in HIV-Exposed and HIV-Unexposed South African Infants. *Clin Infect Dis*. 2016;63(suppl 4):S165-s173. https://doi.org/10.1093/cid/ciw545

32. National Institute for Communicable Diseases. Category 1 Notifiable Medical Conditions. National Institute for Communicable Diseases; 2018.

33. Carrim M, Wolter N, Benitez AJ, et al. Epidemiology and Molecular Identification and Characterization of Mycoplasma pneumoniae, South Africa, 2012-2015. *Emerg Infect Dis*. 2018;24(3):506-513. https://doi.org/10.3201/eid2403.162052

34. Soda EA, Barskey AE, Shah PP, et al. Vital Signs: Health Care-Associated Legionnaires' Disease Surveillance Data from 20 States and a Large Metropolitan Area - United States, 2015. *MMWR Morb Mortal Wkly Rep*. 2017;66(22):584-589. https://doi.org/10.15585/mmwr.mm6622e1

35. Wolter N, Carrim M, Cohen C, et al. Legionnaires' Disease in South Africa, 2012-2014. *Emerg Infect Dis*. 2016;22(1):131-133. https://doi.org/10.3201/eid2201.150972

36. Wolter N, Stewart R. Legionella infection in a hospitalised neonate. In: National Institute for Communicable Diseases, ed. *National Diseases Communiqué*. Sandringham: National Institute for Communicable Diseases; 2016:6-7.

37. Franzin L, Cabodi D, Scolfaro C, Gioannini P. Microbiological investigations on a nosocomial case of Legionella pneumophila pneumonia associated with water birth and review of neonatal cases. *Infez Med*. 2004;12(1):69-75.

38. Collins SL, Afshar B, Walker JT, et al. Heated birthing pools as a source of Legionnaires' disease. *Epidemiol Infect*. 2016;144(4):796-802. https://doi.org/10.1017/S0950268815001983

39. Carrim M. Identification and prevalence of bacteria causing atypical pneumonia in patients with severe respiratory illness and influenza-like illness in South Africa, 2012-2013.

Faculty of Health Sciences. Johannesburg: University of the Witwatersrand; 2015:108. Available at: http://wiredspace.wits.ac.za/handle/10539/19566

40. Maimuna C, Nicole W, Alvaro JB, et al. Epidemiology and Molecular Identification and Characterization of Mycoplasma pneumoniae, South Africa, 2012–2015. *Emerg Infect Dis*. 2018;24(3):506. https://doi.org/10.3201/eid2403.162052

41. Gardiner SJ, Gavranich JB, Chang AB. Antibiotics for community-acquired lower respiratory tract infections secondary to Mycoplasma pneumoniae in children. *Cochrane Database Syst Rev*. 2015;1:CD004875. https://doi.org/10.1002/14651858.CD004875.pub5

42. Blyth CC, Gerber JS. Macrolides in Children With Community-Acquired Pneumonia: Panacea or Placebo? *J Pediatric Infect Dis Soc*. 2018;7(1):71-77. https://doi.org/10.1093/jpids/pix083

43. Oliwa JN, Karumbi JM, Marais BJ, Madhi SA, Graham SM. Tuberculosis as a cause or comorbidity of childhood pneumonia in tuberculosis-endemic areas: a systematic review. *The Lancet Respir Med*. 2015;3(3):235-243. https://doi.org/10.1016/S2213-2600(15)00028-4

44. Adler H, Archary M, Mahabeer P, LaRussa P, Bobat RA. Tuberculosis in HIV-infected South African children with complicated severe acute malnutrition. *Int J Tuberc Lung Dis.* 2017;21(4):438-445. https://doi.org/10.5588/ijtld.16.0753

45. Webb EA, Hesseling AC, Schaaf HS, et al. High prevalence of Mycobacterium tuberculosis infection and disease in children and adolescents with type 1 diabetes mellitus. *Int J Tuberc Lung Dis*. 2009;13(7):868-874.

46. Kala U, Milner LS, Jacobs D, Thomson PD. Impact of tuberculosis in children with idiopathic nephrotic syndrome. *Pediatr Nephrol*. 1993;7(4):392-395. https://doi.org/10.1007/bf00857548

47. Naidu G. Infectious Complications in the South African Black Child with Cancer. *Faculty of Health Sciences*. Johannesburg: University of the Witwatersrand; 2016:236. Available at: http://wiredspace.wits.ac.za/handle/10539/22523

48. Wood R, Johnstone-Robertson S, Uys P, et al. Tuberculosis transmission to young children in a South African community: modeling household and community infection risks. *Clin Infect Dis*. 2010;51(4):401-408. https://doi.org/10.1086/655129

49. Andrews JR, Morrow C, Wood R. Modeling the role of public transportation in sustaining tuberculosis transmission in South Africa. *Am J Epidemiol*. 2013;177(6):556-561. https://doi.org/10.1093/aje/kws331

50. Wood R, Racow K, Bekker L-G, et al. Indoor social networks in a South African township: potential contribution of location to tuberculosis transmission. *PloS One*. 2012;7(6):e39246-e39246. https://doi.org/10.1371/journal.pone.0039246

51. Churchyard G, Kim P, Shah NS, et al. What We Know About Tuberculosis Transmission: An Overview. *J Infect Dis*. 2017;216(suppl_6):S629-S635. https://doi.org/10.1093/infdis/jix362

52. Schepisi MS, Motta I, Dore S, Costa C, Sotgiu G, Girardi E. Tuberculosis transmission among children and adolescents in schools and other congregate settings: a systematic review. *New Microbiol*. 2019;41(4):282-290.

53. Yates TA, Ayles H, Leacy FP, et al. Socio-economic gradients in prevalent tuberculosis in Zambia and the Western Cape of South Africa. *Trop Med Int Health*. 2018;23(4):375-390. https://doi.org/10.1111/tmi.13038

54. National Department of Health SA. Guidelines for the Management of Tuberculosis in Children. 2013. 2013.

55. Resch B, Kurath-Koller S, Eibisberger M, Zenz W. Prematurity and the burden of influenza and respiratory syncytial virus disease. *World J Pediatr*. 2016;12(1):8-18. https://doi.org/10.1007/s12519-015-0055-x

56. Resch B. Product review on the monoclonal antibody palivizumab for prevention of respiratory syncytial virus infection. *Hum Vaccin Immunother*. 2017;13(9):2138-2149. https://doi.org/10.1080/21645515.2017.1337614

57. Akiyama N, Segawa T, Ida H, et al. Bimodal effects of obesity ratio on disease duration of respiratory syncytial virus infection in children. *Allergol Int*. 2011;60(3):305-308. https://doi.org/10.2332/allergolint.10-OA-0252

58. Madhi SA, Schoub B, Simmank K, Blackburn N, Klugman KP. Increased burden of respiratory viral associated severe lower respiratory tract infections in children infected with human immunodeficiency virus type-1. *J Pediatr*. 2000;137(1):78-84. https://doi.org/10.1067/mpd.2000.105350

59. Annamalay AA, Abbott S, Sikazwe C, et al. Respiratory viruses in young South African children with acute lower respiratory infections and interactions with HIV. *J Clin Virol*. 2016;81:58-63. https://doi.org/10.1016/j.jcv.2016.06.002

60. McMorrow ML, Tempia S, Walaza S, et al. The Role of Human Immunodeficiency Virus in Influenza- and Respiratory Syncytial Virus-associated Hospitalizations in South African Children, 2011-2016. *Clin Infect Dis*. 2019;68(5):773-780. https://doi.org/10.1093/cid/ciy532

61. Cohen C, Moyes J, Tempia S, et al. Epidemiology of Acute Lower Respiratory Tract Infection in HIV-Exposed Uninfected Infants. *Pediatrics*. 2016;137(4):e20153272. https://doi.org/10.1542/peds.2015-3272

62. Shi T, Balsells E, Wastnedge E, et al. Risk factors for respiratory syncytial virus associated with acute lower respiratory infection in children under five years: Systematic

review and meta-analysis. *J Glob Health*. 2015;5(2):020416-020416. https://doi.org/10.7189/jogh.05.020416

63. Obando-Pacheco P, Justicia-Grande AJ, Rivero-Calle I, et al. Respiratory Syncytial Virus Seasonality: A Global Overview. *J Infect Dis*. 2018;217(9):1356-1364. https://doi.org/10.1093/infdis/jiy056

64. Nicolai A, Frassanito A, Nenna R, et al. Risk Factors for Virus-induced Acute Respiratory Tract Infections in Children Younger Than 3 Years and Recurrent Wheezing at 36 Months Follow-Up After Discharge. *Pediatr Infect Dis J*. 2017;36(2):179-183. https://doi.org/10.1097/INF.0000000000001385

65. Karppinen S, Teräsjärvi J, Auranen K, et al. Acquisition and Transmission of Streptococcus pneumoniae Are Facilitated during Rhinovirus Infection in Families with Children. *Am J Respir Crit Care Med*. 2017;196(9):1172-1180. https://doi.org/10.1164/rccm.201702-0357OC

66. Pawełczyk M, Kowalski ML. The Role of Human Parainfluenza Virus Infections in the Immunopathology of the Respiratory Tract. *Curr Allergy Asthma Rep*. 2017;17(3):16-16. https://doi.org/10.1007/s11882-017-0685-2

67. Johnson DW. Croup. *BMJ Clin Evid*. 2014;2014:0321.

68. Branche AR, Falsey AR. Parainfluenza Virus Infection. *Semin Respir Crit Care Med*. 2016;37(4):538-554. https://doi.org/10.1055/s-0036-1584798

69. Yang W-C, Lee J, Chen C-Y, Chang Y-J, Wu H-P. Westley score and clinical factors in predicting the outcome of croup in the pediatric emergency department. *Pediatr Pulmonol*. 2017;52(10):1329-1334. https://doi.org/10.1002/ppul.23738

70. Gill PJ, Ashdown HF, Wang K, et al. Identification of children at risk of influenza-related complications in primary and ambulatory care: a systematic review and meta-analysis. *Lancet Respir Med*. 2015;3(2):139-149. https://doi.org/10.1016/S2213-2600(14)70252-8

71. Lee JJ, Bankhead C, Smith M, Kousoulis AA, Butler CC, Wang K. Risk factors for influenza-related complications in children during the 2009/10 pandemic: a UK primary care cohort study using linked routinely collected data. *Epidemiol Infect*. 2018;146(7):817-823. https://doi.org/10.1017/S0950268818000353

72. McAnerney JM, Cohen C, Moyes J, et al. Twenty-five years of outpatient influenza surveillance in South Africa, 1984-2008. *J Infect Dis*. 2012;206 Suppl 1:S153-158. https://doi.org/10.1093/infdis/jis575

73. Calvo C, García-García ML, Pozo F, Carvajal O, Pérez-Breña P, Casas I. Clinical characteristics of human bocavirus infections compared with other respiratory viruses in Spanish children. *Pediatr Infect Dis J*. 2008;27(8):677-680. https://doi.org/10.1097/INF.0b013e31816be052

74. Calvo C, García-García ML, Pozo F, Carballo D, Martínez-Monteserín E, Casas I. Infections and coinfections by respiratory human bocavirus during eight seasons in hospitalized children. *J Med Virol*. 2016;88(12):2052-2058. https://doi.org/10.1002/jmv.24562

75. Schlaberg R, Ampofo K, Tardif KD, et al. Human Bocavirus Capsid Messenger RNA Detection in Children With Pneumonia. *J Infect Dis*. 2017;216(6):688-696. https://doi.org/10.1093/infdis/jix352

76. Schlaberg R, Queen K, Simmon K, et al. Viral Pathogen Detection by Metagenomics and Pan-Viral Group Polymerase Chain Reaction in Children With Pneumonia Lacking Identifiable Etiology. *J Infect Dis*. 2017;215(9):1407-1415. https://doi.org/10.1093/infdis/jix148

77. Rikhotso MC, Kabue JP, Ledwaba SE, Traoré AN, Potgieter N. Prevalence of Human Bocavirus in Africa and Other Developing Countries between 2005 and 2016: A Potential

Emerging Viral Pathogen for Diarrhea. *J Trop Med*. 2018;2018:7875482-7875482.
https://doi.org/10.1155/2018/7875482

78. Lynch JP, 3rd, Kajon AE. Adenovirus: Epidemiology, Global Spread of Novel Serotypes, and Advances in Treatment and Prevention. *Semin Respir Crit Care Med*. 2016;37(4):586-602. https://doi.org/10.1055/s-0036-1584923

79. Zampoli M, Mukuddem-Sablay Z. Adenovirus-associated pneumonia in South African children: Presentation, clinical course and outcome. *S Afr Med J*. 2017;107(2):123-126.
https://doi.org/10.7196/SAMJ.2017.v107i2.11451

80. O'Callaghan-Gordo C, Bassat Q, Morais L, et al. Etiology and epidemiology of viral pneumonia among hospitalized children in rural Mozambique: a malaria endemic area with high prevalence of human immunodeficiency virus. *Pediatr Infect Dis J*. 2011;30(1):39-44.
https://doi.org/10.1097/INF.0b013e3181f232fe

81. van den Hoogen BG, de Jong JC, Groen J, et al. A newly discovered human pneumovirus isolated from young children with respiratory tract disease. *Nat Med*. 2001;7(6):719-724.
https://doi.org/10.1038/89098

82. Madhi SA, Ludewick H, Kuwanda L, van Niekerk N, Cutland C, Klugman KP. Seasonality, incidence, and repeat human metapneumovirus lower respiratory tract infections in an area with a high prevalence of human immunodeficiency virus type-1 infection. *Pediatr Infect Dis J*. 2007;26(8):693-699. https://doi.org/10.1097/INF.0b013e3180621192

83. Rudan I, O'Brien KL, Nair H, et al. Epidemiology and etiology of childhood pneumonia in 2010: estimates of incidence, severe morbidity, mortality, underlying risk factors and causative pathogens for 192 countries. *J Glob Health*. 2013;3(1):010401-010401.
https://doi.org/10.7189/jogh.03.010401

84. Subramoney K, Hellferscee O, Pretorius M, et al. Human bocavirus, coronavirus, and polyomavirus detected among patients hospitalised with severe acute respiratory illness in

South Africa, 2012 to 2013. *Health Sci Rep*. 2018;1(8):e59-e59. https://doi.org/10.1002/hsr2.59

85. Smuts H. Human coronavirus NL63 infections in infants hospitalised with acute respiratory tract infections in South Africa. *Influenza Other Respir Viruses*. 2008;2(4):135-138. https://doi.org/10.1111/j.1750-2659.2008.00049.x

86. Mackay IM, Arden KE. An Opportunistic Pathogen Afforded Ample Opportunities: Middle East Respiratory Syndrome Coronavirus. *Viruses*. 2017;9(12):369. https://doi.org/10.3390/v9120369

87. Esposito S, Rahamat-Langendoen J, Ascolese B, Senatore L, Castellazzi L, Niesters HGM. Pediatric parechovirus infections. *J Clin Virol*. 2014;60(2):84-89. https://doi.org/10.1016/j.jcv.2014.03.003

88. Schuffenecker I, Mirand A, Josset L, et al. Epidemiological and clinical characteristics of patients infected with enterovirus D68, France, July to December 2014. *Euro Surveill*. 2016;21(19):10.2807/1560-7917.ES.2016.2821.2819.30226. https://doi.org/10.2807/1560-7917.ES.2016.21.19.30226

89. Greninger AL, Naccache SN, Messacar K, et al. A novel outbreak enterovirus D68 strain associated with acute flaccid myelitis cases in the USA (2012-14): a retrospective cohort study. *Lancet Infect Dis*. 2015;15(6):671-682. https://doi.org/10.1016/S1473-3099(15)70093-9

90. Cassidy H, Poelman R, Knoester M, Van Leer-Buter CC, Niesters HGM. Enterovirus D68 - The New Polio? *Front Microbiol*. 2018;9:2677-2677. https://doi.org/10.3389/fmicb.2018.02677

91. Moss WJ. Measles. *Lancet*. 2017;390(10111):2490-2502. https://doi.org/10.1016/s0140-6736(17)31463-0

92. Dabbagh A, Patel MK, Dumolard L, et al. Progress Toward Regional Measles Elimination - Worldwide, 2000-2016. *MMWR Morb Mortal Wkly Rep*. 2017;66(42):1148-1153. https://doi.org/10.15585/mmwr.mm6642a6

93. Anderson RM, May RM. Directly transmitted infections diseases: control by vaccination. *Science*. 1982;215(4536):1053-1060. https://doi.org/10.1126/science.7063839

94. Hussey H, Abdullahi L, Collins J, Muloiwa R, Hussey G, Kagina B. Varicella zoster virus-associated morbidity and mortality in Africa - a systematic review. *BMC Infect Dis*. 2017;17(1):717. https://doi.org/10.1186/s12879-017-2815-9
